# Preparation of Benzo[*a*]fluorenes via Pd-Catalyzed Annulation of 5-(2-Bromophenyl)pent-3-en-1-ynes

**DOI:** 10.1021/acs.joc.4c01286

**Published:** 2024-08-09

**Authors:** Cheng-Kai Hsu, Yi-Hung Liu, Shiuh-Tzung Liu

**Affiliations:** Department of Chemistry, National Taiwan University, Taipei 106, Taiwan

## Abstract

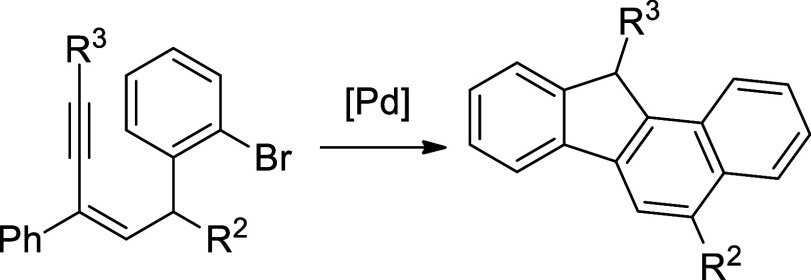

A palladium-promoted cascade cyclization of 5-(2-bromophenyl)pent-3-en-1-ynes is developed for the synthesis of benzo[*a*]fluorene derivatives. The reaction proceeds with oxidative addition of C–Br, insertion, C–H activation, and reductive elimination in sequential steps.

## Introduction

**Scheme 1:**
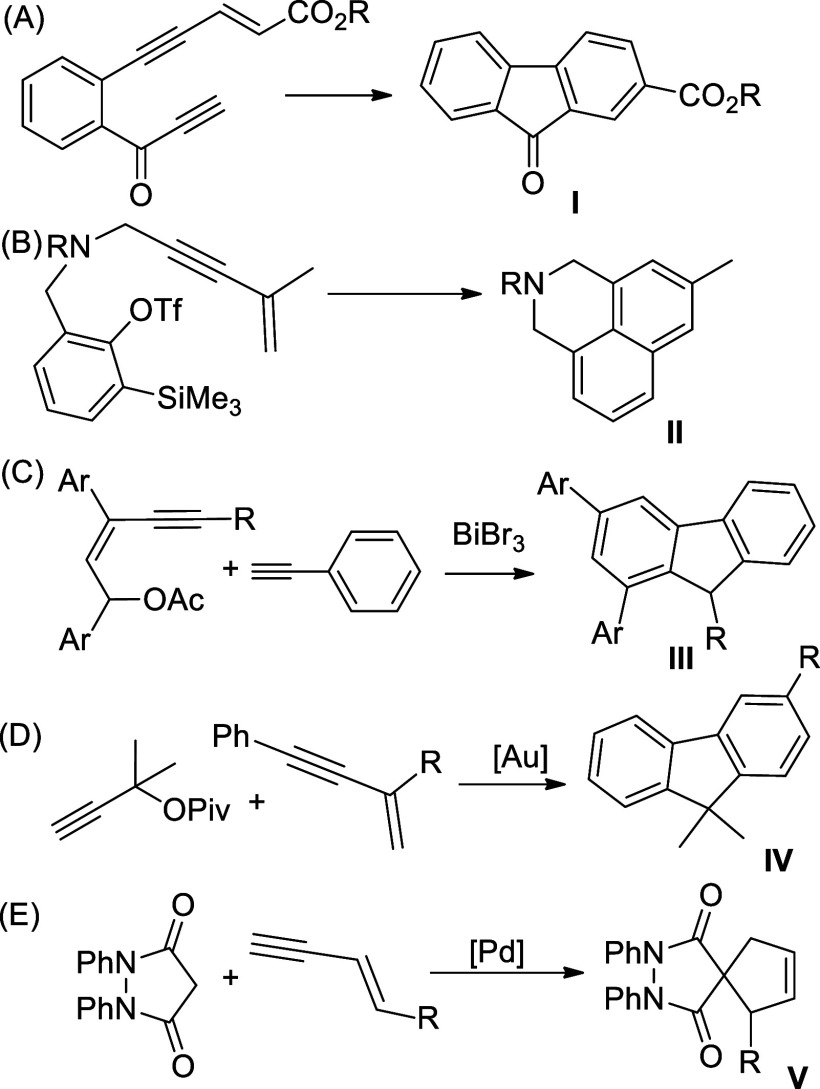


1,3-Enynes are important organic scaffolds and have been implemented in many synthetic applications.^1−7^ Among them, constructions of cyclic compounds from 1,3-enynes have attracted significant interest due to the important application of these obtained molecules.^2^ However, the methodologies leading to polycyclic compounds with the use of four carbon units of 1,3-enynes as part of ring frameworks are somewhat limited and selected examples are summarized in Scheme 1.^3−8^ [4 + 2] Cycloaddition to construct ring systems ought to be an efficient way. Thus, intramolecular cycloaddition of the 1,3-enyne moiety with an alkyne or with *in situ* generated benzyne provided fluoren-9-one **I** and dihydro-1*H*-benzo[*de*]isoquinoline **II**, respectively (Scheme 1A, B).^3,4^ Liang and co-workers discovered a unique preparation of 9-substituted fluorenes **III** via a sequential cyclization of 2-en-4-yn-1-yl acetate with a terminal alkyne in the presence of BiBr_3_.^5^ Transition metal-promoted cyclization of enynes leading to cyclic rings is another useful approach in synthesis. Toste and co-workers reported that Au(I)-catalyzed annulation of aryl-substituted enynes and propargyl esters gave the fluorene products **IV** (Scheme 1D).^6^ Pd-catalyzed [4 + 1] reaction of 1,3-enynes with pyrazolidine-3,5-dione leading spiro compounds **V** was disclosed by Shao’s group (Scheme 1E).^7^

**Scheme 2:**
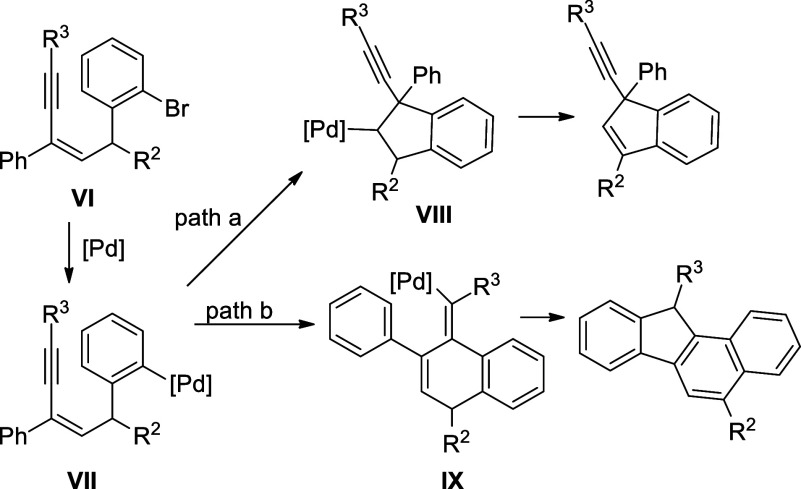
Our Approach in Preparation of Benzofluorenes

We are also interested in the reactivity of 1,3-enyne systems and have reported the preparation of pyrroles, furans, and tetrahydropentalenopyrroles from 1-en-4-yn-3-ol derivatives.^9^ In this work, we anticipated that an enyne **VI** with a pendant bromoaryl group for studying the intramolecular insertion might lead to the cyclization products via Pd-catalyzed cascade reactions (Scheme 2). Activation of C–Br in **VI** gives the Pd-aryl intermediate **VII**, which may undergo the migratory insertion with either ene or yne moiety of the molecule, giving indenyl species **VIII** (Scheme 2, path a) or naphthalenyl species **IX** (Scheme 2, path b), respectively. Subsequently, **VIII** undergoes β-elimination provides indene derivatives, whereas **IX** may proceed C–H activation with the adjacent phenyl ring and render the benzo[*a*]fluorenes.

## Results and Discussion

Substrates in this investigation were prepared by the electrophilic aromatic substitution of *m*-bromoanilines with 2-en-4-yn-1-ol under acidic conditions (eq 1). It is noticed that *m*-bromophenols did not undergo this kind of substitution. In addition, when the R^2^ group is an alkyl group, the substitution reaction gave a complicated mixture of products. To validate our idea, the investigation started with **1a** as the model reaction to establish the optimized catalytic conditions (Table 1).
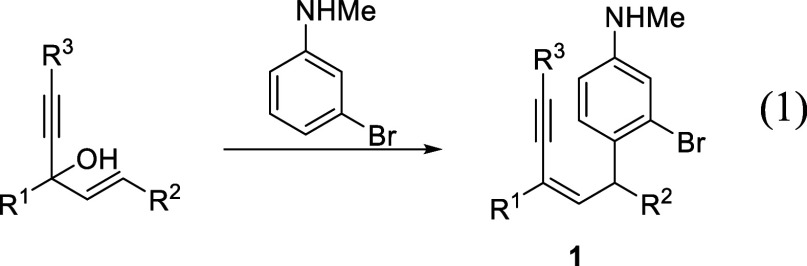
1

**Table 1:**
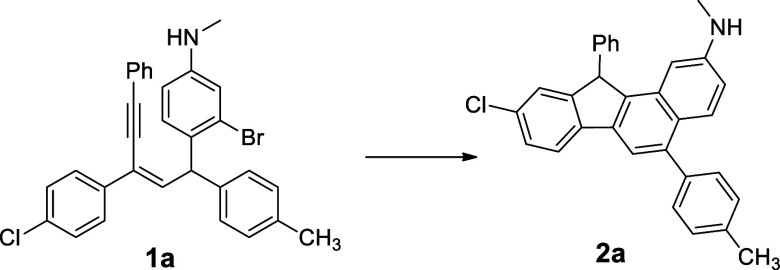
Reaction Optimization^a^

entry	catalyst	base	solvent	yield^b^
1	Pd(PPh_3_)_4_	Bu_3_N^d^	DMF	18%
2	Pd(PPh_3_)_4_	Bu_3_N	DMF	71%
3	Pd(PPh_3_)_4_	KOH	DMF	f
4	Pd(PPh_3_)_4_	Cs_2_CO_3_	DMF	f
5	Pd(OAc)_2_/PPh_3_^c^	Bu_3_N	DMF	23%
6	Pd(OAc)_2_/dppp	Bu_3_N	DMF	54%
7	Pd(OAc)_2_/DPEphos	Bu_3_N	DMF	83%
8	PdCl_2_/DPEphos	Bu_3_N	DMF	30%
9	Pd(OAc)_2_/DPEphos	Bu_3_N	DMSO	f
10	Pd(OAc)_2_/DPEphos	Bu_3_N	xylene	14%
11	Pd(OAc)_2_/DPEphos	Bu_3_N	DMA	f
12^e^	Pd(OAc)_2_/DPEphos	Bu_3_N	DMF	86%

a Reaction conditions: **1a** (0.4 mmol), Pd complex (5 mol %), ligands (6 mol %) and base (1.2 mmol) in 8 mL DMF under N_2_ at 140 °C for 16 h. DPEphos = bis[(2-diphenylphosphino)phenyl] ether.

b Yields were determined by ^1^H NMR with 1,3,5-trimethoxybenzene as the internal standard.

c PPh_3_ (0.04 mmol, 10 mol %).

d 0.6 mmol Bu_3_N.

e 160 °C instead of 140 °C.

f Complicated mixture.

After several trials, we found that the Pd(PPh_3_)_4_-catalyzed reaction of **1a** in the presence of tributylamine in DMF at 140 °C gave the fluorene product **2a** in 18% (entry 1). By increasing the amount of tributylamine to 3 equiv, compound **2a** was obtained in 71% (entry 2). Among various bases, tributylamine gave the best result (entry 2–4). Then, various palladium sources and ligand combination were screened (entries 5–8). To our delight, the use of Pd(OAc)_2_ and DPEphos as the catalytic system provided **2a** in 83% yield (entry 7). Carrying out the reaction in other solvents provided inferior results (entries 9–11). Finally, the yield was improved up to 86% by running the reaction at 160 °C (entry 12). It is also noticed that no indene derivative was detected in this reaction, presumably due to the unfavorable ring strain of the transition state during the insertion.

**Figure 1:**
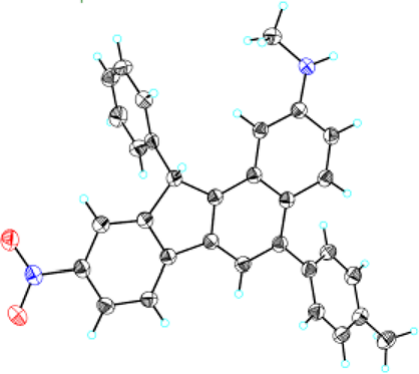
ORTEP plot of **2d** (30% probability ellipsoids).

**Scheme 3:**
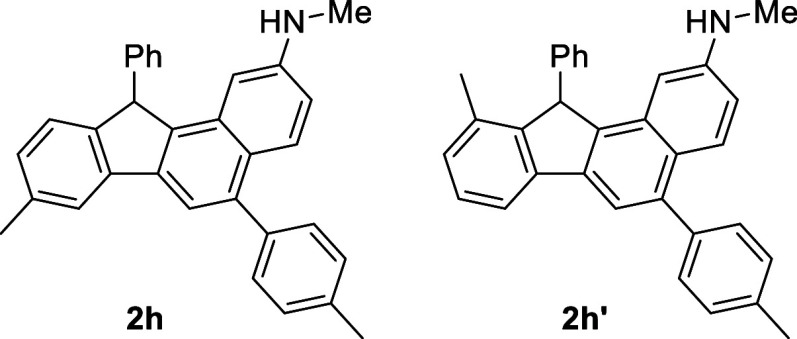
Structures of **2h** and **2h′.**

With the above optimized conditions, exploration of the Pd-catalyzed reaction of various substituents in **1** was investigated and compounds **1** with a variety of aryl groups at positions 1 and 3 underwent the cyclization to render the corresponding fluorenes (Table 2). First, reactants **1a**–**1g** with electron-withdrawing (−F, −NO_2_) or electron-donating (−OCH_3_, −Me) substituents on phenyl rings has no significant effect on the reaction, giving the corresponding products **1a**–**1g** in good to excellent yields except **1c** (entries **1a**–**1g**). Presumably, the extra aryl bromide moiety causes the complication of the reaction. As expected, substrate **1h** with the *meta*-methyl group gave a mixture of regio-isomers (entry **1h**) and the structures are shown in Scheme 3. On the other hand, under the optimized catalyzed conditions, **1j**–**1o** with R^2^ as various aryl groups yielded the corresponding product **2j**–**2o** in excellent yields, respectively, even with a thienyl group (entry **1o**). All products were characterized by NMR and mass spectroscopy. Crystallographic analysis of **2d** was determined, and the proposed structure was confirmed (Figure 1). To further demonstrate the practicality and efficiency of the developed method, gram-scale reactions were performed. Under the optimal conditions, reaction of **1a** (1.05 g, 2 mmol) rendered **2a** in 85% isolating yield.

**Table 2:**
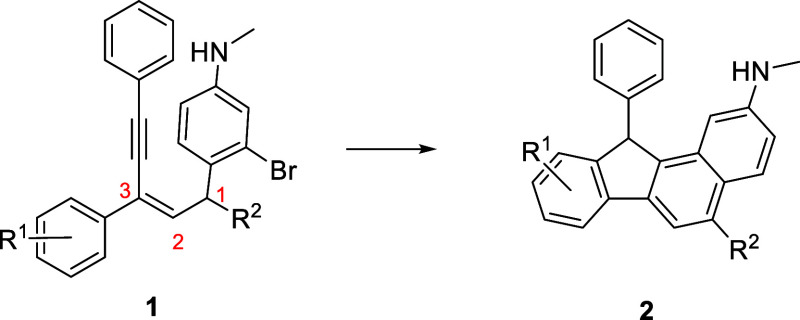
Substrate Scope^a^

reactant	R^1^	R^2^	yield (%)
**1a**	*p*-Cl	*p*-MeC_6_H_4_	**2a** (86%)
**1b**	*p*-F	*p*-MeC_6_H_4_	**2b** (84%)
**1c**	*p*-Br	*p*-MeC_6_H_4_	**2c** (0%)
**1d**	*p*-NO_2_	*p*-MeC_6_H_4_	**2d** (55%)
**1e**	H	*p*-MeC_6_H_4_	**2e** (79%)
**1f**	*p*-MeO	*p*-MeC_6_H_4_	**2f** (76%)
**1g**	*p*-Me	*p*-MeC_6_H_4_	**2g** (83%)
**1h**	*m*-Me	*p*-MeC_6_H_4_	**2h** (42%) + **2h** (30%)^b^
**1i**	*o-*Me	*p*-MeC_6_H_4_	**2i** (48%)
**1j**	*p*-Cl	*p*-ClC_6_H_4_	**2j** (89%)
**1k**	*p*-Cl	*p*-(CF_3_)C_6_H_4_	**2k** (88%)
**1l**	*p*-Cl	*p*-MeOC_6_H_4_	**2l** (78%)
**1m**	*p*-Cl	*m*-MeC_6_H_4_	**2m** (88%)
**1n**	*p*-Cl	*o*-MeC_6_H_4_	**2n** (90%)
**1o**	*p*-Cl	2-thiophenyl	**2o** (80%)

a Reaction conditions: **1** (0.4 mmol), Pd(OAc)_2_ (5 mol %), DPEphos (6 mol %), Bu_3_N (3.0 equiv) in 8 mL of DMF under N_2_ at 160 °C for 16 h; isolated yields.

b Structures given in Scheme 3.

**Scheme 4:**
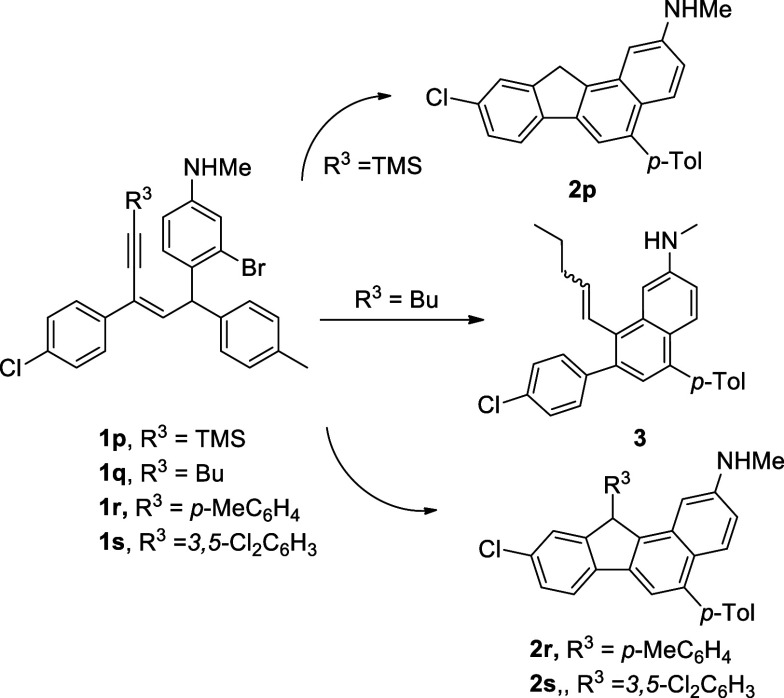
Substituent Effect on the Alkynyl Carbon

The substituent effect on alkynyl carbon was also studied. Four examples with a different substituent at the alkynyl-carbon (**1p**–**1s** with TMS, butyl, *p*-MeC_6_H_4_ and, 3,5-Cl_2_C_6_H_3_, respectively) were investigated, and the results are shown in Scheme 4. Compound **1p** underwent the cyclization to give the benzo[*a*]fluorene product **2p**, but the TMS group was removed during the reaction. When the alkynyl carbon is attaching to a butyl group, a naphthalene product **3** was obtained. Apparently, the β-elimination is superior to the C–H activation. As for substituted phenyl groups at the alkynyl carbon (R^3^ position), reaction of **1r** and **1s** proceeded smoothly giving the desired benzo[*a*]fluorene derivatives **2r** and **2s** in 81 and 73% yields, respectively, showing a slightly substituent effect.

To gain further understanding about the substituent effect of the R^2^ group in **1**, we prepared compound **4** by the treatment of (*E*)-1-(2-bromophenyl)-3-(4-chlorophenyl)-5-(trimethylsilyl)pent-1-en-4-yn-3-ol in methanol under acidic conditions. Under the Pd-catalyzed conditions described above, **4** was transformed into the desired benzofluorene **5** in 51% yield (eq 2), showing an extensive substrate scope in this methodology.
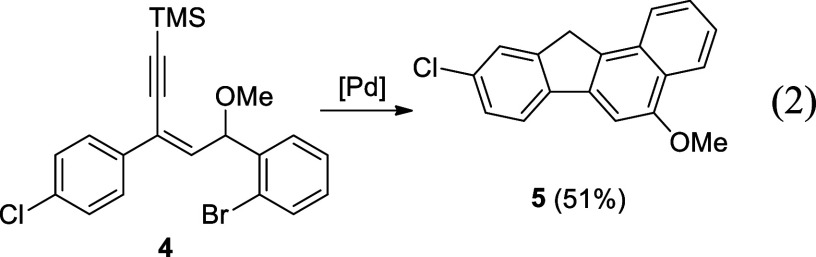
2

**Scheme 5:**
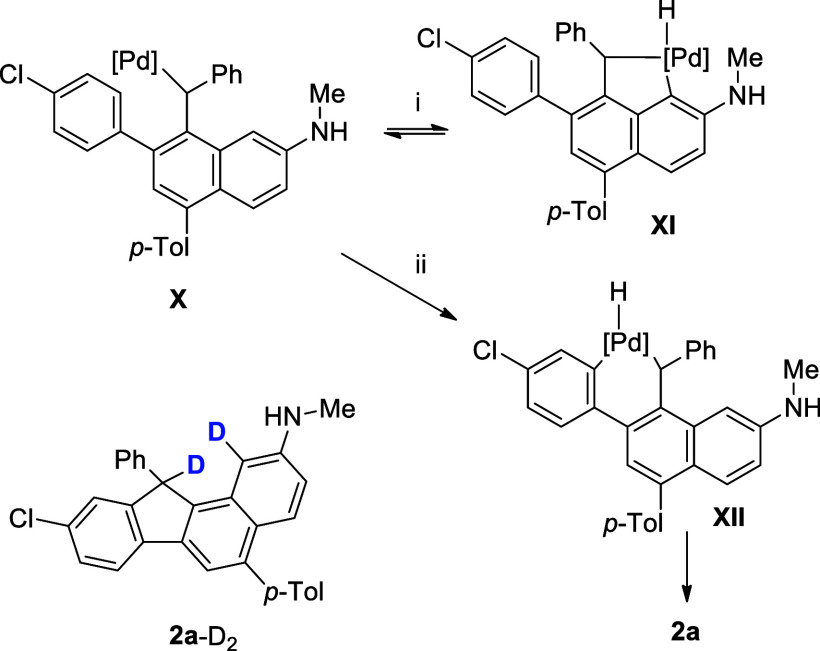
Possible Mechanistic Pathway

As for the mechanistic pathway, we found that carrying out the reaction of **1a** in the presence of trace of D_2_O provided the deuterium-labeled product **2a-**D_2_, suggesting that intermediate **X** might be operating in the reaction. C–H activation could be occurred with the two positions of the two adjacent phenyl rings (Scheme 5). Step i gives a five-membered palladacycle **XI**, which is quite unlikely to undergo the reductive elimination. Step ii is a more favorable process and leads to the final product.

In summary, we have successfully demonstrated an efficient preparation of benzo[*a*]fluorenes from 5-(2-bromophenyl)pent-3-en-1-ynes via Pd-catalyzed activation of C–Br, insertion, C–H activation, and reductive elimination. Further studies involving various aryl rings for further annulation are currently under investigation.

## Experimental Section

### General Information

^1^H and ^13^C NMR were recorded in a 400 MH_Z_ spectrometer in CDCl_3_ referenced to TMS. All chemicals were commercially purchased and used without further purification. Flash chromatography was performed using silica gel 230–400 mesh. Melting points were determined on a Fargo MP-1D instrument. HRMS was recorded in a Bruker micrOTOF-Q II instrument. Cell parameters were determined by a Bruker AXS D8 VENTURE diffractometer. Pent-1-en-4-yn-3-ols were prepared according our previously reported method.^9b^ All reactions were performed without any special precautions.

### General Procedure for Preparation of (*Z*)-(5-(2-Bromoaryl)pent-3-en-1-ynes **1**

To a solution of pent-1-en-4-yn-3-ols (4 mmol) and 3-bromo-*N*-methylaniline (0.85 mL, 6.8 mmol, 1.7 equiv) in DCM (40 mL) was added dropwise the boron trifluoride-diethyl etherate (0.6 mmol, 15 mol %) with stirring at ice bath temperature for 0.5 h. The reaction mixture was stirred at room temperature for 16 h. The reaction was quenched with saturated aq. NaHCO_3_, and the mixture was extracted with DCM (30 mL × 2). The combined organic layer was washed with water (20 mL × 2) and brine (20 mL), dried over MgSO_4_, and concentrated under reduced pressure. The residue was chromatographed on silica gel with hexane/ethyl acetate (19:1) as the eluent, unless noted.

#### (*Z*)-3-Bromo-4-(3-(4-chlorophenyl)-5-phenyl-1-(*p-*tolyl)-pent-2-en-4-yn-1-yl)-*N*-methylaniline (**1a**)

Yellow oil. (1.85 g, 88%), eluent: hexane/ethyl acetate (19:1). ^1^H NMR (400 MHz, CDCl_3_, 298 K): δ 7.71 (d, *J* = 8.6 Hz, 2H), 7.64–7.61 (m, 2H), 7.42–7.38 (m, 5H), 7.23–7.15 (m, 5H), 6.90 (d, *J* = 2.4 Hz, 1H), 6.87 (d, *J* = 9.8 Hz, 1H), 6.59 (dd, *J* = 8.4, 2.4 Hz, 1H), 6.01 (d, *J* = 9.8 Hz, 1H), 3.74 (s, 1H, broad), 2.84 (s, 3H), 2.39 (s, 3H) ppm; ^13^C{^1^H} NMR (100 MHz): δ 148.9, 140.3, 139.5, 136.4, 135.9, 133.6, 131.7, 130.5, 130.0, 129.2, 128.5, 128.4, 128.3, 127.9, 127.6, 125.6, 123.2, 123.0, 116.1, 112.2, 96.3, 86.3, 50.1, 30.5, 21.0 ppm; HRMS (ESI-TOF): *m*/*z* [M + H]^+^ calcd. for C_31_H_25_^79^BrClN: 526.0932; found 526.0945. HRMS (ESI-TOF): *m*/*z* [M + H]^+^ calcd. for C_31_H_25_^81^BrClN^+^: 528.0913; found 528.0937.

#### (*Z*)-3-Bromo-4-(3-(4-fluorophenyl)-5-phenyl-1-(*p-*tolyl)-pent-2-en-4-yn-1-yl)-*N*-methylaniline (**1b**)

Yellow oil. (1.34 g, 65%), eluent: hexane/ethyl acetate (19:1). ^1^H NMR (400 MHz, CDCl_3_, 298 K): δ 7.78–7.75 (m, 2H), 7.65–7.63 (m, 2H), 7.42–7.40 (m, 3H), 7.25 (d, *J* = 9.8 Hz, 2H), 7.21–7.18 (m, 3H), 7.13 (t, *J* = 8.7 Hz, 2H), 6.93 (d, *J* = 2.4 Hz, 1H), 6.84 (d, *J* = 9.8 Hz, 1H), 6.62 (dd, *J* = 8.4, 2.4 Hz, 1H), 6.03 (d, *J* = 9.8 Hz, 1H), 2.84 (s, 3H), 2.40 (s, 3H) ppm; ^13^C{^1^H} NMR (100 MHz): δ 162.5 (d, *J* = 247.3 Hz), 148.7, 140.4, 139.0, 135.8, 134.1 (d, *J* = 3.6 Hz), 131.7, 130.8, 130.1, 129.2, 128.3, 128.2 (d, *J* = 40.1 Hz), 128.0, 127.9, 125.6, 123.2, 123.0, 116.2, 115.2 (d, *J* = 21.6 Hz), 112.4, 96.2, 86.6, 50.1, 30.6, 21.0 ppm; ^19^F NMR (400 MHz): δ −114.3 ppm; HRMS (ESI-TOF): *m*/*z* [M + H]^+^ calcd. for C_31_H_26_^79^Br FN: 510.1227; found 510.1218. HRMS (ESI-TOF): *m*/*z* [M + H]^+^ calcd. for C_31_H_26_^81^BrFN: 512.1211; found 512.1202.

#### (*Z*)-3-Bromo-4-(3-(4-bromophenyl)-5-phenyl-1-(*p-*tolyl)-pent-2-en-4-yn-1-yl)-*N*-methylaniline (**1c**)

Yellow oil. (1.58 g, 73%), eluent: hexane/ethyl acetate (19:1). ^1^H NMR (400 MHz, CDCl_3_, 298 K): δ 7.71–7.69 (m, 3H), 7.67 (m, 1H), 7.59 (d, *J* = 8.7 Hz, 2H), 7.45–7.43 (m, 3H), 7.30–7.20 (m, 5H), 6.95 (d, *J* = 9.8 Hz, 1H), 6.94 (d, *J* = 2.5 Hz, 1H), 6.62 (dd, *J* = 8.4, 2.5 Hz, 1H), 6.08 (d, *J* = 9.8 Hz, 1H), 2.84 (s, 3H), 2.43 (s, 3H) ppm; ^13^C{^1^H}NMR (100 MHz): δ = 148.9, 140.4, 139.7, 137.0, 135.9 131.5, 130.5, 130.1, 129.3, 128.5, 128.4, 128.0, 125.7, 123.2, 123.1, 116.2, 112.4, 96.4, 86.3, 50.2, 30.6, 21.1 ppm; HRMS (ESI-TOF): *m*/*z* [M + H]^+^ calcd. for C_31_H_26_^79^Br_2_N: 570.0427; found 570.0421. HRMS (ESI-TOF): *m*/*z* [M + H]^+^ calcd. for C_31_H_26_^79^Br^81^BrN: 572.0408; found 572.0429. HRMS (ESI-TOF): *m*/*z* [M + H]^+^ calcd. for C_31_H_26_^81^Br_2_N: 574.0395; found 574.0389.

#### (*Z*)-3-Bromo-*N*-methyl-4-(3-(4-nitrophenyl)-5-phenyl-1-(*p-*tolyl)pent-2-en-4-yn-1-yl)aniline (**1d**)

Yellow oil. (207 mg, 76%), eluent: hexane/ethyl acetate (19:1). ^1^H NMR (400 MHz, CDCl_3_, 298 K): δ 8.25 (d, *J* = 9.0 Hz, 2H), 7.89 (d, *J* = 9.0 Hz, 2H), 7.62–7.60 (m, 2H), 7.42–7.40 (m, 3H), 7.22–7.14 (m, 5H), 7.03 (d, *J* = 9.8 Hz, 1H), 6.92 (d, *J* = 2.4 Hz, 1H), 6.61 (dd, *J* = 8.5, 2.5 Hz, 1H), 6.01 (d, *J* = 9.8 Hz, 1H), 2.84 (s, 3H), 2.37 (s, 3H) ppm; ^13^C{^1^H} NMR (100 MHz): δ 148.7, 147.1, 144.2, 142.7, 139.7, 136.1, 131.7, 130.2, 130.0, 129.3, 128.7, 128.4, 127.9, 126.9, 125.6, 123.7, 122.7, 122.6, 116.3, 112.4, 97.1, 85.4, 50.3, 30.6, 21.0 ppm; HRMS (ESI-TOF): *m*/*z* [M + H]^+^ calcd. for C_31_H_26_^79^BrN_2_O_2_: 537.1172; found 537.1130. HRMS (ESI-TOF): *m*/*z* [M + H]^+^ calcd. for C_31_H_26_^81^BrN_2_O_2_: 539.1157; found 539.1110.

#### (*Z*)-3-Bromo-4-(3,5-diphenyl-1-(*p-*tolyl)pent-2-en-4-yn-1-yl)-*N*-methylaniline (**1e**)

Yellow oil. (1.19 g, 68%), eluent: hexane/ethyl acetate (19:1). ^1^H NMR (400 MHz, CDCl_3_, 298 K): δ 7.78–7.82 (m, 2H), 7.68–7.66 (m, 2H), 7.48–7.39 (m, 6H), 7.28 (d, *J* = 7.8 Hz, 2H), 7.23–7.21 (m, 3H), 6.94 (d, *J* = 9.8 Hz, 1H), 6.94 (d, *J* = 2.5 Hz, 1H), 6.62 (dd, *J* = 8.5, 2.5 Hz, 1H), 6.08 (d, *J* = 9.8 Hz, 1H), 2.85 (s, 3H), 2.42 (s, 3H) ppm; ^13^C{^1^H} NMR (100 MHz): δ 148.8, 140.6, 139.2, 138.0, 135.8, 131.7, 130.8, 130.1, 129.2, 128.4, 128.3, 128.0, 127.8, 126.3, 125.7, 124.1, 123.4, 116.1, 112.4, 96.0, 86.9, 50.1, 30.6, 21.0 ppm; HRMS (ESI-TOF): *m*/*z* [M + H]^+^ calcd. for C_31_H_27_^79^BrN: 492.1321; found 492.1329. HRMS (ESI-TOF): *m*/*z* [M + H]^+^ calcd. for C_31_H_27_^81^BrN: 494.1306; found 494.1316.

#### (*Z*)-3-Bromo-4-(3-(4-methoxyphenyl)-5-phenyl-1-(*p-*tolyl)pent-2-en-4-yn-1-yl)-*N*-methylaniline (**1f**)

Yellow oil. (673 mg, 40%), eluent: hexane/ethyl acetate (19:1). ^1^H NMR (400 MHz, CDCl_3_, 298 K): δ 7.80 (d, *J* = 8.8 Hz, 2H), 7.72–7.69 (m, 2H), 7.47–7.42 (m, 3H), 7.32 (d, *J* = 8.0 Hz, 2H), 7.26–7.23 (m, 3H), 7.03 (d, *J* = 8.8 Hz, 2H), 6.94 (d, *J* = 2.4 Hz, 1H), 6.88 (d, *J* = 9.8 Hz, 1H), 6.62 (dd, *J* = 8.4, 2.4 Hz, 1H), 6.10 (d, *J* = 9.8 Hz, 1H), 3.90 (s,3H), 2.84 (s, 3H), 2.44 (s, 3H) ppm; ^13^C{^1^H} NMR (100 MHz): δ 159.5, 148.8, 140.8, 137.5, 135.7, 131.7, 130.9, 130.6, 130.1, 129.2, 128.3, 128.0, 127.5, 125.7, 123.5, 123.4, 116.1, 113.8, 112.3, 95.9, 87.1, 55.3, 50.1, 30.6, 21.1 ppm; HRMS (ESI-TOF): *m*/*z* [M + H]^+^ calcd. for C_32_H_29_^79^BrNO: 522.1427; found 522.1416. HRMS (ESI-TOF): *m*/*z* [M + H]^+^ calcd. for C_32_H_29_^81^BrNO: 524.1408; found 524.1409.

#### (*Z*)-3-Bromo-*N*-methyl-4-(5-phenyl-1,3-di-*p-*tolylpent-2-en-4-yn-1-yl)aniline (**1g**)

Yellow oil. (1.48g, 73%), eluent: hexane/ethyl acetate (19:1). ^1^H NMR (400 MHz, CDCl_3_, 298 K): δ 7.74 (d, *J* = 8.2 Hz, 2H), 7.69–7.67 (m, 2H), 7.44–7.42 (m, 3H), 7.30–7.28 (m, 4H), 7.24–7.21 (m, 3H), 6.94 (d, *J* = 2.5 Hz, 1H), 6.91 (d, *J* = 9.8 Hz, 1H), 6.62 (dd, *J* = 8.4, 2.5 Hz, 1H), 6.08 (d, *J* = 9.8 Hz, 1H), 2.85 (s, 3H), 2.47 (s, 3H), 2.43 (s, 3H) ppm; ^13^C{^1^H} NMR (100 MHz): δ 148.7, 140.7, 138.4, 137.7, 135.7, 135.2, 131.7, 131.0, 130.1, 129.1, 128.3, 128.3, 128.0, 126.2, 125.7, 123.9, 123.5, 116.1, 112.4, 95.9, 87.0, 50.0, 30.6, 21.2, 21.1 ppm; HRMS (ESI-TOF): *m*/*z* [M + H]^+^ calcd. for C_32_H_29_^79^BrN: 506.1483; found 506.1502. HRMS (ESI-TOF): *m*/*z* [M + H]^+^ calcd. for C_32_H_29_^81^BrN: 508.1462; found 508.1491.

#### (*Z*)-3-Bromo-*N*-methyl-4-(5-phenyl-3-(*m-*tolyl)-1-(*p-*tolyl)-pent-2-en-4-yn-1-yl)aniline (**1h**)

Yellow oil. (1.17g, 78%), eluent: hexane/ethyl acetate (19:1). ^1^H NMR (400 MHz, CDCl_3_, 298 K): δ 7.63–7.58 (m, 4H), 7.41–7.38 (m, 3H), 7.32 (t, *J* = 7.9 Hz, 1H), 7.22 (d, *J* = 8.0 Hz, 2H), 7.19–7.16 (m, 4H), 6.91 (d, *J* = 2.4 Hz, 1H), 6.89 (d, *J* = 9.8 Hz, 1H), 6.60 (dd, *J* = 8.5, 2.5 Hz, 1H), 6.00 (d, *J* = 9.8 Hz, 1H), 2.84 (s, 3H), 2.45 (s, 3H), 2.38 (s, 3H) ppm; ^13^C{^1^H} NMR (100 MHz): δ 148.7, 140.6, 139.1, 137.9, 135.7, 131.7, 130.9, 130.1, 129.1, 128.6, 128.2, 128.2, 127.9, 126.9, 125.7, 124.1, 123.5, 123.5, 116.1, 112.3, 95.8, 86.9, 50.0, 30.6, 21.5, 21.0 ppm; HRMS (ESI-TOF): *m*/*z* [M + H]^+^ calcd. for C_32_H_29_^79^BrN: 506.1478; found 506.1464. HRMS (ESI-TOF): *m*/*z* [M + H]^+^ calcd. for C_32_H_29_^81^BrN: 508.1462; found 506.1447.

#### (*E*)-3-Bromo-*N*-methyl-4-(5-phenyl-3-(*o*-tolyl)-1-(*p-*tolyl)pent-2-en-4-yn-1-yl)aniline (**1i**)

Yellow oil. (1.39 g, 71%), eluent: hexane/ethyl acetate (19:1). ^1^H NMR (400 MHz, CDCl_3_, 298 K): δ 7.54–7.52 (m, 2H), 7.37–7.34 (m, 4H), 7.28–7.23 (m, 5H), 7.17 (d, *J* = 8.1 Hz, 2H), 7.13 (d, *J* = 8.5 Hz, 1H), 6.93 (d, *J* = 2.5 Hz, 1H), 6.60 (dd, *J* = 8.4, 2.5 Hz, 1H), 6.43 (d, *J* = 9.9 Hz, 1H), 5.99 (d, *J* = 9.9 Hz, 1H), 2.85 (s, 3H), 2.53 (s, 3H), 2.38 (s, 3H) ppm; ^13^C{^1^H} NMR (100 MHz): δ 148.6, 143.0, 140.5, 139.4, 136.0, 135.7, 131.5, 131.0, 130.3, 130.0, 129.1, 129.1, 128.2, 128.1, 127.9, 127.6, 125.8, 125.7, 124.4, 123.5, 116.2, 112.4, 95.8, 87.4, 49.6, 30.6, 21.0, 20.3 ppm; HRMS (ESI-TOF): *m*/*z* [M + H]^+^ calcd. for C_32_H_29_^79^BrN: 506.1478; found 506.1482. HRMS (ESI-TOF): *m*/*z* [M + H]^+^ calcd. for C_32_H_29_^81^BrN: 508.1462; found 508.1467.

#### (*Z*)-4-(1,3-Bis(4-chlorophenyl)-5-phenylpent-2-en-4-yn-1-yl)-3-Bromo-*N*-methylaniline (**1j**)

Yellow oil. (1.07 g, 60%), eluent: hexane/ethyl acetate (19:1). ^1^H NMR (400 MHz, CDCl_3_, 298 K): δ 7.67 (d, *J* = 8.7 Hz, 2H), 7.57–7.55 (m, 2H), 7.38–7.36 (m, 5H), 7.30–7.28 (m, 2H), 7.21 (d, *J* = 8.3 Hz, 2H), 7.10 (d, *J* = 8.5 Hz, 1H), 6.91 (d, *J* = 2.5 Hz, 1H), 6.77 (d, *J* = 9.7 Hz, 1H), 6.61 (dd, *J* = 8.4, 2.5 Hz, 1H), 5.94 (d, *J* = 9.7 Hz, 1H), 2.84 (s, 3H) ppm; ^13^C{^1^H} NMR (100 MHz): δ 148.7, 141.8, 138.4, 136.1, 133.8, 132.1, 131.6, 130.1, 130.0, 129.3, 128.5, 128.3, 127.5, 125.6, 123.7, 122.9, 116.4, 112.5, 96.6, 86.0, 49.9, 30.7 ppm; HRMS (ESI-TOF): *m*/*z* [M + H]^+^ calcd. for C_30_H_23_^79^BrCl_2_N: 546.0385; found 546.0385. HRMS (ESI-TOF): *m*/*z* [M + H]^+^ calcd. for C_30_H_23_^81^BrCl_2_N: 548.0364; found 548.0373.

#### (*Z*)-3-Bromo-4-(3-(4-chlorophenyl)-5-phenyl-1-(4-(trifluoromethyl)phenyl)pent-2-en-4-yn-1-yl)-*N*-methylaniline (**1k**)

Yellow oil. (407 mg, 47%), eluent: hexane/ethyl acetate (19:1). ^1^H NMR (400 MHz, CDCl_3_, 298 K): δ 7.70 (d, *J* = 8.6 Hz, 2H), 7.62–7.57 (m, 4H), 7.43 (d, *J* = 8.4 Hz, 2H), 7.40–7.38 (m, 5H), 7.13 (d, *J* = 8.4 Hz, 1H), 6.91 (d, *J* = 2.4 Hz, 1H), 6.82 (d, *J* = 9.6 Hz, 1H), 6.61 (dd, *J* = 8.4, 2.5 Hz, 1H), 6.07 (d, *J* = 9.6 Hz, 1H), 2.85 (s, 3H) ppm; ^13^C{^1^H} NMR (100 MHz): δ 149.0, 147.4, 137.9, 136.1, 133.9, 131.7, 130.0, 129.5, 128.6 (q, *J* = 32.4 Hz), 128.6, 128.5, 128.3, 128.3, 127.6, 125.6, 125.4, 125.3, 124.2 (q, *J* = 272.2 Hz), 124.1, 122.8, 116.2, 112.4, 96.9, 85.9, 77.3, 77.0, 76.7, 50.4, 30.5 ppm; ^19^F NMR (400 MHz): δ - 62.2 ppm; HRMS (ESI-TOF): *m*/*z* [M + H]^+^ calcd. for C_31_H_23_^79^BrClF_3_N: 580.0649; found 580.0665. HRMS (ESI-TOF): *m*/*z* [M + H]^+^ calcd. for C_31_H_23_^81^BrClF_3_N: 582.0630; found 582.0650.

#### (*Z*)-3-Bromo-4-(3-(4-chlorophenyl)-1-(4-methoxyphenyl)-5-phenylpent-2-en-4-yn-1-yl)-*N*-methylaniline (**1l**)

Yellow oil. (1.52 g, 72%), eluent: hexane/ethyl acetate (19:1). ^1^H NMR (400 MHz, CDCl_3_, 298 K): δ 7.69 (d, *J* = 8.6 Hz, 2H), 7.63–7.60 (m, 2H), 7.40–7.37 (m, 5H), 7.23 (d, *J* = 8.6 Hz, 2H), 7.15 (d, *J* = 8.5 Hz, 1H), 6.91–6.89 (m, 3H), 6.85 (d, *J* = 9.8 Hz, 1H), 6.59 (dd, *J* = 8.4, 2.5 Hz, 1H), 5.97 (d, *J* = 9.8 Hz, 1H), 3.82 (s, 3H), 2.83 (s, 3H) ppm; ^13^C{^1^H} NMR (100 MHz): δ 158.1, 148.8, 139.6, 136.4, 135.4, 133.6, 131.7, 130.6, 130.0, 129.0, 128.5, 128.4, 128.3, 127.6, 125.6, 123.1, 122.9, 116.1, 113.9, 112.3, 96.3, 86.3, 55.2, 49.6, 30.5 ppm; HRMS (ESI-TOF): *m*/*z* [M + H]^+^ calcd. for C_31_H_26_^79^BrClNO: 542.0881; found 542.0903. HRMS (ESI-TOF): *m*/*z* [M + H]^+^ calcd. for C_31_H_26_^81^BrClNO: 544.0862; found 544.0891.

#### (*Z*)-3-Bromo-4-(3-(4-chlorophenyl)-5-phenyl-1-(*m-*tolyl)-pent-2-en-4-yn-1-yl)-*N*-methylaniline (**1m**)

Yellow oil. (1.45 g, 70%), eluent: hexane/ethyl acetate (19:1). ^1^H NMR (400 MHz, CDCl_3_, 298 K): δ 7.70 (d, *J* = 8.6 Hz, 2H), 7.62–7.60 (m, 2H), 7.41–7.37 (m, 5H), 7.25 (t, *J* = 7.6 Hz, 1H), 7.15 (d, *J* = 8.4 Hz, 1H), 7.12–7.07 (m, 3H), 6.91 (d, *J* = 2.5 Hz, 1H), 6.86 (d, *J* = 9.8 Hz, 1H), 6.60 (dd, *J* = 8.4, 2.5 Hz, 1H), 6.00 (d, *J* = 9.8 Hz, 1H), 2.84 (s, 3H), 2.37 (s, 3H) ppm; ^13^C{^1^H} NMR (100 MHz): δ = 148.7, 143.2, 139.4, 138.0, 136.4, 133.6, 131.7, 130.6, 130.1, 128.7, 128.4, 128.4, 128.3, 127.6, 127.1, 125.6, 125.0, 123.1, 123.0, 116.2, 112.3, 96.3, 86.3, 50.4, 30.6, 21.5 ppm; HRMS (ESI-TOF): *m*/*z* [M + H]^+^ calcd. for C_31_H_26_^79^BrClN: 526.0932; found 526.0930. HRMS (ESI-TOF): *m*/*z* [M + H]^+^ calcd. for C_31_H_26_^81^BrClN: 528.0913; found 528.0920.

#### (*Z*)-3-Bromo-4-(3-(4-chlorophenyl)-5-phenyl-1-(*o*-tolyl)-pent-2-en-4-yn-1-yl)-*N*-methylaniline (**1n**)

Yellow oil. (929 mg, 72%), eluent: hexane/ethyl acetate (19:1). ^1^H NMR (400 MHz, CDCl_3_, 298 K): δ 7.77 (d, *J* = 8.6 Hz, 2H), 7.69–7.66 (m, 2H), 7.46–7.44 (m, 5H), 7.31–7.28 (m, 4H), 7.14 (d, *J* = 8.4 Hz, 1H), 6.96 (d, *J* = 2.4 Hz, 1H), 6.80 (d, *J* = 9.6 Hz, 1H), 6.59 (dd, *J* = 8.4, 2.4 Hz, 1H), 6.12 (d, *J* = 9.6 Hz, 1H), 2.83 (s, 3H), 2.56 (s, 3H) ppm; ^13^C NMR (100 MHz): δ 148.9, 141.9, 139.3, 137.0, 136.3, 133.7, 131.7, 130.6, 129.9, 128.6, 128.5, 127.6, 127.5, 126.6, 126.1, 125.8, 123.3, 123.2, 116.2, 112.0, 96.7, 86.4, 48.1, 30.6, 19.9 ppm; HRMS (ESI-TOF): *m*/*z* [M + H]^+^ calcd. for C_31_H_26_^79^BrClN: 526.0932; found 526.0910. HRMS (ESI-TOF): *m*/*z* [M + H]^+^ calcd. for C_31_H_26_^81^BrClN: 528.0913; found 528.0889.

#### (*Z*)-3-Bromo-4-(3-(4-chlorophenyl)-5-phenyl-1-(thiophen-2-yl)pent-2-en-4-yn-1-yl)-*N*-methylaniline (**1o**)

Yellow oil. (1.36 g, 67%), eluent: hexane/ethyl acetate (19:1). ^1^H NMR (400 MHz, CDCl_3_, 298 K): δ 7.70 (d, *J* = 8.6 Hz, 2H), 7.64–7.62 (m, 2H), 7.40–7.38 (m, 5H), 7.24–7.22 (m, 2H), 7.01–6.99 (m, 1H), 6.89–6.86 (m, 3H), 6.59 (dd, *J* = 8.5, 2.4 Hz, 1H), 6.20 (d, *J* = 9.5 Hz, 1H), 2.83 (s, 3H) ppm; ^13^C{^1^H} NMR (100 MHz): δ 149.1, 147.7, 138.3, 136.2, 133.8, 131.7, 130.2, 129.8, 128.5, 128.5, 128.3, 127.7, 126.8, 125.1, 124.5, 124.1, 123.3, 123.0, 115.9, 112.4, 96.7, 85.9, 46.2, 30.5; HRMS (ESI-TOF): *m*/*z* [M + H]^+^ calcd. for C_28_H_22_^79^BrClNS: 518.0307; found 518.0339. HRMS (ESI-TOF): *m*/*z* [M + H]^+^ calcd. for C_28_H_22_^81^BrClNS: 520.0319; found 520.0291.

#### (*Z*)-3-Bromo-4-(3-(4-chlorophenyl)-1-(*p-*tolyl)-5-(trimethylsilyl)pent-2-en-4-yn-1-yl)-*N*-methylaniline (**1p**)

Yellow oil. (1.70 g, 54%), eluent: hexane/ethyl acetate (19:1). ^1^H NMR (400 MHz, CDCl_3_, 298 K): δ 7.63 (d, *J* = 8.7 Hz, 2H), 7.36 (d, *J* = 8.7 Hz, 2H), 7.20–7.18 (m, 4H), 7.14 (d, *J* = 8.5 Hz, 1H), 6.89 (d, *J* = 2.5 Hz, 1H), 6.86 (d, *J* = 9.8 Hz, 1H), 6.58 (dd, *J* = 8.4, 2.5 Hz, 1H), 5.97 (d, *J* = 9.8 Hz, 1H), 2.84 (s, 3H), 2.38 (s, 3H), 0.34 (s, 9H); ^13^C{^1^H} NMR (100 MHz): δ 148.8, 140.5, 140.3, 136.1, 135.8, 133.5, 130.3, 130.0, 129.1, 128.4, 127.8, 127.5, 125.7, 123.0, 116.1, 112.1, 101.9, 101.5, 49.9, 30.5, 21.0, 0.0; HRMS (ESI-TOF): *m*/*z* [M + H]^+^ calcd. for C_28_H_30_^79^BrClNSi: 522.1014; found 522.1019. HRMS (ESI-TOF): *m*/*z* [M + H]^+^ calcd. for C_28_H_30_^81^BrClNSi: 524.0995; found 524.1006.

#### (*Z*)-3-Bromo-4-(3-(4-chlorophenyl)-1-(*p-*tolyl)non-2-en-4-yn-1-yl)-*N*-methylaniline (**1q**)

Yellow oil. (1.22 g, 60%), eluent: hexane/ethyl acetate (19:1). ^1^H NMR (400 MHz, CDCl_3_, 298 K): δ 7.61 (d, *J* = 8.7 Hz, 2H), 7.32 (d, *J* = 8.7 Hz, 2H), 7.14 (m, 4H), 7.10 (d, *J* = 8.4 Hz, 1H), 6.88 (d, *J* = 2.5 Hz, 1H), 6.71 (d, *J* = 9.7 Hz, 1H), 6.57 (dd, *J* = 8.4, 2.5 Hz, 1H), 5.83 (d, *J* = 9.7 Hz, 1H), 2.84 (s, 3H), 2.50 (t, *J* = 7.0 Hz, 2H), 2.35 (s, 3H), 1.64 (quintet, *J* = 7.3 Hz, 2H), 1.50 (sextet, *J* = 7.3 Hz, 2H), 0.97 (t, *J* = 7.3 Hz, 3H); ^13^C{^1^H} NMR (100 MHz): δ 148.6, 140.6, 138.1, 137.0, 135.6, 133.2, 130.9, 130.0, 129.0, 128.2, 127.8, 127.5, 125.6, 123.4, 116.1, 112.3, 97.8, 49.8, 30.8, 30.6, 22.0, 20.9, 19.4, 13.6; HRMS (ESI-TOF): *m*/*z* [M + H]^+^ calcd. for C_29_H_30_^79^BrClN: 506.1245; found 506.1238. HRMS (ESI-TOF): *m*/*z* [M + H]^+^ calcd. for C_29_H_30_^81^BrClN: 508.1225; found 508.1224.

#### (*Z*)-3-Bromo-4-(3-(4-chlorophenyl)-1,5-di-*p*-tolylpent-2-en-4-yn-1-yl)-*N*-methylaniline (**1r**)

Yellow oil. (491 mg, 67%). ^1^H NMR (400 MHz, CDCl_3_, 298 K): δ 7.69 (d, *J* = 8.6 Hz, 2H), 7.50 (d, *J* = 8.1 Hz, 2H), 7.37 (d, *J* = 8.6 Hz, 2H), 7.21–7.14 (m, 7H), 6.89 (d, *J* = 2.4 Hz, 1H), 6.83 (d, *J* = 9.8 Hz, 1H), 6.58 (dd, *J* = 8.5, 2.5 Hz, 1H), 5.98 (d, *J* = 9.8 Hz, 1H), 3.73 (s, broad, 1H), 2.83 (s, 3H), 2.42 (s, 3H), 2.38 (s, 3H); ^13^C{^1^H}NMR (100 MHz): δ 148.8, 140.4, 139.1, 138.5, 136.5, 135.8, 133.5, 131.6, 130.6, 130.0, 129.1, 129.0, 128.4, 127.9, 127.6, 125.6, 123.1, 120.1, 116.1, 112.2, 96.5, 85.7, 50.0, 30.5, 21.5, 21.0; HRMS (ESI-TOF): *m*/*z* [M + H]^+^ calcd. for C_32_H_28_^79^BrClN: 540.1088; found 540.1112. HRMS (ESI-TOF): *m*/*z* [M + H]^+^ calcd. for C_32_H_28_^81^BrClN: 542.1069; found 542.1106.

#### (*Z*)-3-Bromo-4-(3-(4-chlorophenyl)-5-(3,5-dichlorophenyl)-1-(*p*-tolyl)pent-2-en-4-yn-1-yl)-*N*-methylaniline (**1s**)

Yellow oil. (420 mg, 61%). ^1^H NMR (400 MHz, CDCl_3_, 298 K): δ 7.68 (d, *J* = 8.6 Hz, 2H), 7.48 (d, *J* = 1.9 Hz, 2H), 7.42 (d, *J* = 8.6 Hz, 2H), 7.39 (t, *J* = 1.9 Hz, 1H), 7.27–7.21 (m, 4H), 7.17 (d, *J* = 8.4 Hz, 1H), 6.96 (d, *J* = 9.5 Hz, 1H), 6.93 (d, *J* = 2.4 Hz, 1H), 6.61 (dd, *J* = 8.5, 2.5 Hz, 1H), 5.95 (d, *J* = 9.5 Hz, 1H), 3.77 (s, broad, 1H), 2.85 (s, 3H), 2.42 (s, 3H); ^13^C NMR (100 MHz): δ 149.0, 141.4, 140.0, 136.1, 135.9, 134.9, 133.9, 130.3, 130.1, 129.8, 129.3, 128.7, 128.6, 127.9, 127.5, 125.9, 125.6, 122.6, 116.0, 112.3, 93.7, 88.7, 50.2, 30.5, 21.1; HRMS (ESI-TOF): *m*/*z* [M + H]^+^ calcd. for C_31_H_24_^79^BrCl_3_N: 594.0152; found 594.0146. HRMS (ESI-TOF): *m*/*z* [M + H]^+^ calcd. for C_31_H_24_^81^BrCl_3_N: 596.0130; found 596.0159.

### General Procedure for Preparation of Benzofluorenes **2**

To a flask was loaded with a solution of **1** (0.4 mmol), Pd(OAc)_2_ (4.5 mg, 0.02 mmol, 5 mol %), DPEphos (0.02 mmol, 6 mol %), and tributylamine (1.2 mmol, 3.0 equiv) in DMF (8 mL) under a N_2_ atmosphere. The resulting mixture was heated in an oil bath at 160 °C for 16 h. The reaction mixture was neutralized with a diluted HCl solution and then extracted with diethyl ether (15 mL × 2). The combined organic layer was washed with water (10 mL × 5) and brine (10 mL), dried over MgSO_4_, and concentrated under reduced pressure. The crude product was purified by chromatography using hexane/ethyl acetate (19:1) as eluent, unless noted.

#### 9-Chloro-*N*-methyl-11-phenyl-5-(*p-*tolyl)-11*H*-benzo[*a*]fluoren-2-amine (**2a**)

Pale-yellow oil. (376 mg, 86%), eluent: hexane/ethyl acetate (19:1). ^1^H NMR (400 MHz, CDCl_3_, 298 K): δ 7.78 (d, *J* = 9.1 Hz, 1H), 7.71 (d, *J* = 8.1 Hz, 1H), 7.64 (s, 1H), 7.53 (d, *J* = 7.9 Hz, 2H), 7.41–7.36 (m, 3H), 7.33–7.28 (m, 4H), 7.23–7.21 (m, 2H), 6.72 (dd, *J* = 9.1, 2.4 Hz, 1H), 6.60 (d, *J* = 2.3 Hz, 1H), 5.22 (s, 1H), 2.65 (s, 3H), 2.55 (s, 3H); ^13^C{^1^H} NMR (100 MHz): δ 151.0, 146.9, 141.3, 141.0, 140.1, 139.8, 138.5, 137.9, 136.8, 132.3, 132.2, 130.0, 128.9, 128.9, 128.2, 127.3, 126.8, 125.5, 125.2, 120.4, 116.9, 115.4, 101.1, 54.2, 30.2, 21.2; HRMS (ESI-TOF): *m*/*z* [M + H]^+^ calcd. for C_31_H_25_ClN: 446.1670; found 446.1672.

#### 9-Fluoro-*N*-methyl-11-phenyl-5-(*p-*tolyl)-11*H*-benzo[*a*]fluoren-2-amine (**2b**)

Pale-yellow oil. (216 mg, 84%), eluent: hexane/ethyl acetate (19:1). ^1^H NMR (400 MHz, CDCl_3_, 298 K): δ 7.81 (d, *J* = 9.1 Hz, 1H), 7.76 (dd, *J* = 8.3, 4.9 Hz, 1H), 7.68 (s, 1H), 7.57 (d, *J* = 8.0 Hz, 2H), 7.43 (d, *J* = 7.8 Hz, 2H), 7.37–7.30 (m, 3H), 7.26–7.24 (m, 2H), 7.15–7.09 (m, 2H), 6.72 (dd, *J* = 9.1, 2.4 Hz, 1H), 6.63 (d, *J* = 2.4 Hz, 1H), 5.25 (s, 1H), 2.66 (s, 3H), 2.58 (s, 3H) ppm; ^13^C{^1^H} NMR (100 MHz): δ 162.5 (d, *J* = 244.6 Hz), 151.5, 151.5, 146.9, 141.3, 139.6, 138.6, 138.1, 137.5, 136.8, 132.3, 130.0, 128.9, 128.9, 128.2, 126.8, 125.2, 120.3 (d, *J* = 8.6 Hz), 116.8, 115.4, 114.1 (d, *J* = 22.9 Hz), 112.4 (d, *J* = 23.2 Hz), 101.0, 54.3, 30.2, 21.3 ppm; ^19^F NMR (400 MHz): δ - 115.3 ppm; HRMS (ESI-TOF): *m*/*z* [M + H]^+^ calcd. for C_31_H_25_FN: 430.1966; found 430.1936.

#### *N*-Methyl-9-nitro-11-phenyl-5-(*p-*tolyl)-11*H*-benzo[*a*]fluoren-2-amine (**2d**)

Brown solid. (106 mg, 55%), eluent: hexane/ethyl acetate (19:1). m.p.: 108–109 °C. ^1^H NMR (400 MHz, CDCl_3_, 298 K): δ 8.28 (dd, *J* = 8.4, 1.7 Hz, 1H), 8.14 (d, *J* = 1.9 Hz, 1H), 7.84 (d, *J* = 8.4 Hz, 1H), 7.77 (d, *J* = 9.1 Hz, 1H), 7.66 (s, 1H), 7.48 (d, *J* = 8.0 Hz, 2H), 7.37 (d, *J* = 7.8 Hz, 2H), 7.34–7.27 (m, 3H), 7.20–7.18 (m, 2H), 6.82 (dd, *J* = 9.1, 2.4 Hz, 1H), 6.67 (d, *J* = 2.4 Hz, 1H), 5.28 (s, 1H), 2.67 (s, 3H), 2.52 (s, 3H) ppm; ^13^C{^1^H} NMR (100 MHz): δ 150.0, 148.0, 146.7, 146.4, 142.6, 141.9, 139.7, 137.9, 137.1, 136.7, 132.0, 129.8, 129.1, 129.0, 128.4, 128.1, 127.2, 126.5, 123.5, 120.2, 119.4, 117.9, 115.8, 102.3, 54.2, 30.5, 21.2 ppm; HRMS (ESI-TOF): *m*/*z* [M + H]^+^ calcd. for C_31_H_25_N_2_O_2_: 457.1911; found 457.1899.

#### *N*-Methyl-11-phenyl-5-(*p-*tolyl)-11*H*-benzo[*a*]fluoren-2-amine (**2e**)

Pale-yellow oil. (181 mg, 79%), eluent: hexane/ethyl acetate (19:1). ^1^H NMR (400 MHz, CDCl_3_, 298 K): δ 7.87 (d, *J* = 7.5 Hz, 1H), 7.80 (d, *J* = 8.9 Hz, 1H), 7.74 (s, 1H), 7.56 (d, *J* = 7.9 Hz, 2H), 7.45–7.40 (m, 4H), 7.34–7.27 (m, 6H), 6.76–6.72 (m, 2H), 5.34 (s, 1H), 2.68 (s, 3H), 2.57 (s, 3H) ppm; ^13^C{^1^H} NMR (100 MHz): δ = 149.4, 146.6, 141.9, 141.5, 141.0, 140.0, 139.0, 138.7, 136.7, 132.3, 130.0, 128.9, 128.7, 128.3, 128.3, 127.1, 126.7, 126.6, 125.5, 124.8, 119.6, 116.7, 115.8, 101.5, 54.3, 30.3, 21.2 ppm; HRMS (ESI-TOF): *m*/*z* [M + H]^+^ calcd. for C_31_H_26_N: 412.2060; found 412.2082.

#### 9-Methoxy-*N*-methyl-11-phenyl-5-(*p-*tolyl)-11*H*-benzo[*a*]fluoren-2-amine (**2f**)

Pale-yellow oil. (215 mg, 76%), eluent: hexane/ethyl acetate (19:1). ^1^H NMR (400 MHz, CDCl_3_, 298 K): δ 7.75 (d, *J* = 9.0 Hz, 1H), 7.72 (dd, *J* = 8.0, 0.5 Hz, 1H), 7.63 (s, 1H), 7.52 (d, *J* = 8.0 Hz, 2H), 7.38 (d, *J* = 7.8 Hz, 2H), 7.34–7.30 (m, 2H), 7.27–7.24 (m, 3H), 6.97–6.94 (m, 2H), 6.70 (dd, *J* = 9.1, 2.4 Hz, 1H), 6.66 (d, *J* = 2.4 Hz, 1H), 5.27 (s, 1H), 3.82 (s, 3H), 2.67 (s, 3H), 2.53 (s, 3H) ppm; ^13^C{^1^H} NMR (100 MHz): δ 159.3, 151.3, 146.4, 142.0, 141.0, 139.1, 138.9, 138.7, 136.6, 134.5, 132.3, 130.0, 128.8, 128.7, 128.3, 128.2, 126.5, 125.0, 120.1, 116.3, 115.5, 112.7, 111.0, 101.3, 55.4, 54.3, 30.4, 21.2 ppm; HRMS (ESI-TOF): *m*/*z* [M + H]^+^ calcd. for C_32_H_28_NO: 442.2165; found 442.2131.

#### 9-Methyl-*N*-methyl-11-phenyl-5-(*p-*tolyl)-11*H*-benzo[*a*]fluoren-2-amine (**2g**)

Pale-yellow oil. (220 mg, 83%), eluent: hexane/ethyl acetate (19:1). ^1^H NMR (400 MHz, CDCl_3_, 298 K): δ 7.80 (d, *J* = 8.8 Hz, 1H), 7.76 (d, *J* = 7.6 Hz, 1H), 7.71 (s, 1H), 7.56 (d, *J* = 7.4 Hz, 2H), 7.41 (d, *J* = 7.4 Hz, 2H), 7.37–7.33 (m, 2H), 7.29–7.22 (m, 5H), 6.76–6.73 (m, 2H), 5.30 (s, 1H), 2.68 (s, 3H), 2.57 (s, 3H), 2.43 (s, 3H) ppm; ^13^C{^1^H} NMR (100 MHz): δ 149.7, 146.2, 142.1, 140.9, 139.7, 139.1, 138.8, 138.7, 136.7, 136.6, 132.3, 130.0, 128.9, 128.7, 128.3, 128.3, 127.9, 126.5, 125.6, 125.5, 119.3, 116.6, 115.8, 101.9, 54.2, 30.5, 21.6, 21.2 ppm; HRMS (ESI-TOF): *m*/*z* [M + H]^+^ calcd. for C_32_H_28_N: 426.2216; found 426.2195.

#### 8-Methyl-*N*-methyl-11-phenyl-5-(*p*-tolyl)-11*H*-benzo[*a*]fluoren-2-amine (**2h**) + 10-methyl-*N*-methyl-11-phenyl-5-(*p*-tolyl)-11*H*-benzo[*a*]fluoren-2-amine (**2h′**)

Pale-yellow oil. (186 mg, **2h**: **2h′** = 42%: 30%, not separable), eluent: hexane/ethyl acetate (19:1). Compound **2h**. ^1^H NMR (400 MHz, CDCl_3_, 298 K): δ 7.88 (d, *J* = 9.8 Hz, 1H), 7.85–7.81 (m, 1H), 7.78 (s, 1H), 7.66–7.62 (m, 2H), 7.50–7.44 (m, 3H), 7.41–7.32 (m, 5H), 7.19 (d, *J* = 8.1 Hz, 1H), 6.76–6.73 (m, 2H), 5.34 (s, 1H), 2.69 (s, 3H), 2.64 (s, 3H), 2.59 (s, 3H) ppm; ^13^C{^1^H} NMR (100 MHz): δ 146.8, 146.8, 142.3, 141.7, 141.0, 140.5, 139.1, 138.9, 136.8, 132.5, 130.1, 129.2, 129.0, 128.8, 128.3, 128.3, 127.7, 126.5, 125.5, 124.6, 120.3, 116.8, 115.8, 101.3, 54.0, 30.3, 21.7, 21.3 ppm. Compound **2h′**. ^1^H NMR (400 MHz, CDCl_3_, 298 K): δ 7.85–7.81 (m, 3H), 7.66–7.62 (m, 2H), 7.50–7.44 (m, 2H), 7.41–7.32 (m, 6H), 7.16 (d, *J* = 7.5 Hz, 1H), 6.93 (d, *J* = 2.2 Hz, 1H), 6.72 (dd, *J* = 9.1, 2.3 Hz, 1H), 5.37 (s, 1H), 2.78 (s, 3H), 2.63 (s, 3H), 2.27 (s, 3H) ppm; ^13^C{^1^H} NMR (100 MHz): δ 147.7, 146.8, 142.1, 141.2, 141.0, 138.9, 138.6, 136.8, 134.8, 132.3, 130.1, 129.0, 128.6, 128.5, 128.3, 128.2, 127.7, 126.3, 125.6, 124.6, 117.3, 116.9, 115.8, 100.8, 54.2, 30.5, 21.3, 19.1 ppm; HRMS (ESI-TOF): *m*/*z* [M + H]^+^ calcd. for C_31_H_25_ClN: 446.1670; found 446.1638.

#### 7-Methyl-*N*-methyl-11-phenyl-5-(*p-*tolyl)-11*H*-benzo[*a*]fluoren-2-amine (**2i**)

Pale-yellow oil. (99.4 mg, 48%), eluent: hexane/ethyl acetate (19:1). ^1^H NMR (400 MHz, CDCl_3_, 298 K): δ 7.91 (s, 1H), 7.77 (d, *J* = 9.0 Hz, 1H), 7.55 (d, *J* = 8.0 Hz, 2H), 7.40 (d, *J* = 7.8 Hz, 2H), 7.33–7.23 (m, 6H), 7.19–7.18 (m, 2H), 6.75 (dd, *J* = 9.0, 2.4 Hz, 1H), 6.72 (d, *J* = 2.3 Hz, 1H), 5.30 (s, 1H), 2.86 (s, 3H), 2.68 (s, 3H), 2.55 (s, 3H) ppm; ^13^C{^1^H} NMR (100 MHz): δ 149.8, 146.4, 142.4, 140.6, 140.4, 140.2, 139.4, 139.0, 136.7, 132.4, 132.0, 130.1, 129.5, 128.9, 128.7, 128.3, 128.0, 126.4, 126.4, 124.9, 122.5, 118.9, 117.0, 101.5, 54.2, 30.4, 21.2, 21.1 ppm; HRMS (ESI-TOF): *m*/*z* [M + H]^+^ calcd. for C_32_H_28_N: 426.2216; found 426.2230.

#### 9-Chloro-5-(4-chlorophenyl)-*N*-methyl-11-phenyl-11*H*-benzo[*a*]fluoren-2-amine (**2j**)

Pale-yellow oil. (165 mg, 89%), eluent: hexane/ethyl acetate (19:1). ^1^H NMR (400 MHz, CDCl_3_, 298 K): δ 7.70–7.65 (m, 2H), 7.58 (s, 1H), 7.53–7.52 (m, 4H), 7.36 (dd, *J* = 8.1, 1.9 Hz, 1H), 7.32–7.27 (m, 4H), 7.20–7.18 (m, 2H), 6.73 (dd, *J* = 9.1, 2.4 Hz, 1H), 6.61 (d, *J* = 2.4 Hz, 1H), 5.20 (s, 1H), 2.65 (s, 3H) ppm; ^13^C{^1^H} NMR (100 MHz): δ 150.9, 146.8, 140.8, 140.3, 139.9, 139.8, 137.9, 133.2, 132.5, 132.2, 131.4, 128.9, 128.4, 128.2, 127.8, 127.4, 126.9, 125.2, 125.2, 120.4, 117.2, 115.4, 101.3, 54.2, 30.2 ppm; HRMS (ESI-TOF): *m*/*z* [M + H]^+^ calcd. for C_30_H_22_Cl_2_N: 466.1124; found 466.1103.

#### 9-Chloro-*N*-methyl-11-phenyl-5-(4-(trifluoromethyl)phenyl)-11*H*-benzo[*a*]fluoren-2-amine (**2k**)

Yellow oil. (114 mg, 88%), eluent: hexane/ethyl acetate (19:1). ^1^H NMR (400 MHz, CDCl_3_, 298 K): δ 7.82 (d, *J* = 8.0 Hz, 2H), 7.71–7.69 (m, 3H), 7.62 (d, *J* = 9.1 Hz, 1H), 7.59 (s, 1H), 7.37 (dd, *J* = 8.1, 1.8 Hz, 1H), 7.34–7.27 (m, 4H), 7.21–7.19 (m, 2H), 6.75 (dd, *J* = 9.1, 2.4 Hz, 1H), 6.62 (d, *J* = 2.4 Hz, 1H), 5.23 (s, 1H), 2.66 (s, 3H) ppm; ^13^C{^1^H} NMR (100 MHz): δ 150.9, 146.9, 145.1, 140.7, 140.6, 139.7, 137.9, 132.6, 132.2, 130.4, 129.4 (q, *J* = 32.8 Hz), 128.9, 128.2, 127.6, 127.4, 126.9, 125.3, 125.1, 124.9, 124.3, 120.4 (q, *J* = 272.6 Hz), 117.3, 115.4, 101.2, 54.2, 30.2 ppm; ^19^F NMR (400 MHz): δ - 62.2 ppm; HRMS (ESI-TOF): *m*/*z* [M + H]^+^ calcd. for C_31_H_22_ClF_3_N: 500.1387; found 500.1364.

#### 9-Chloro-5-(4-methoxyphenyl)-*N*-methyl-11-phenyl-11*H*-benzo[*a*]fluoren-2-amine (**2l**)

Yellow oil. (173 mg, 78%), eluent: hexane/ethyl acetate (19:1). ^1^H NMR (400 MHz, CDCl_3_, 298 K): δ 7.78 (d, *J* = 9.1 Hz, 1H), 7.70 (d, *J* = 8.0 Hz, 1H), 7.62 (s, 1H), 7.54 (d, *J* = 8.7 Hz, 2H), 7.38–7.28 (m, 5H), 7.22–7.20 (m, 2H), 7.12 (d, *J* = 8.7 Hz, 2H), 6.73 (dd, *J* = 9.1, 2.4 Hz, 1H), 6.61 (d, *J* = 2.4 Hz, 1H), 5.21 (s, 1H), 3.96 (s, 3H), 2.65 (s, 3H) ppm; ^13^C{^1^H} NMR (100 MHz): δ 158.9, 151.0, 146.7, 141.0, 140.9, 140.1, 139.7, 137.9, 133.8, 132.3, 132.2, 131.1, 128.9, 128.2, 127.3, 126.8, 125.6, 125.2, 120.4, 116.9, 115.5, 113.7, 101.3, 55.3, 54.1, 30.3 ppm; HRMS (ESI-TOF): *m*/*z* [M + H]^+^ calcd. for C_31_H_25_ClNO: 462.1619; found 462.1632.

#### 9-Chloro-*N*-methyl-11-phenyl-5-(*m-*tolyl)-11*H*-benzo[*a*]fluoren-2-amine (**2m**)

Pale-yellow oil. (136 mg, 88%), eluent: hexane/ethyl acetate (19:1). ^1^H NMR (400 MHz, CDCl_3_, 298 K): δ = 7.80 (d, *J* = 9.1 Hz, 1H), 7.73 (d, *J* = 8.0 Hz, 1H), 7.67 (s, 1H), 7.50–7.45 (m, 3H), 7.39–7.37 (m, 2H), 7.35–7.28 (m, 4H), 7.24–7.22 (m, 2H), 6.75 (dd, *J* = 9.1, 2.4 Hz, 1H), 6.63 (d, *J* = 2.3 Hz, 1H), 5.21 (s, 1H), 2.66 (s, 3H), 2.56 (s, 3H) ppm; ^13^C{^1^H} NMR (100 MHz): δ 151.0, 146.7, 141.5, 141.4, 141.0, 140.1, 139.9, 137.9, 137.8, 132.4, 132.2, 130.8, 128.9, 128.3, 128.2, 128.1, 127.9, 127.4, 127.2, 126.9, 125.5, 125.2, 120.4, 117.0, 115.5, 101.3, 54.2, 30.3, 21.5 ppm; HRMS (ESI-TOF): *m*/*z* [M + H]^+^ calcd. for C_31_H_25_ClN: 446.1670; found 446.1664.

#### 9-Chloro-*N*-methyl-11-phenyl-5-(*o*-tolyl)-11*H*-benzo[*a*]fluoren-2-amine (**2n**)

Pale-yellow oil. (190 mg, 90%, two rotamers 1:1), eluent: hexane/ethyl acetate (19:1). ^1^H NMR (400 MHz, CDCl_3_, 298 K): δ 7.74 (d, *J* = 8.8 Hz, 1H), 7.64 (s, 1H), 7.48–7.45 (m, 4H), 7.42–7.35 (m, 5H), 7.33–7.32 (m, 1H), 7.29–7.27 (m, 2H), 6.72–6.69 (m, 1H), 6.68–6.66 (m, 1H), 5.35 (s, 1H), 2.67 (s, 3H), 2.23 (s, 3H) ppm; ^13^C{^1^H} NMR (100 MHz): δ 151.0, 146.9, 141.0, 140.8, 140.2, 139.9, 138.0, 136.9, 132.4, 132.0, 130.4, 129.9, 128.9, 128.3, 127.6, 127.4, 126.9, 125.8, 125.6, 125.3, 120.5, 117.1, 115.2, 101.1, 54.3, 30.2, 20.2 ppm. The other isomer: ^1^H NMR (400 MHz, CDCl_3_, 298 K): δ 7.73 (dd, *J* = 7.6, 0.8 Hz, 1H), 7.64 (s, 1H), 7.48–7.45 (m, 4H), 7.42–7.35 (m, 5H), 7.33–7.32 (m, 1H), 7.29–7.27 (m, 2H), 6.72–6.69 (m, 1H), 6.68–6.66 (m, 1H), 5.29 (s, 1H), 2.67 (s, 3H), 2.24 (s, 3H) ppm; ^13^C{^1^H} NMR (100 MHz): δ 151.0, 146.9, 141.1, 140.9, 140.2, 139.9, 137.9, 136.9, 132.4, 132.0, 130.4, 129.9, 128.9, 128.3, 127.6, 127.4, 126.9, 125.8, 125.6, 125.3, 120.5, 117.1, 115.2, 101.1, 54.2, 30.2, 20.2 ppm; HRMS (ESI-TOF): *m*/*z* [M + H]^+^ calcd. for C_31_H_25_ClN: 446.1670; found 446.1659.

#### 9-Chloro-*N*-methyl-11-phenyl-5-(thiophen-2-yl)-11*H*-benzo[*a*]fluoren-2-amine (**2o**)

Yellow oil. (177 mg, 80%), eluent: hexane/ethyl acetate (19:1). ^1^H NMR (400 MHz, CDCl_3_, 298 K): δ 8.10 (d, *J* = 9.1 Hz, 1H) 7.80 (s, 1H), 7.71 (d, *J* = 8.0 Hz, 1H), 7.53 (d, *J* = 4.9 Hz, 1H), 7.39–7.37 (m, 2H), 7.33–7.29 (m, 5H), 7.18 (d, *J* = 6.9 Hz, 1H), 6.76 (dd, *J* = 9.0, 1.4 Hz, 1H), 6.55 (d, *J* = 1.4 Hz, 1H), 5.09 (s, 1H), 2.62 (s, 3H) ppm; ^13^C{^1^H} NMR (100 MHz): δ 150.9, 146.9, 142.6, 140.8, 140.7, 139.7, 137.8, 133.2, 132.5, 132.3, 128.9, 128.2, 127.8, 127.4, 127.3, 126.9, 125.5, 125.5, 125.2, 120.5, 117.4, 116.7, 101.1, 54.1, 30.2 ppm; HRMS (ESI-TOF): *m*/*z* [M + H]^+^ calcd. for C_28_H_21_ClNS: 438.1078; found 438.1069.

#### 9-Chloro-*N*-methyl-5-(*p-*tolyl)-11*H*-benzo[*a*]fluoren-2-amine (**2p**)

White solid. (105 mg, 73%), eluent: hexane/ethyl acetate (19:1). m.p.: 225–226 °C. ^1^H NMR (400 MHz, CDCl_3_, 298 K): δ 7.76 (d, *J* = 9.1 Hz, 1H), 7.68 (d, *J* = 8.1 Hz, 1H), 7.61 (d, *J* = 1.3 Hz, 1H), 7.56 (s, 1H), 7.46 (d, *J* = 8.0 Hz, 2H), 7.38 (dd, *J* = 8.1, 1.9 Hz, 1H), 7.35 (d, *J* = 7.8 Hz, 2H), 6.90 (d, *J* = 2.4 Hz, 1H), 6.82 (dd, *J* = 9.1, 2.4 Hz, 1H), 4.13 (s, 2H), 4.00 (s, broad, 1H), 3.02 (s, 3H), 2.50 (s, 3H) ppm; ^13^C{^1^H} NMR (100 MHz): δ 147.3, 145.1, 141.5, 140.4, 138.5, 137.6, 136.7, 132.5, 131.7, 129.9, 128.8, 128.3, 126.9, 125.0, 124.9, 120.3, 117.1, 115.6, 100.0, 35.7, 30.6, 21.2 ppm; HRMS (ESI-TOF): *m*/*z* [M + H]^+^ calcd. for C_25_H_21_ClN: 370.1357; found 370.1351.

#### 9-Chloro-*N*-methyl-5,11-di-*p*-tolyl-11*H*-benzo[*a*]fluoren-2-amine (**2r**)

Yellow oil. (148 mg, 81%). ^1^H NMR (400 MHz, CDCl_3_, 298 K): δ 7.81 (d, *J* = 9.1 Hz, 1H), 7.72 (d, *J* = 8.0 Hz, 1H), 7.66 (s, 1H), 7.56 (d, *J* = 7.9 Hz, 2H), 7.42 (d, *J* = 7.8 Hz, 2H), 7.39–7.36 (m, 2H), 7.17–7.11 (m, 4H), 6.74 (dd, *J* = 9.1, 2.3 Hz, 1H), 6.66 (d, *J* = 2.2 Hz, 1H), 5.20 (s, 1H), 3.79 (s, broad, 1H), 2.69 (s, 3H), 2.57 (s, 3H), 2.39 (s, 3H); ^13^C NMR (100 MHz): δ 151.1, 146.8, 141.1, 140.0, 139.9, 138.5, 137.8, 137.8, 136.8, 136.3, 132.3, 132.2, 129.9, 129.5, 128.9, 128.2, 128.0, 127.2, 125.4, 125.2, 120.3, 116.8, 115.4, 101.2, 53.8, 30.2, 21.2, 21.0; HRMS (ESI-TOF): *m*/*z* [M + H]^+^ calcd. for C_32_H_27_ClN: 460.1827; found 460.1844.

#### 9-Chloro-11-(3,5-dichlorophenyl)-*N*-methyl-5-(*p*-tolyl)-11*H*-benzo[*a*]fluoren-2-amine (**2s**)

Yellow oil. (150 mg, 73%). ^1^H NMR (400 MHz, CDCl_3_, 298 K): δ 7.75 (d, *J* = 9.1 Hz, 1H), 7.69 (d, *J* = 8.0 Hz, 1H), 7.58 (s, 1H), 7.48 (d, *J* = 7.9 Hz, 2H), 7.39–7.36 (m, 3H), 7.28–7.27 (m, 2H), 7.07 (d, *J* = 1.6 Hz, 2H), 6.75 (dd, *J* = 9.1, 2.3 Hz, 1H), 6.51 (d, *J* = 2.2 Hz, 1H), 5.12 (s, 1H), 3.91 (s, broad, 1H), 2.75 (s, 3H), 2.51 (s, 3H); ^13^C NMR (100 MHz): δ 149.4, 147.1, 144.8, 141.8, 140.1, 138.2, 138.2, 137.9, 137.0, 135.2, 132.5, 132.0, 129.9, 128.9, 128.5, 127.9, 127.2, 126.6, 125.5, 125.1, 120.6, 117.0, 115.3, 100.3, 53.1, 30.2, 21.2; HRMS (ESI-TOF): *m*/*z* [M + H]^+^ calcd. for C_31_H_23_Cl_3_N: 514.0896; found 514.0906.

#### 7-(4-Chlorophenyl)-*N*-methyl-8-(pent-1-en-1-yl)-5-(*p-*tolyl)naphthalen-2-amine (**3**)

Pale-yellow oil. (174 mg, 83%; *E*:*Z* = 3.4:1, not separable). Eluent: hexane/ethyl acetate (49:1). *E***-isomer**: ^1^H NMR (400 MHz, CDCl_3_, 298 K): δ 7.79 (d, *J* = 9.0 Hz, 1H), 7.47–7.41 (m, 3H), 7.40–7.32 (m, 5H), 7.30–7.28 (m, 2H), 7.12 (s, 1H), 6.87 (dd, *J* = 9.1, 2.4 Hz, 1H), 6.57 (dt, *J* = 16.0, 1.4 Hz, 1H), 5.73 (dt, *J* = 16.1, 7.0 Hz, 1H), 2.98 (s, 3H), 2.47 (s, 3H), 2.19 (qd, *J* = 7.2, 1.3 Hz, 2H), 1.45 (sextet, *J* = 7.3, 2H), 0.95 (t, *J* = 7.3, 3H) ppm; ^13^C{^1^H} NMR (100 MHz): δ 147.1, 141.5, 139.0, 138.0, 136.7, 136.6, 134.3, 132.2, 131.7, 131.3, 131.3, 129.9, 128.9, 127.8, 127.4, 126.9, 125.3, 125.0, 117.3, 102.8, 35.6, 30.7, 22.4, 21.2, 13.7 ppm. *Z***-isomer:**^1^H NMR (400 MHz, CDCl_3_, 298 K): δ 7.80 (d, *J* = 9.0 Hz, 1H), 7.47–7.41 (m, 3H), 7.40–7.32 (m, 5H), 7.30–7.28 (m, 2H), 7.12 (s, 1H), 6.87 (dd, *J* = 9.1, 2.3 Hz, 1H), 6.60 (dt, *J* = 11.2, 1.2 Hz, 1H), 5.73 (dt, *J* = 11.7, 7.1 Hz, 1H), 2.97 (s, 3H), 2.48 (s, 3H), 1.71–1.69 (m, 2H), 1.30 (m, 2H), 0.77 (t, *J* = 7.4, 3H) ppm; ^13^C{^1^H} NMR (100 MHz): δ 147.1, 141.4, 139.4, 138.0, 137.1, 136.7, 134.2, 133.6, 132.4, 131.7, 130.8, 130.2, 128.9, 128.0, 127.3, 126.6, 124.9, 124.7, 117.3, 103.0, 35.6, 31.3, 22.0, 21.2, 13.9 ppm; HRMS (ESI-TOF): *m*/*z* [M + H]^+^ calcd. for C_29_H_29_ClN: 426.1983; found 426.1974.

#### 9-Chloro-5-methoxy-11*H*-benzo[*a*]fluorene (**5**)

White solid. (96.1 mg, 51%); eluent: hexane/ethyl acetate (99:1). m.p.: 135–136 °C. ^1^H NMR (400 MHz, CDCl_3_, 298 K): δ 8.33 (d, *J* = 8.3 Hz, 1H), 7.63 (d, *J* = 8.1 Hz, 1H), 7.59–7.47 (m, 3H), 7.38 (d, *J* = 8.0 Hz, 2H), 7.11 (s, 1H), 4.09 (s, 3H), 3.95 (s, 2H) ppm; ^13^C{^1^H} NMR (100 MHz): δ 155.8, 145.4, 141.4, 137.7, 131.7, 131.5, 131.0, 127.0, 126.8, 125.2, 125.0, 124.8, 123.7, 123.2, 119.9, 96.5, 55.6, 34.9 ppm; HRMS (ESI-TOF): *m*/*z* [M + H]^+^ calcd. for C_18_H_14_ClO: 281.0728; found 281.0710.

#### (*Z*)-(5-(2-Bromophenyl)-3-(4-chlorophenyl)-5-methoxypent-3-en-1-yn-1-yl)trimethylsilane (**4**)

To a stirring solution of (*E*)-1-(2-bromophenyl)-3-(4-chlorophenyl)-5-(trimethylsilyl)-pent-1-en-4-yn-3-ol (4 mmol) in MeOH (40 mL) was added sulfuric acid (0.6 mmol, 15 mol %). The reaction was kept at room temperature for 16 h. The solution was neutralized with a saturated aqueous solution of NaHCO_3_. The mixture was concentrated under reduced pressure, and then the residue was extracted with EtOAc (30 mL × 2). The combined extracts were washed with water (20 mL × 2) and brine (20 mL), dried over MgSO_4_, and concentrated under reduced pressure to afford **4** (868 mg, 100%) as pale-yellow oil. This compound was used for the next step without further purification. ^1^H NMR (400 MHz, CDCl_3_, 298 K): δ 7.66–7.61 (m, 4H), 7.41 (t, *J* = 7.5 Hz, 1H), 7.35 (d, *J* = 8.6 Hz, 2H), 7.20 (td, *J* = 7.6, 1.6 Hz, 1H), 6.41 (d, *J* = 9.0 Hz, 1H), 5.84 (d, *J* = 9.0 Hz, 1H), 3.53 (s, 3H), 0.38 (s, 9H) ppm; ^13^C{^1^H} NMR (100 MHz): δ 140.1, 135.7, 135.2, 134.2, 132.9, 129.1, 128.5, 128.4, 127.7, 127.6, 126.3, 123.4, 102.8, 101.1, 80.3, 56.6, −0.1 ppm; HRMS (ESI-TOF): *m*/*z* [M + H]^+^ calcd. for C_21_H_23_^79^BrClOSi: 433.0390; found 433.0399. HRMS (ESI-TOF): *m*/*z* [M + H]^+^ calcd. for C_21_H_23_^81^BrClOSi: 435.0370; found 435.0381.

### Gram-Scale Test

To a flask were loaded **1a** (2.0 mmol, 1.05 g), Pd(OAc)_2_ (22.5 mg, 0.10 mmol, 5 mol %), DPEphos (0.12 mmol, 6 mol %), and tributylamine (6.0 mmol, 3.0 equiv) in DMF (40 mL) under a N_2_ atmosphere. The mixture was heated in an oil bath at 160 °C for 16 h. The reaction mixture was neutralized with a diluted HCl solution and then extracted with diethyl ether (40 mL × 2). The combined organic layer was washed with water (30 mL × 5) and brine (30 mL), dried over MgSO_4_, and concentrated under reduced pressure. The crude product was purified by chromatography using hexane/ethyl acetate (19:1) as eluent to give **2a** (758 mg, 85%).

### Crystallography

Crystal of **2d** for X-ray determination was obtained by recrystallization from diethyl ether/hexane solutions. The structure was solved using the SHELXS-97 program^10^ and refined using the SHELXL-97 program^11^ by full-matrix least squares on F2 values.

## Data Availability

The data underlying this study are available in the published article and its online Supporting Information.

## Supporting Information Available

ORTEP plot (Figure S1) and crystal data of **2d** (Table S1) and spectra of all compounds (PDF)
