# Supplementary material for: Preparation of Benzo[a]fluorenes via Pd-Catalyzed Annulation of 5-(2-Bromophenyl)pent-3-en-1-ynes
Source: J Org Chem. 2024 Aug 9;89(17):12341–8. doi: 10.1021/acs.joc.4c01286 (PMC11382150; doi:10.1021/acs.joc.4c01286)

# Preparation of Benzo[*a*]fluorenes via Pd-Catalyzed Annulation of 5-(2-bromophenyl)pent-3-en-1-ynes

Cheng-Kai Hsu, Yi-Hung Liu and Shiuh-Tzung Liu*

## Supplementary Material

Figure S1 ORTEP plot of **2d** (30% probability ellipsoids) …………………..……S1
Table S1. Crystal data for **2d**……………………………………………………S2
Spectra for all compounds………………………………………………………S3-S41

Figure S1 ORTEP plot of **2d** (30% probability ellipsoids)

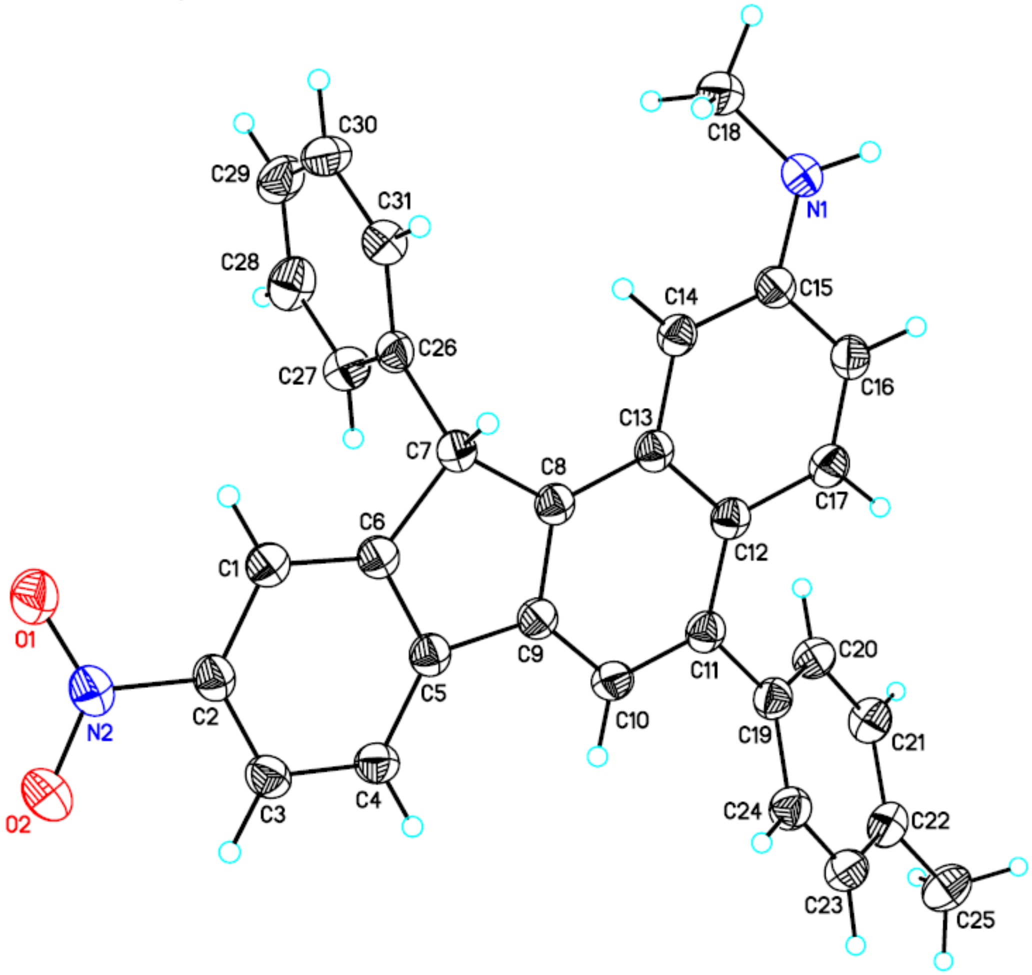

Table S1. Crystal data for **2d** (ic22384).

| | | |
|---|---|---|
| Crystal data | | |
| Empirical formula | C31 H24 N2 O2 | |
| Formula weight | 456.52 | |
| Crystal system | Monoclinic | |
| Space group | $P2_1/n$ | |
| Unit cell dimensions | a = 10.8571(3) Å | α= 90°. |
| | b = 14.4354(4) Å | β= 107.6044(12)°. |
| | c = 15.3784(4) Å | γ = 90°. |
| Volume | 2297.33(11) Å $^3$ | |
| Z | 4 | |
| F(000) | 960 | |
| Density (calculated) | 1.320 Mg/m$^3$ | |
| Wavelength | 1.54178 Å | |
| Cell parameters reflections used | 9686 | |
| Theta range for Cell parameters | 4.30 to 68.25°. | |
| Absorption coefficient | 0.654 mm$^{-1}$ | |
| Temperature | 100(2) K | |
| Crystal size | 0.150 x 0.150 x 0.100 mm$^3$ | |
| Data collection | | |
| Diffractometer | Bruker AXS D8 VENTURE, PhotonIII_C28 | |
| Absorption correction | Semi-empirical from equivalents | |
| Max. and min. transmission | 1.0000 and 0.8238 | |
| No. of measured reflections | 39183 | |
| No. of independent reflections | 4189 [R(int) = 0.0653] | |
| No. of observed [I>2_igma(I)] | 3604 | |
| Completeness to theta = 67.679° | 99.6 % | |
| Theta range for data collection | 4.298 to 68.528°. | |
| Refinement | | |
| Final R indices [I>2sigma(I)] | R1 = 0.0467, wR2 = 0.1242 | |
| R indices (all data) | R1 = 0.0541, wR2 = 0.1332 | |
| Goodness-of-fit on F$^2$ | 1.013 | |
| No. of reflections | 4189 | |
| No. of parameters | 318 | |
| No. of restraints | 0 | |
| Largest diff. peak and hole | 0.404 and -0.413 e.Å $^{-3}$ | |

$^{1}H$ NMR (400 MHz, $CDCl_3$) Spectrum of **1a**

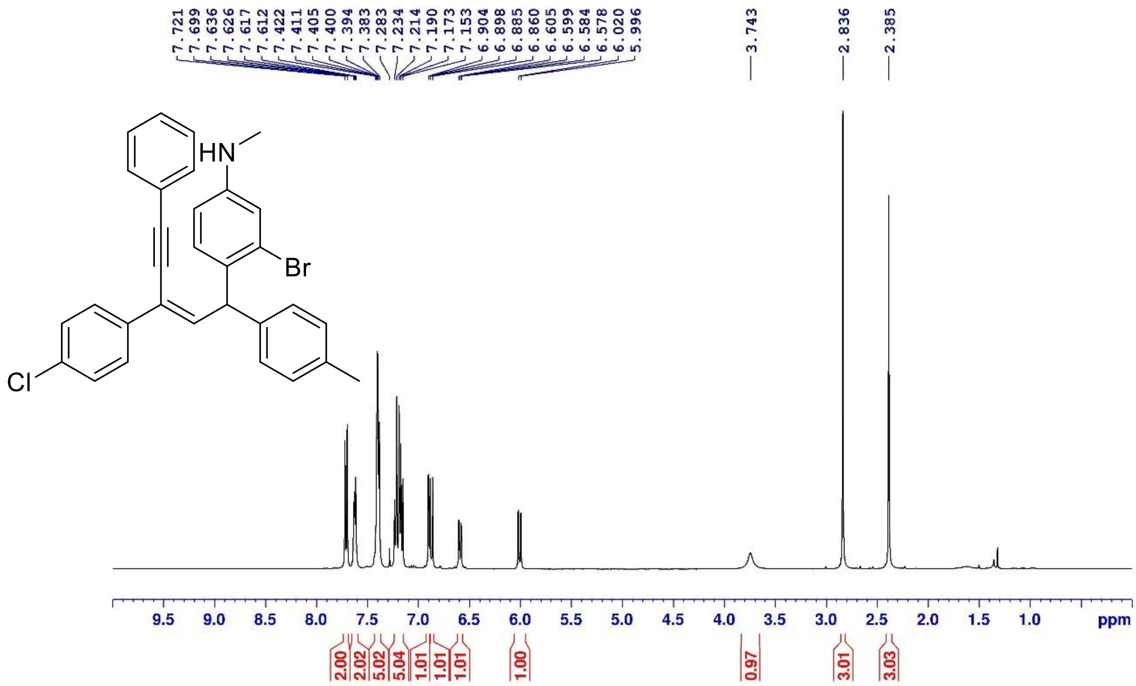

$^{13}C\{^{1}H\}$ NMR (100 MHz, $CDCl_3$) **1a**

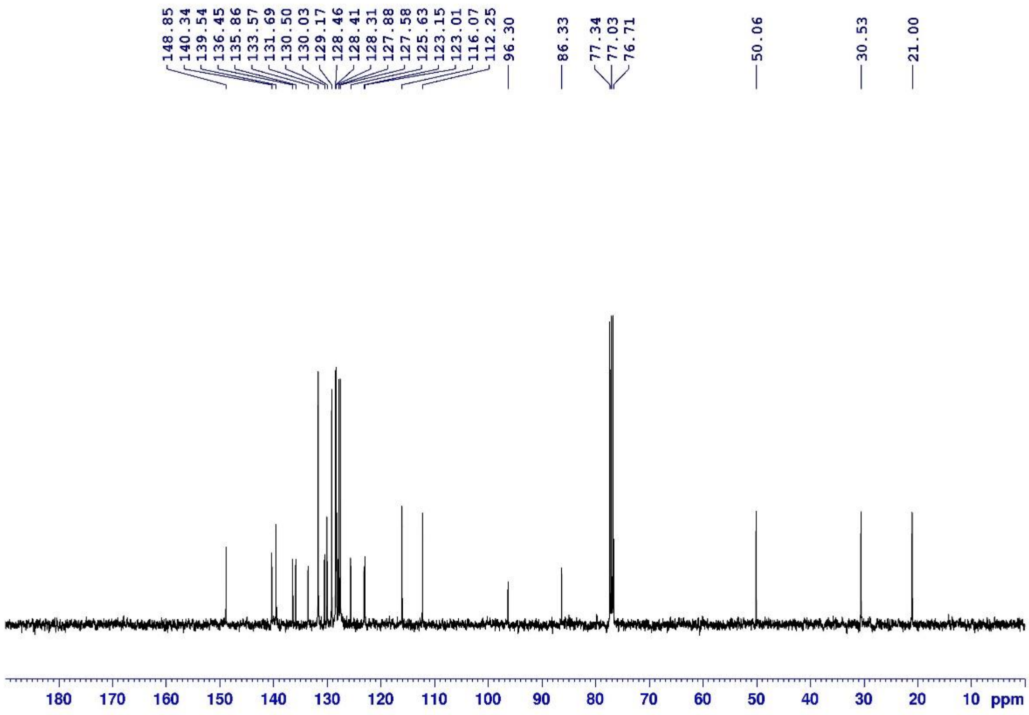

$^{1}$H NMR (400 MHz, $CDCl_3$) Spectrum of **1b**

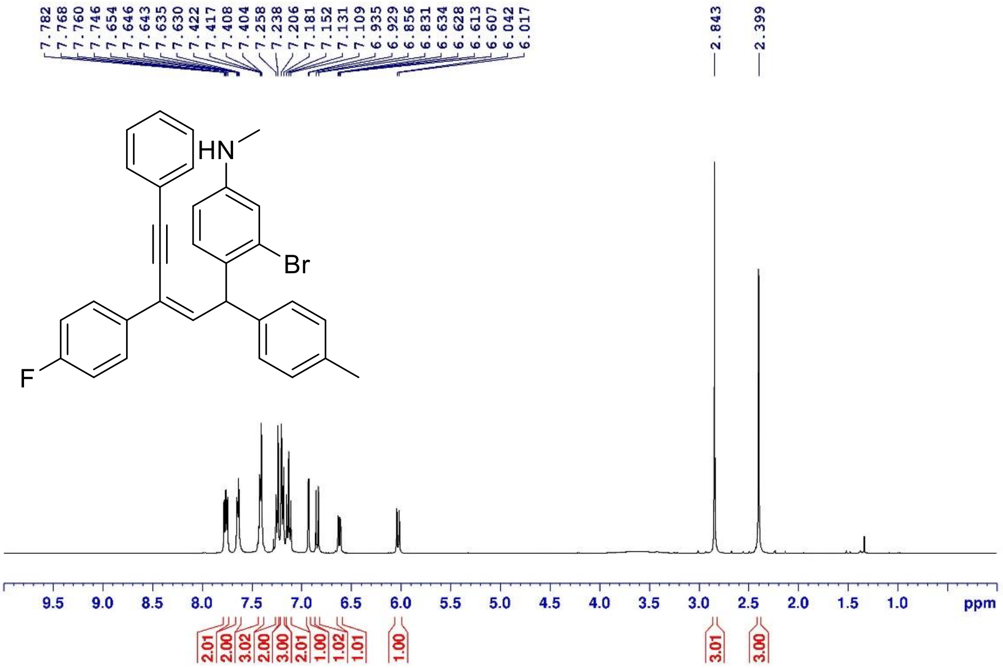


$^{13}$C{$^{1}$H} NMR (100 MHz, $CDCl_3$) **1b**

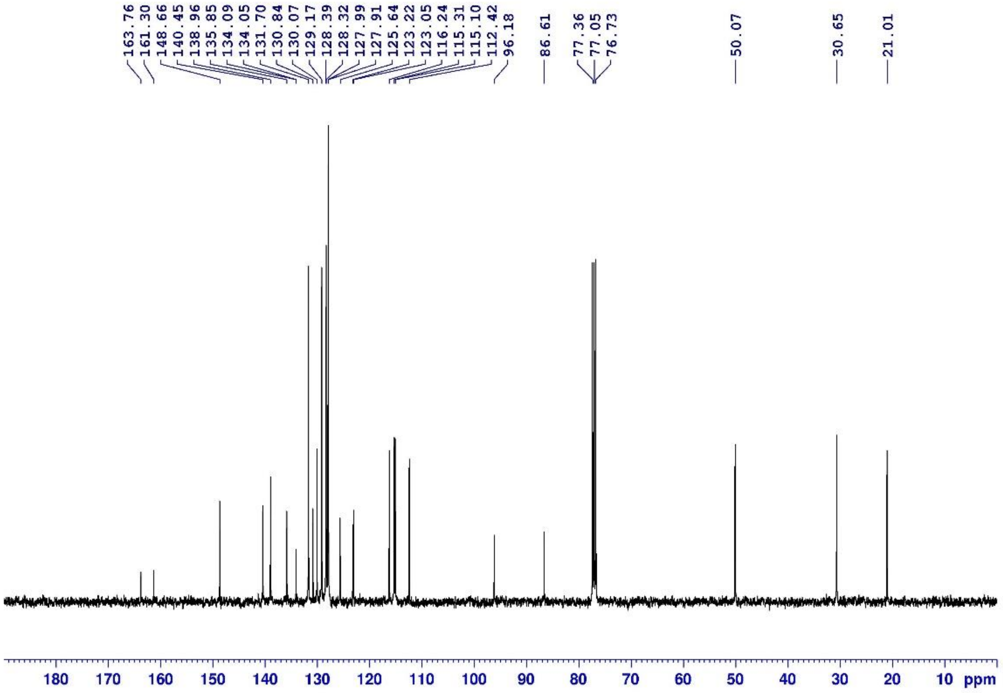

$^1$H NMR (400 MHz, $CDCl_3$) Spectrum of **1c**

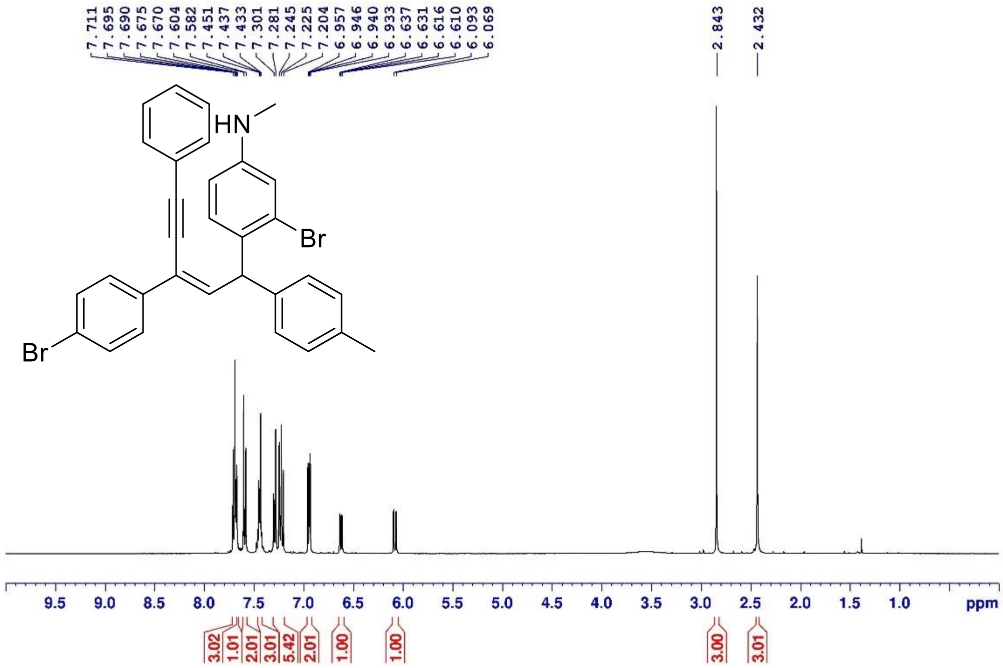

$^{13}C\{^1H\}$ NMR (100 MHz, $CDCl_3$) **1c**

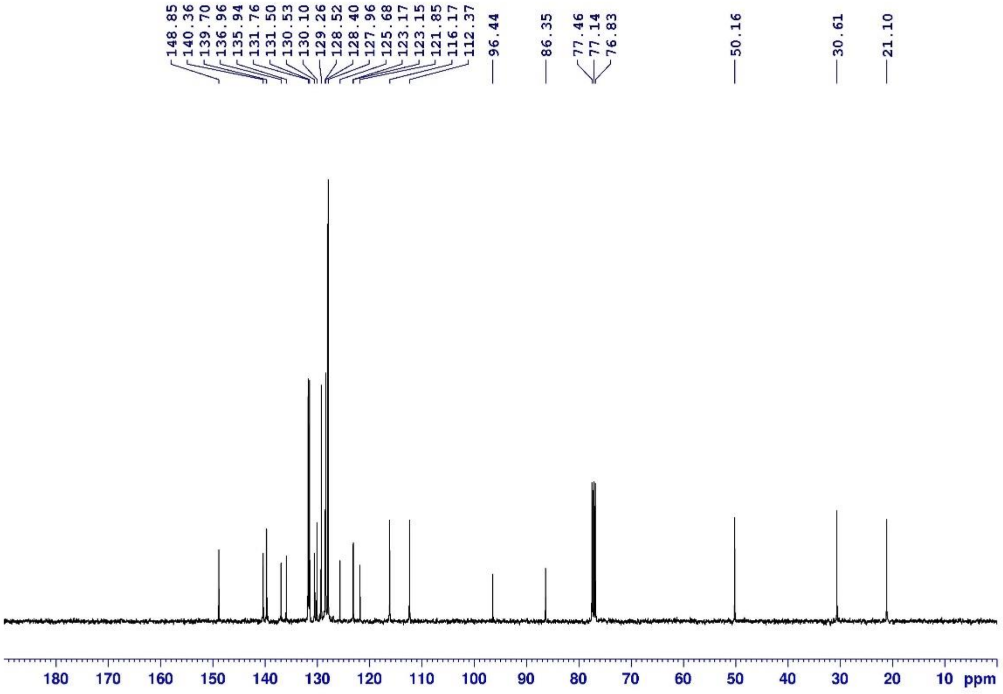

$^{1}H$ NMR (400 MHz, $CDCl_3$) Spectrum of **1d**

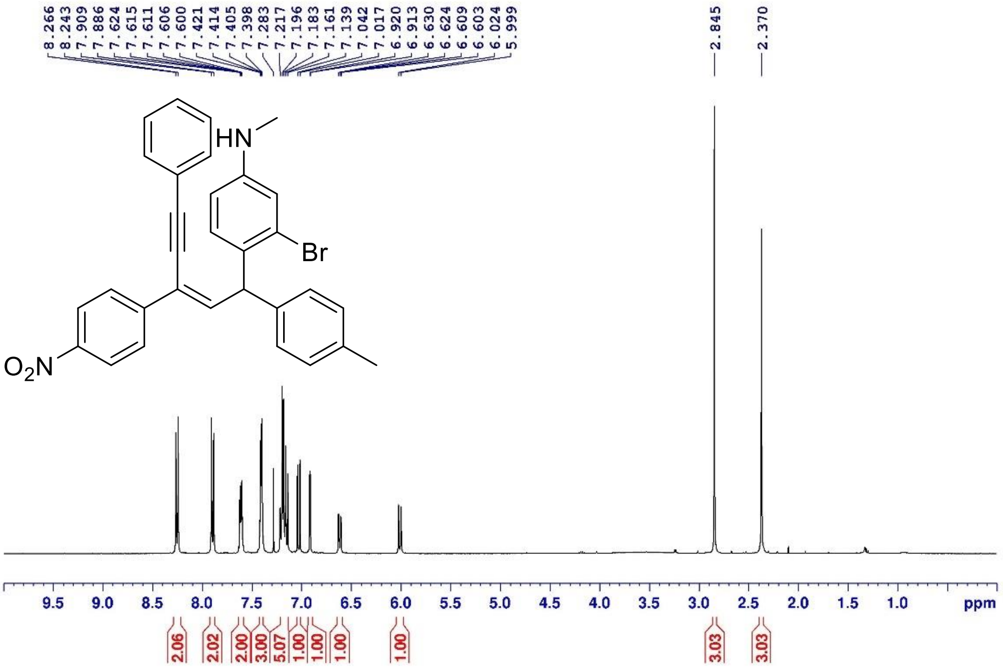


$^{13}C\{^{1}H\}$ NMR (100 MHz, $CDCl_3$) **1d**

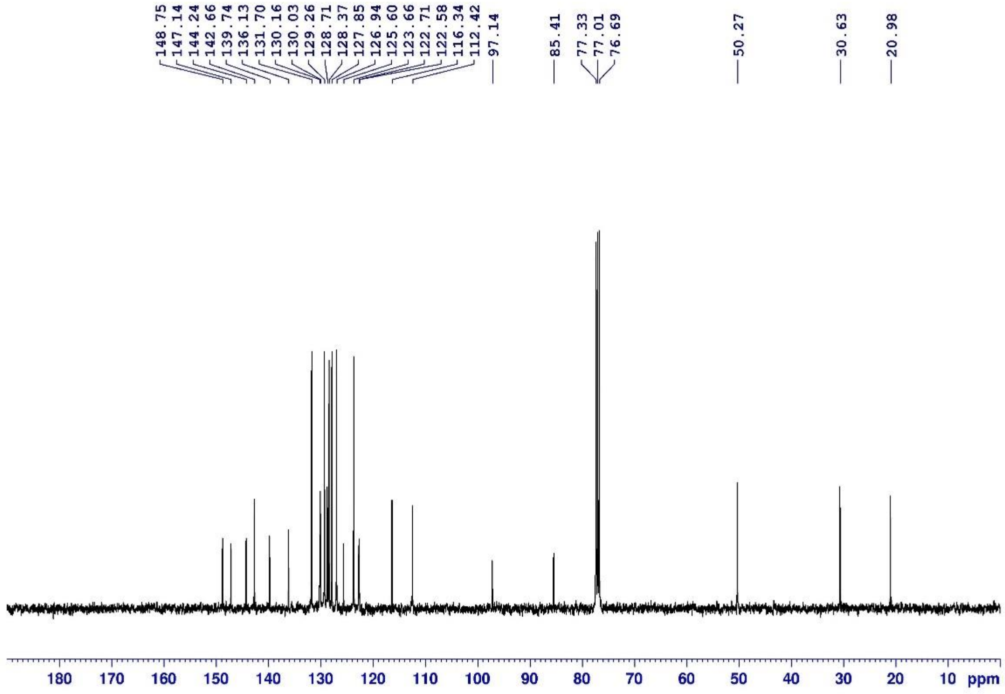

$^{1}$H NMR (400 MHz, $CDCl_3$) Spectrum of **1e**

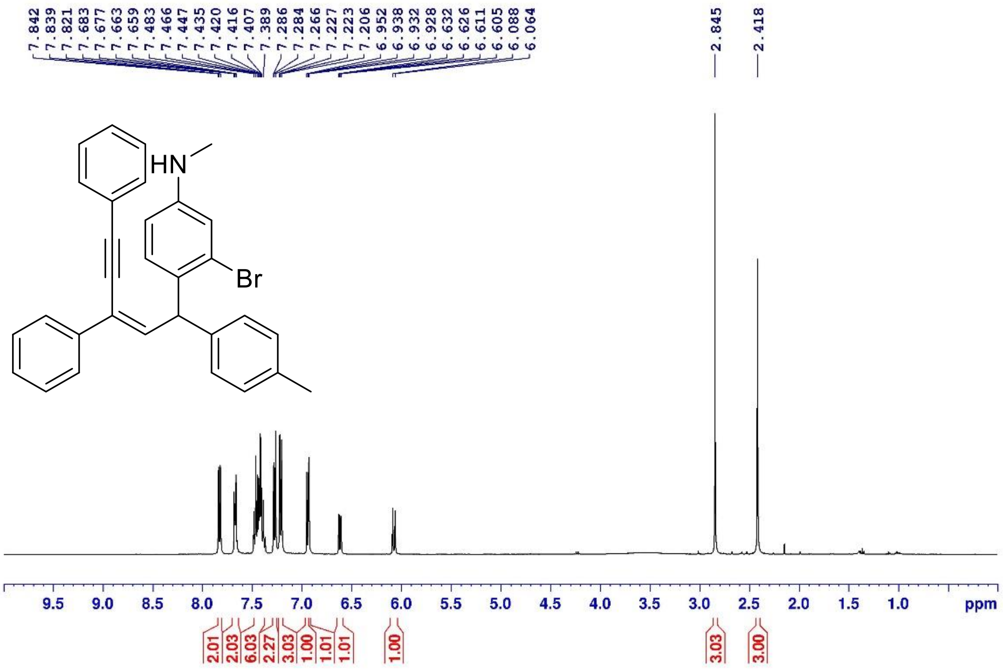


$^{13}$C{$^{1}$H} NMR (100 MHz, $CDCl_3$) **1e**

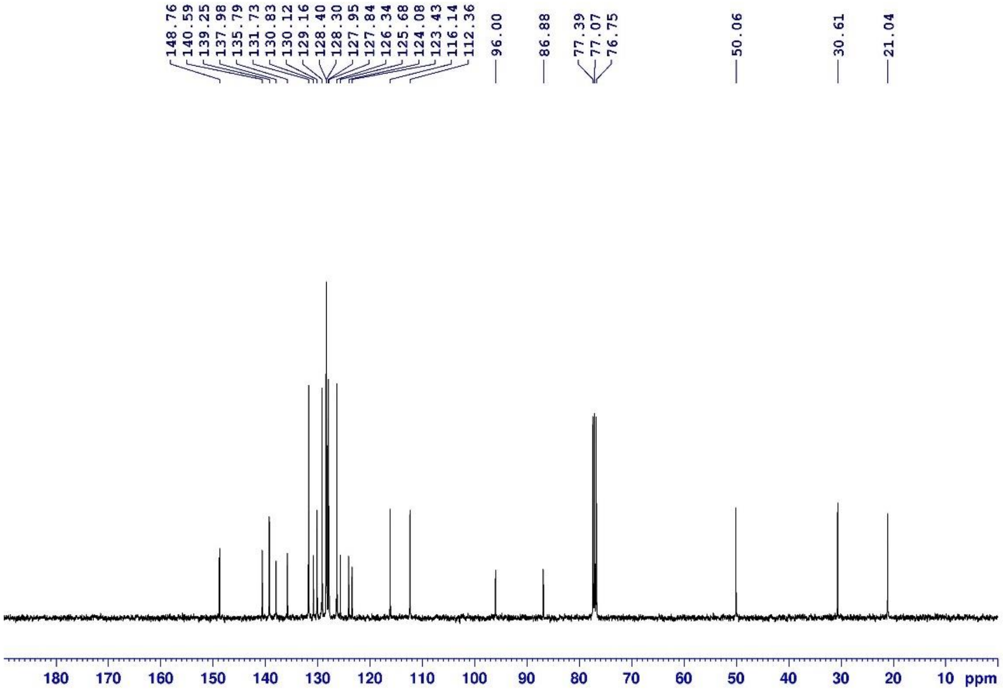

$^1$H NMR (400 MHz, $CDCl_3$) Spectrum of **1f**

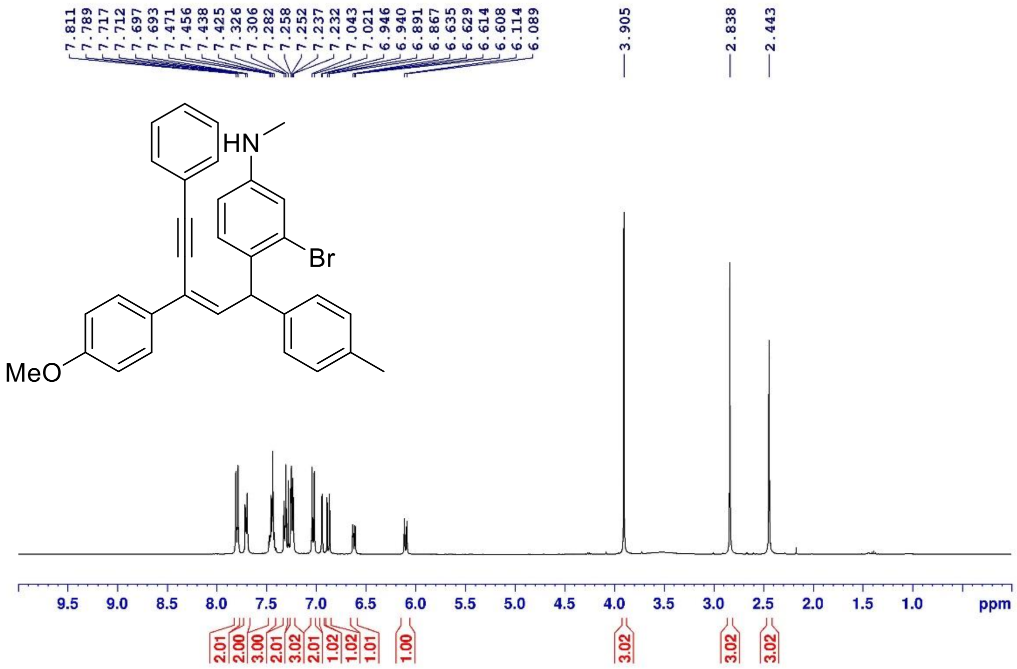

$^{13}C\{^1H\}$ NMR (100 MHz, $CDCl_3$) **1f**

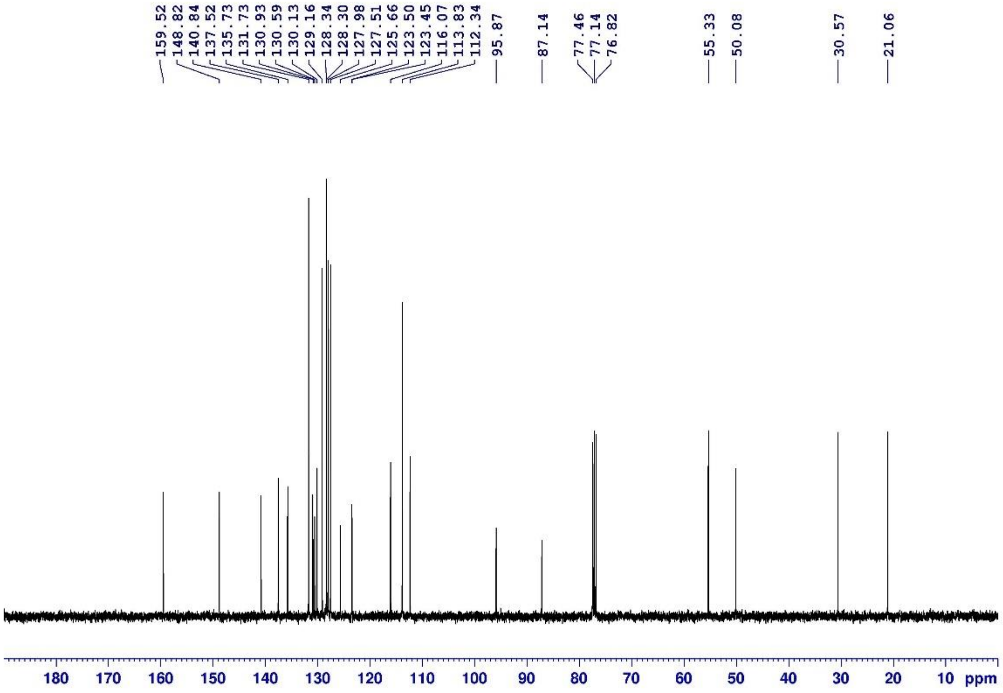

$^{1}H$ NMR (400 MHz, $CDCl_3$) Spectrum of **1g**

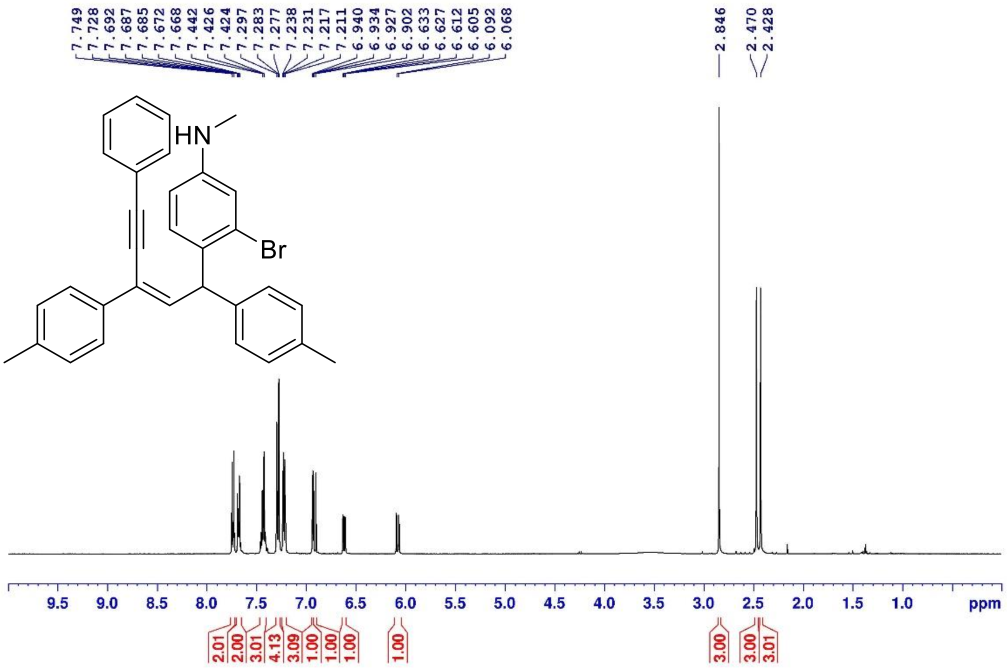


$^{13}C\{^{1}H\}$ NMR (100 MHz, $CDCl_3$) **1g**

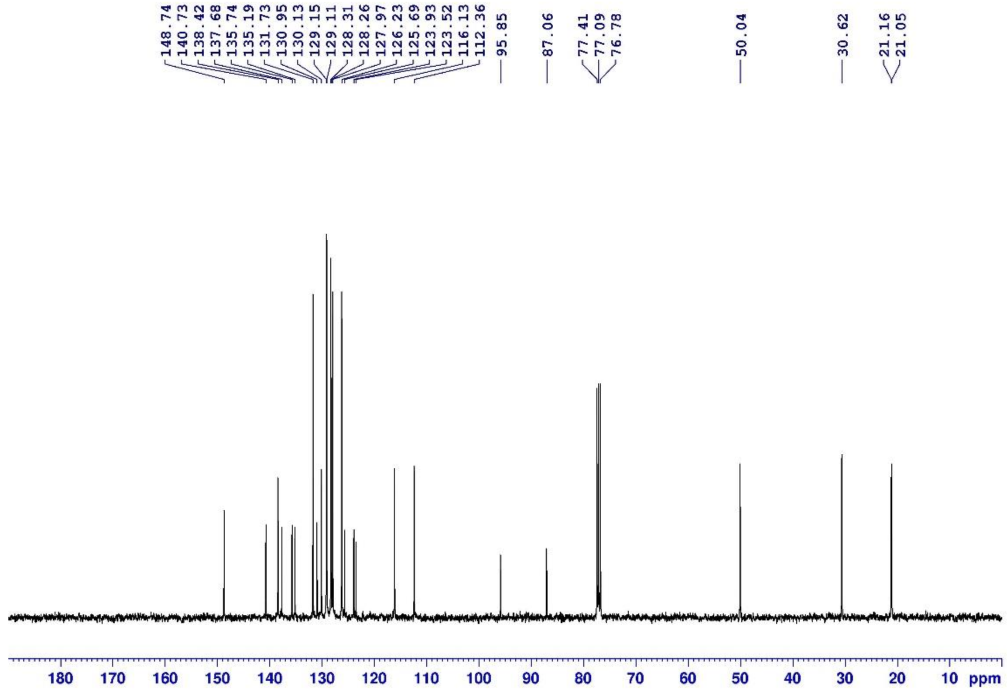

**$^{1}$H NMR (400 MHz, $CDCl_3$) Spectrum of 1h**

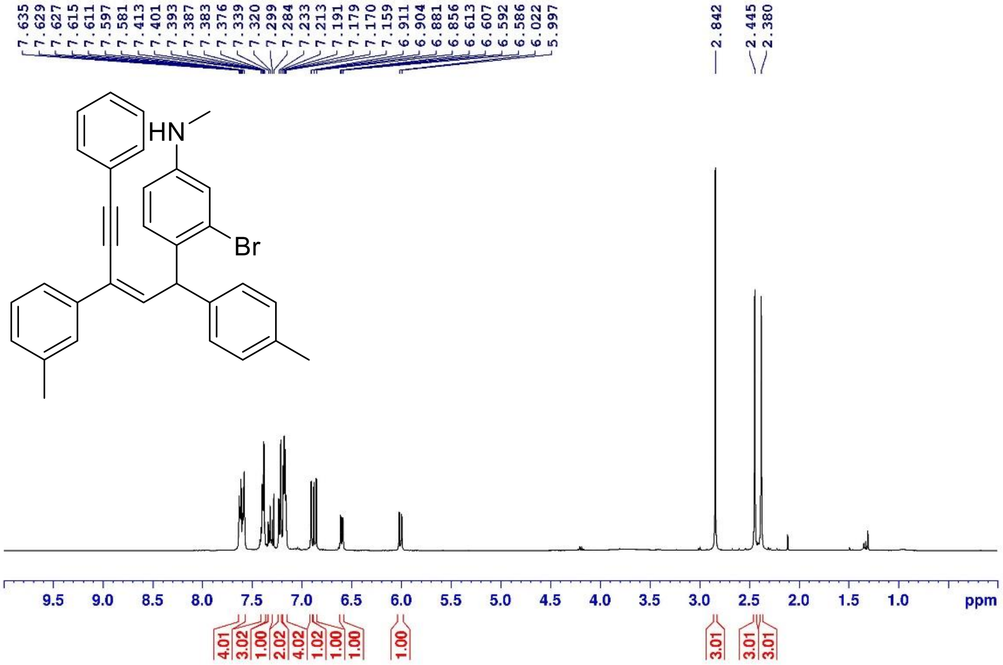


**$^{13}$C{$^{1}$H} NMR (100 MHz, $CDCl_3$) 1h**

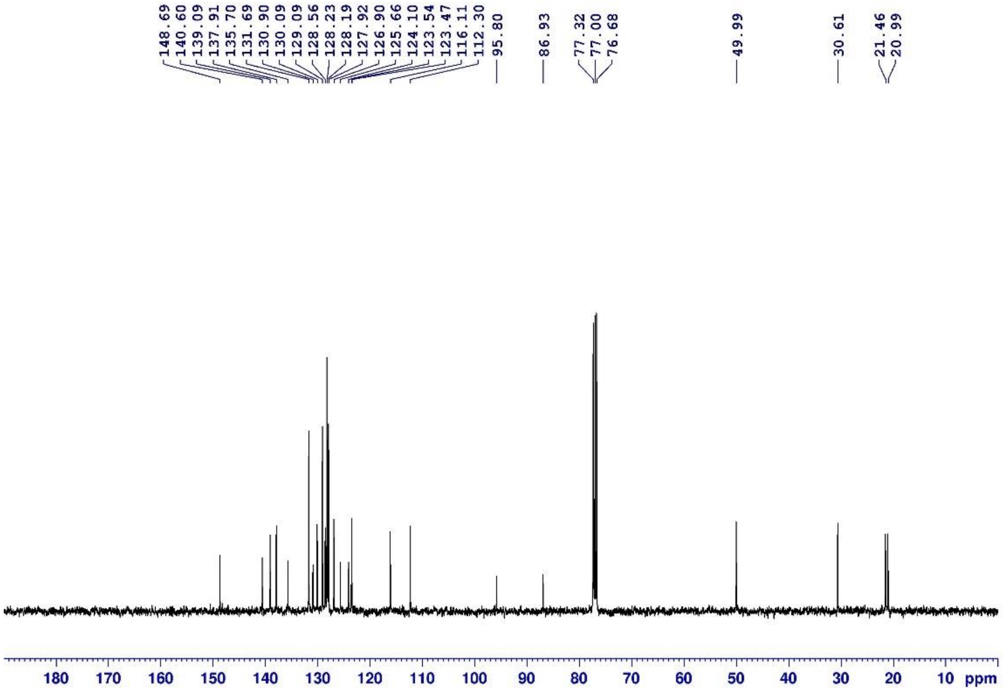

$^{1}H$ NMR (400 MHz, $CDCl_3$) Spectrum of **1i**

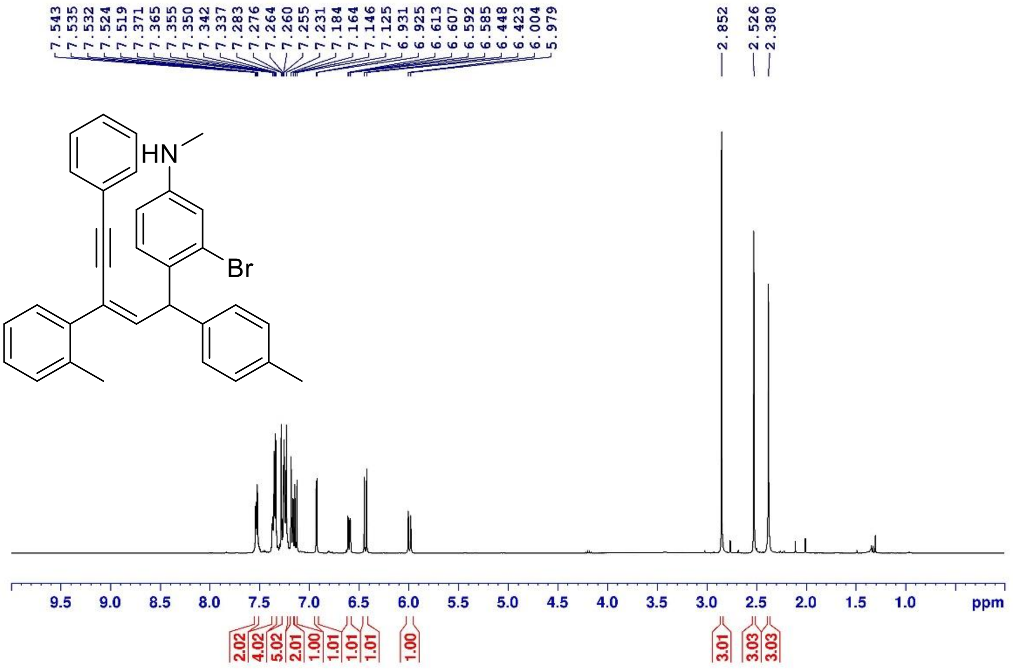


$^{13}C\{^{1}H\}$ NMR (100 MHz, $CDCl_3$) **1i**

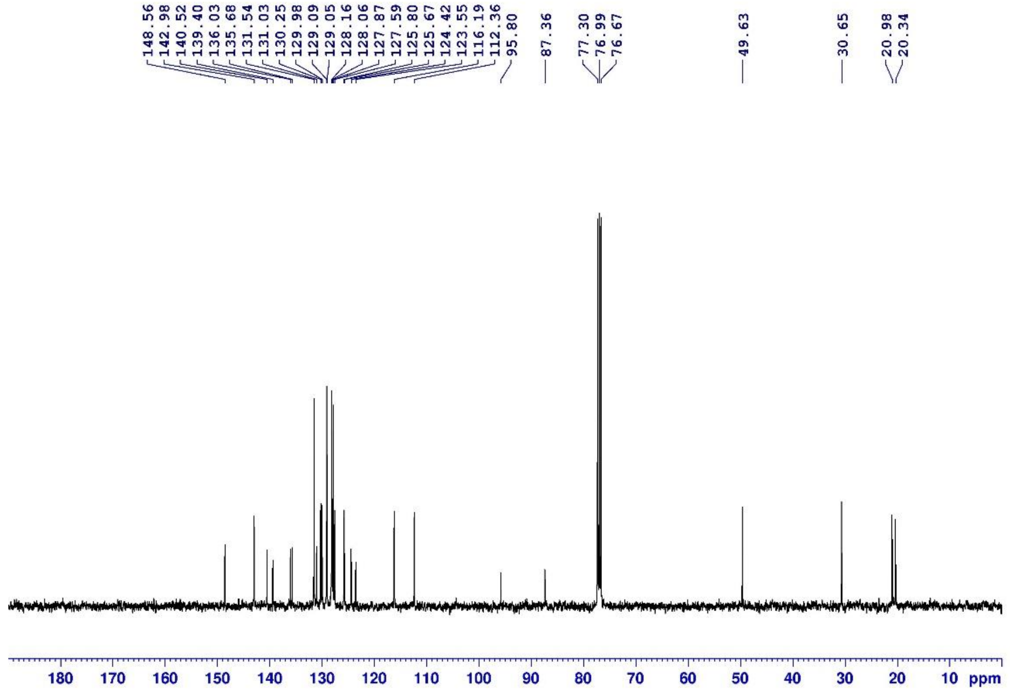

$^{1}H$ NMR (400 MHz, $CDCl_3$) Spectrum of **1j**

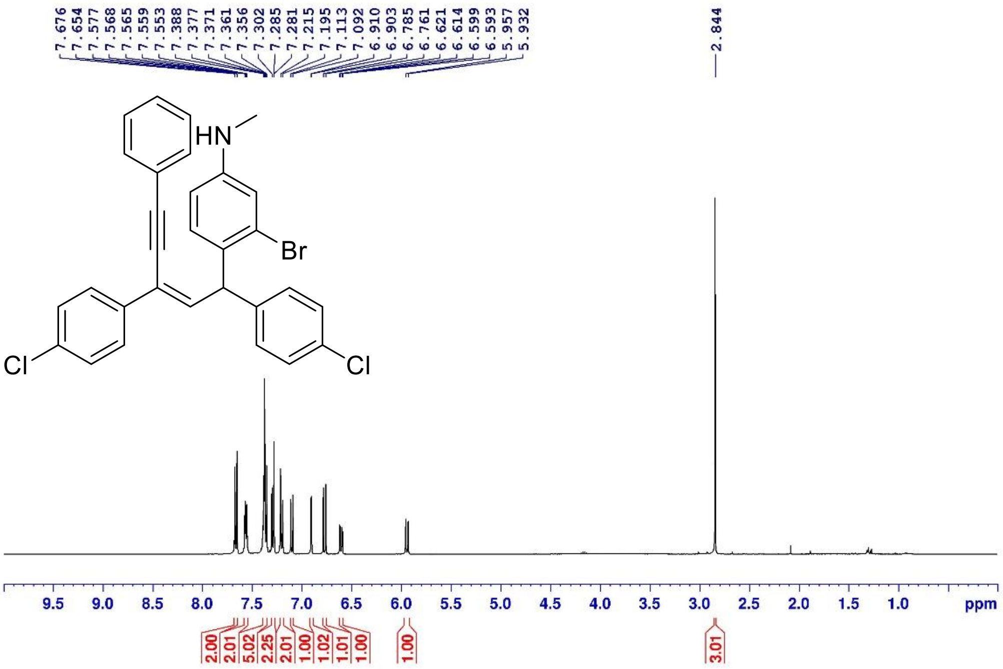


$^{13}C\{^{1}H\}$ NMR (100 MHz, $CDCl_3$) **1j**

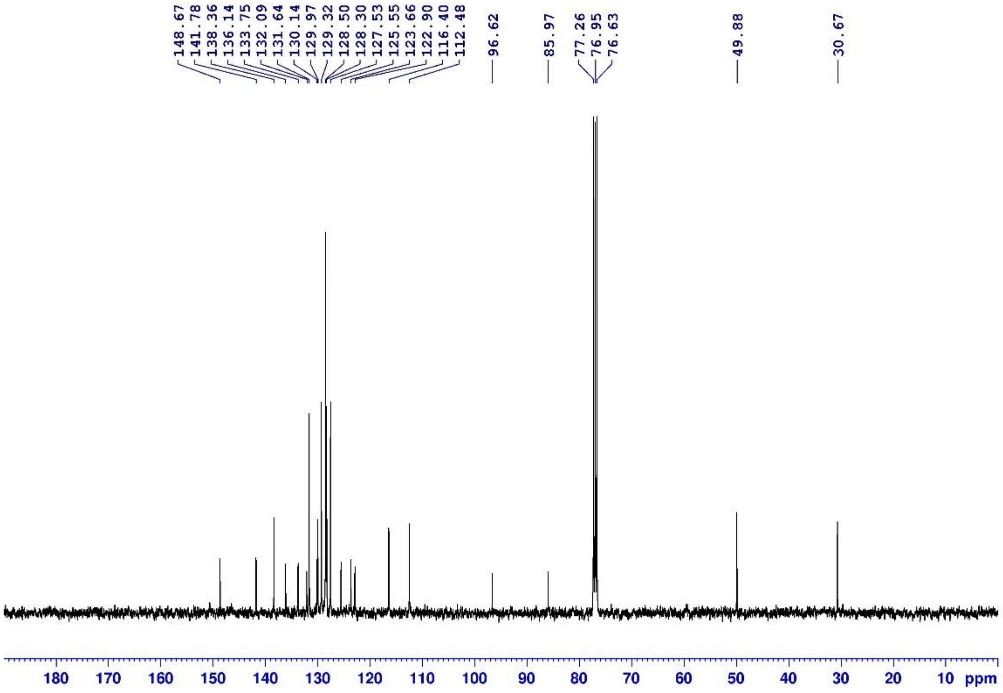

$^{1}H$ NMR (400 MHz, $CDCl_3$) Spectrum of **1k**

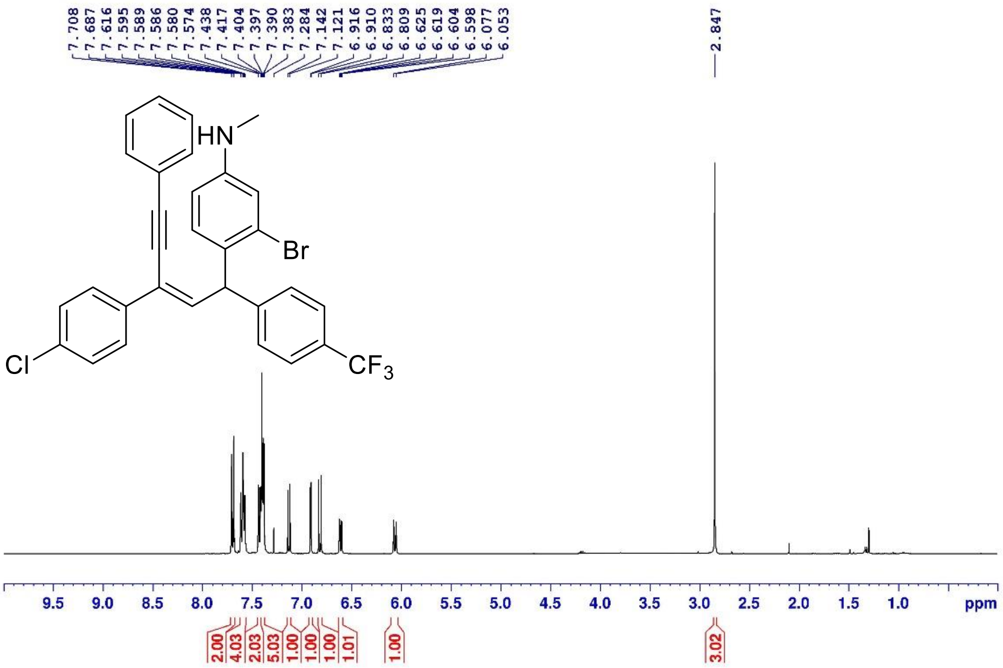

$^{13}C\{^{1}H\}$ NMR (100 MHz, $CDCl_3$) **1k**

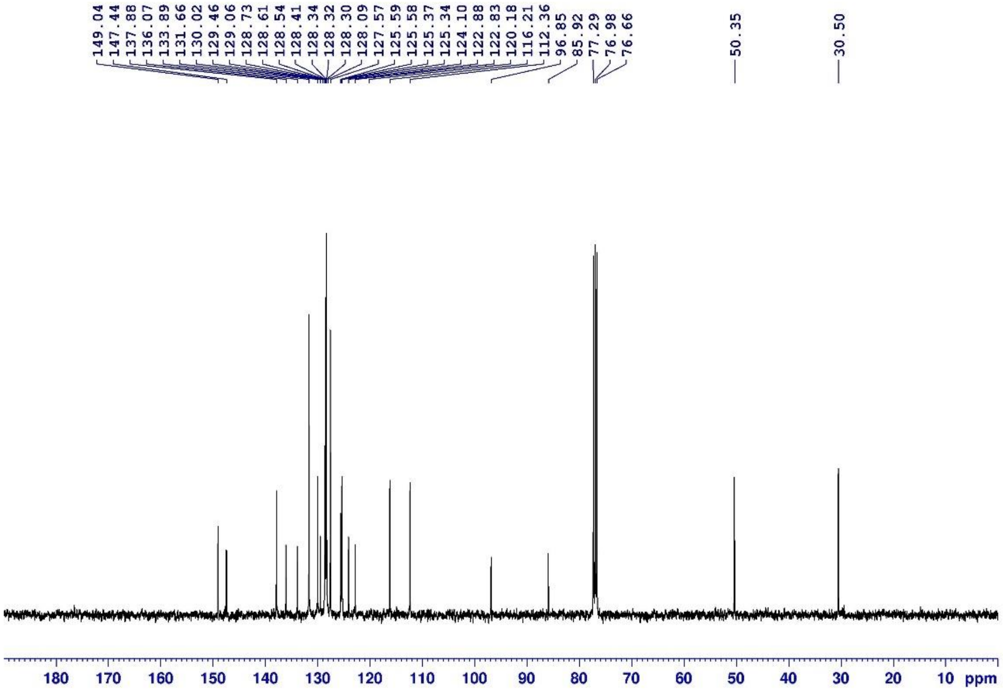

$^1$H NMR (400 MHz, $CDCl_3$) Spectrum of **1l**

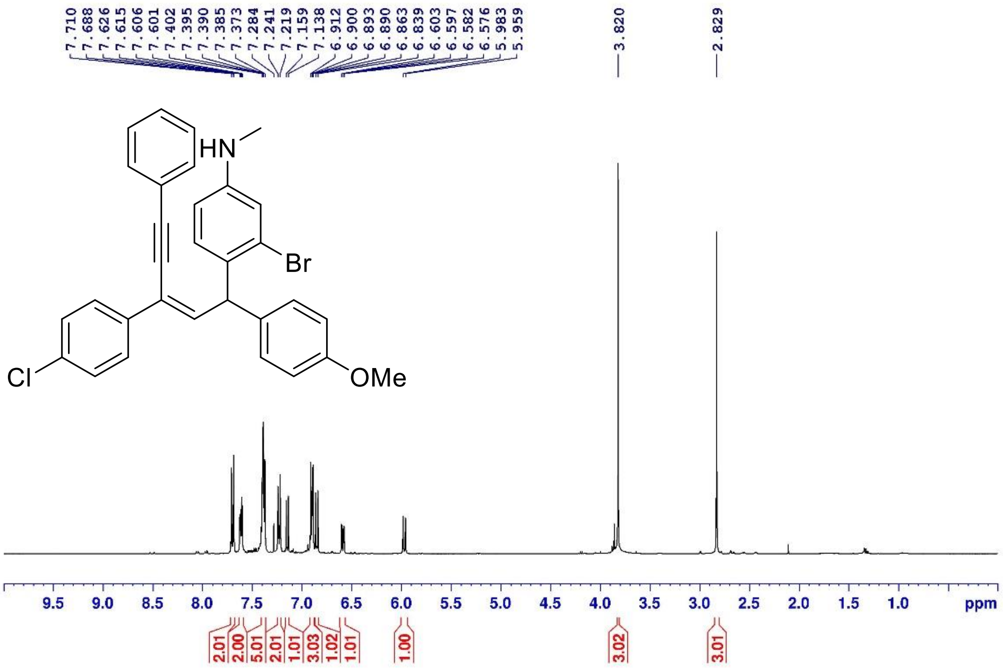


$^{13}C\{^1H\}$ NMR (100 MHz, $CDCl_3$) **1l**

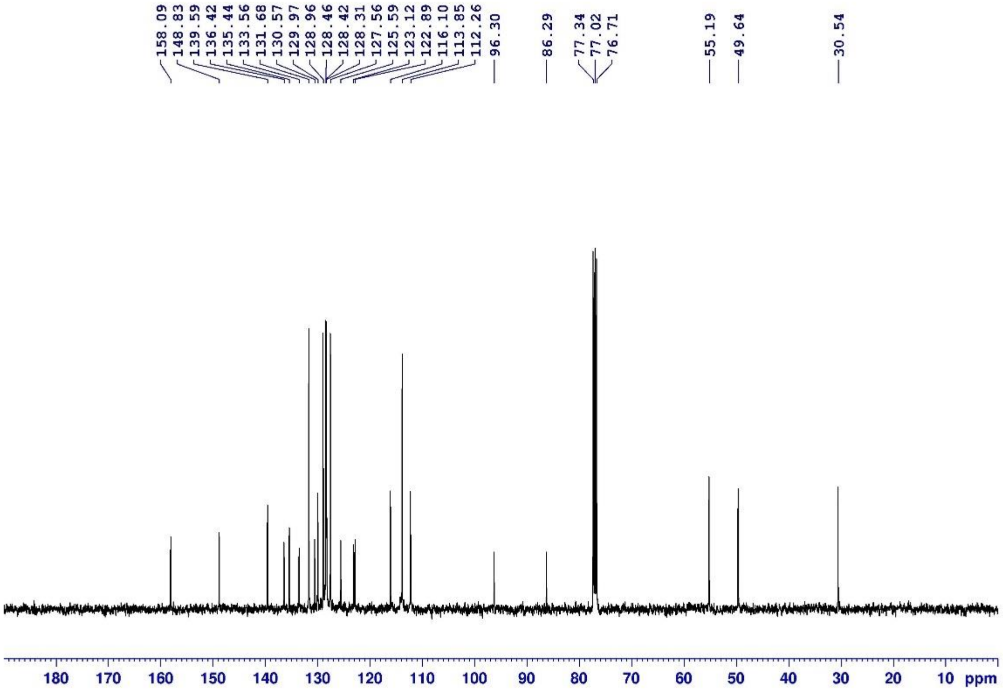

$^{1}$H NMR (400 MHz, $CDCl_3$) Spectrum of **1m**

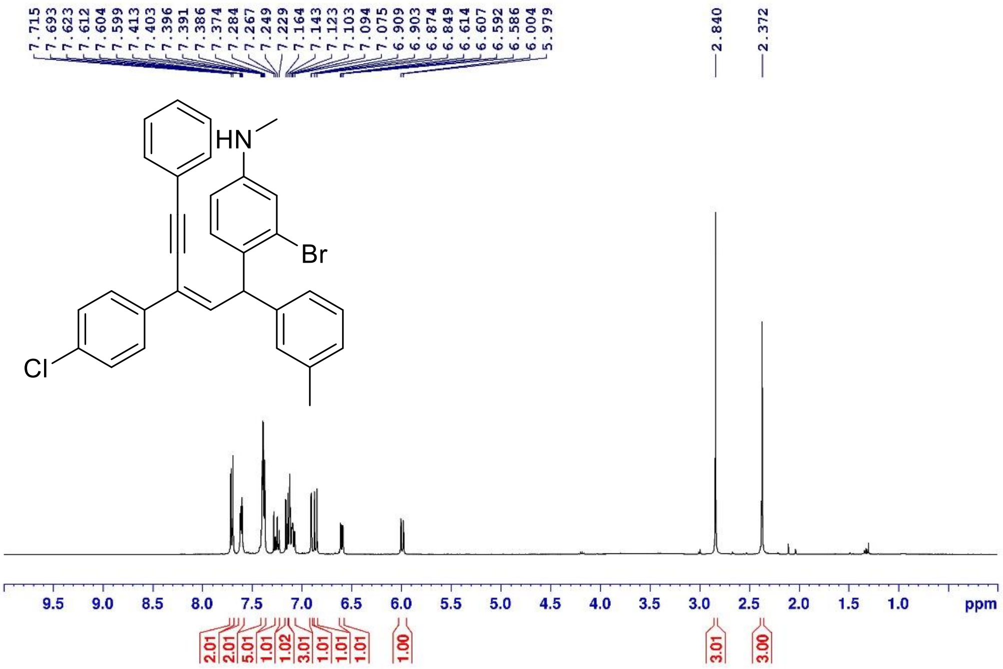

$^{13}$C{$^{1}$H} NMR (100 MHz, $CDCl_3$) **1m**

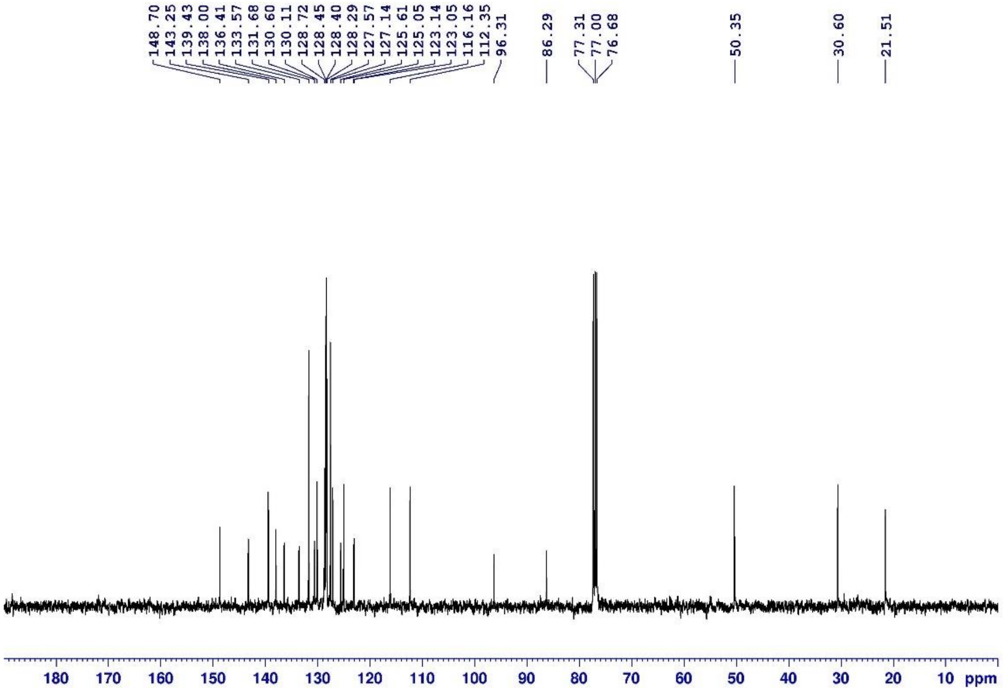

[1]1H NMR (400 MHz, $CDCl_3$) Spectrum of **1n**

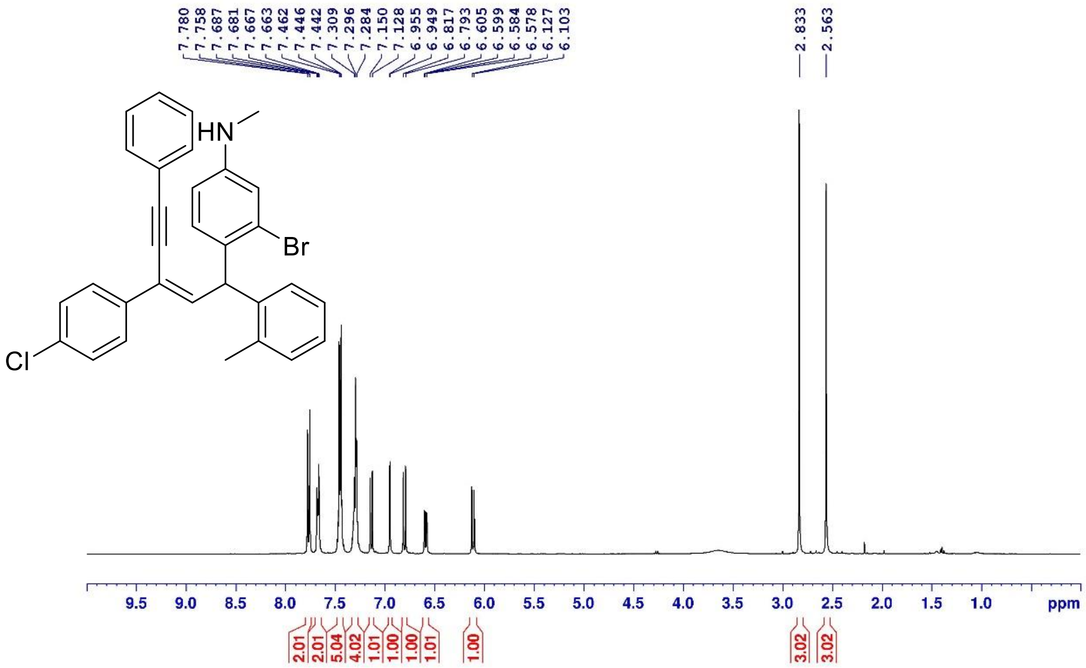

$^{13}C\{^{1}H\}$ NMR (100 MHz, $CDCl_3$) **1n**

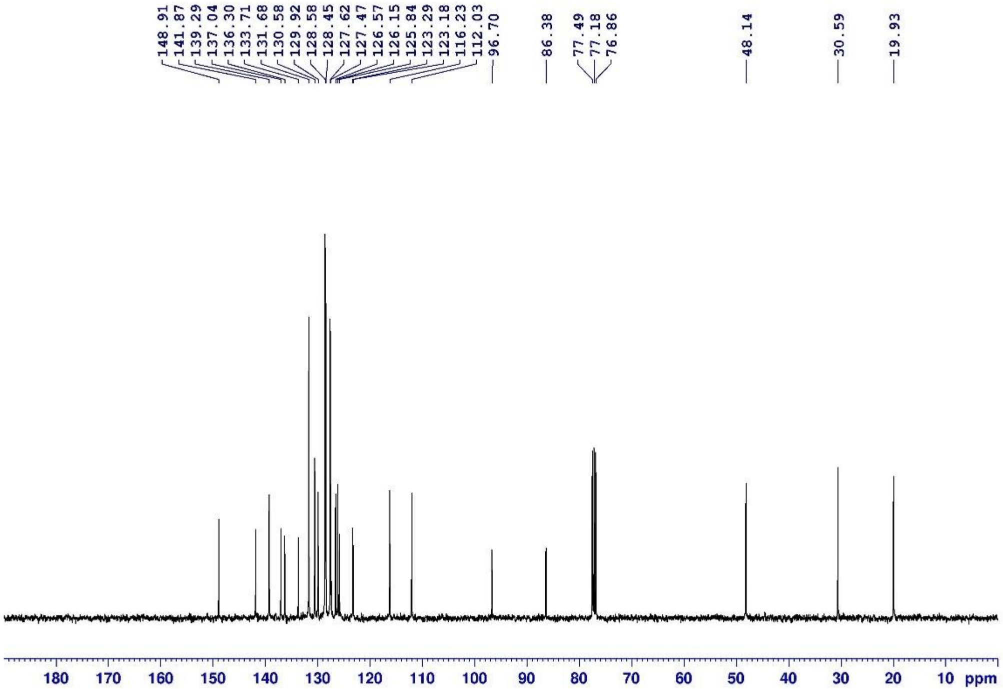

$^{1}$H NMR (400 MHz, $CDCl_3$) Spectrum of **1o**

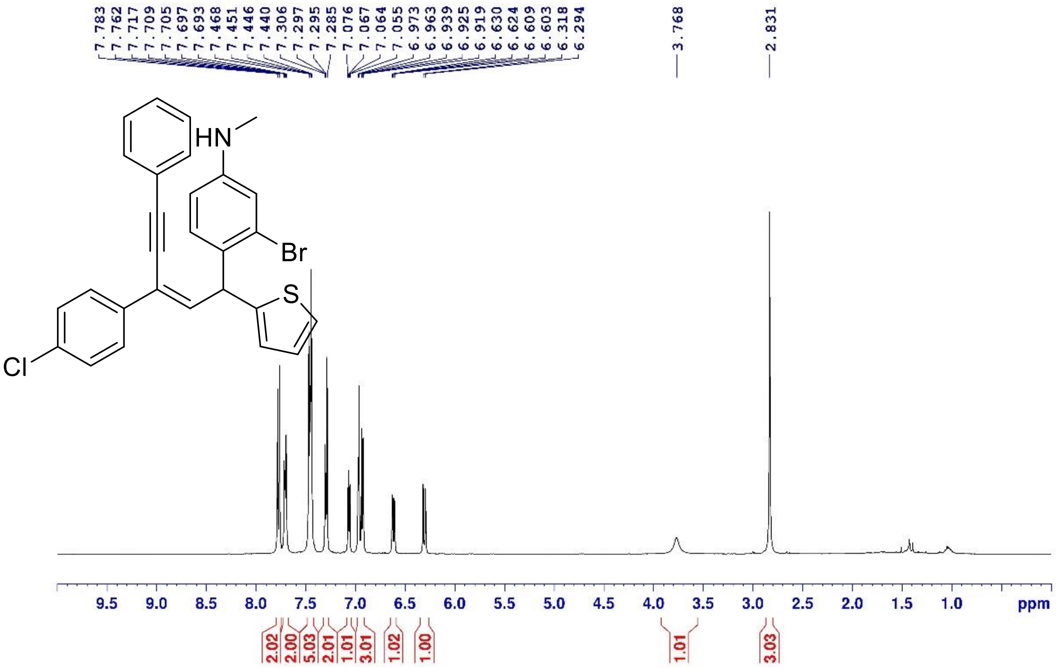


$^{13}$C{$^{1}$H} NMR (100 MHz, $CDCl_3$) **1o**

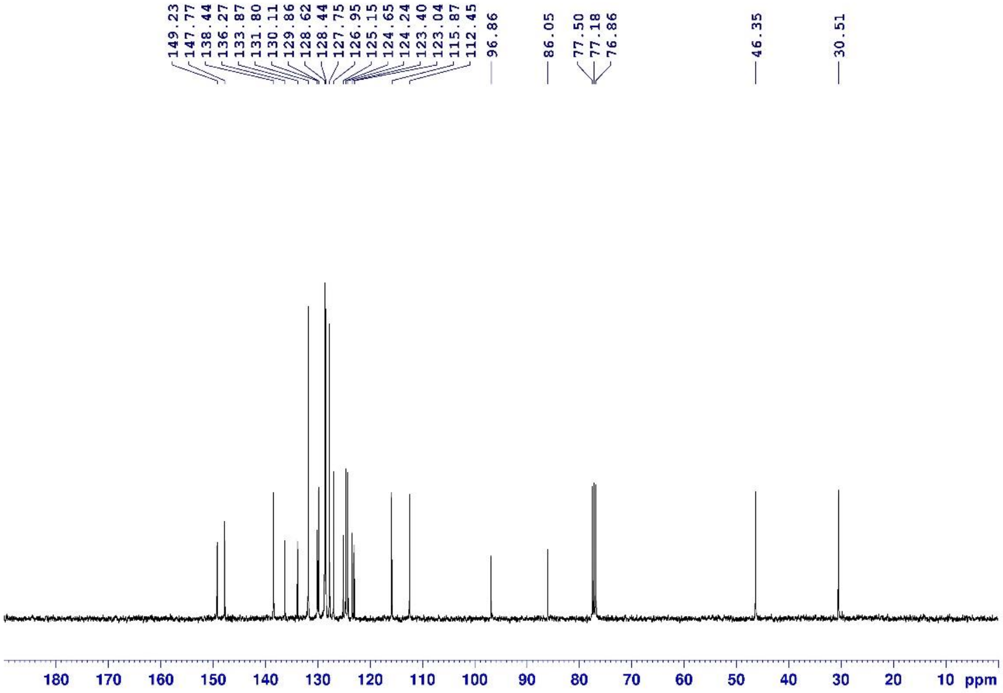

$^{1}$H NMR (400 MHz, $CDCl_3$) Spectrum of **1p**

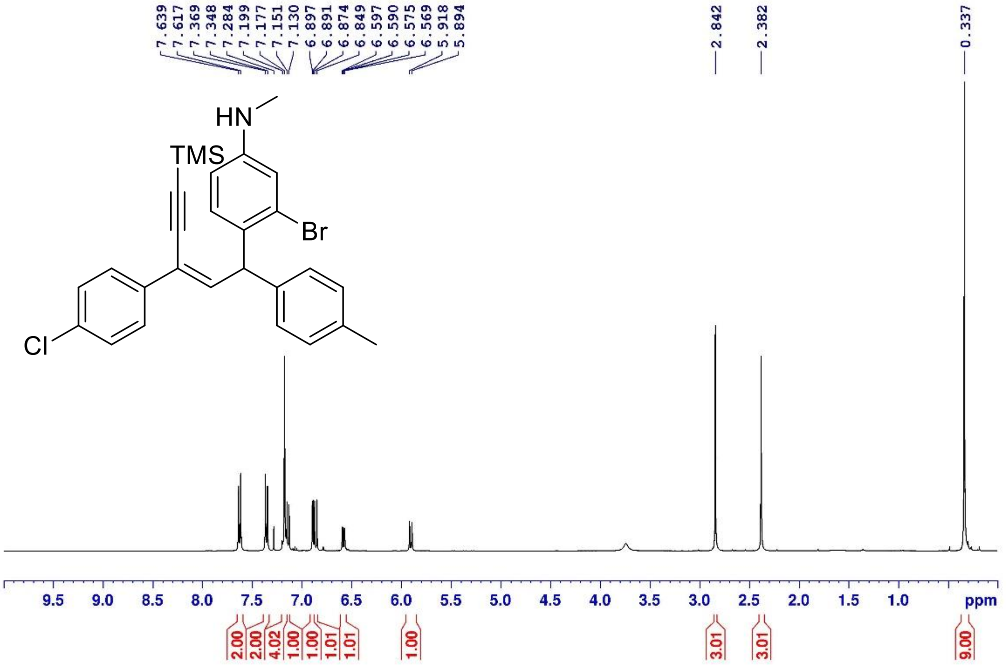


$^{13}$C{$^{1}$H} NMR (100 MHz, $CDCl_3$) **1p**

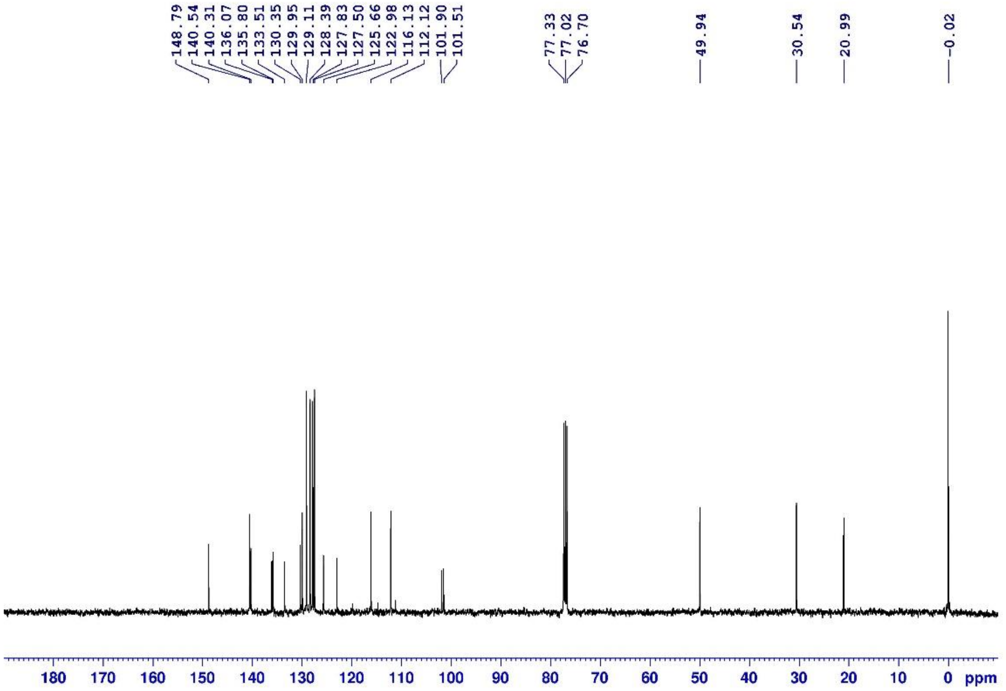

$^{1}$H NMR (400 MHz, $CDCl_3$) Spectrum of **1q**

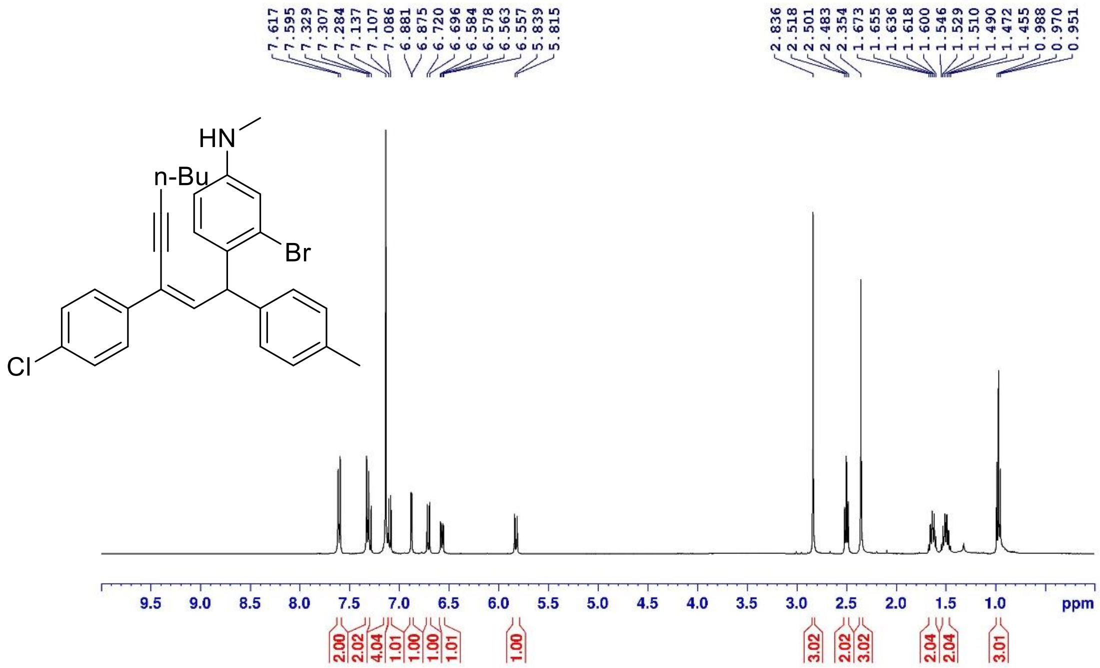


$^{13}$C{$^{1}$H} NMR (100 MHz, $CDCl_3$) **1q**

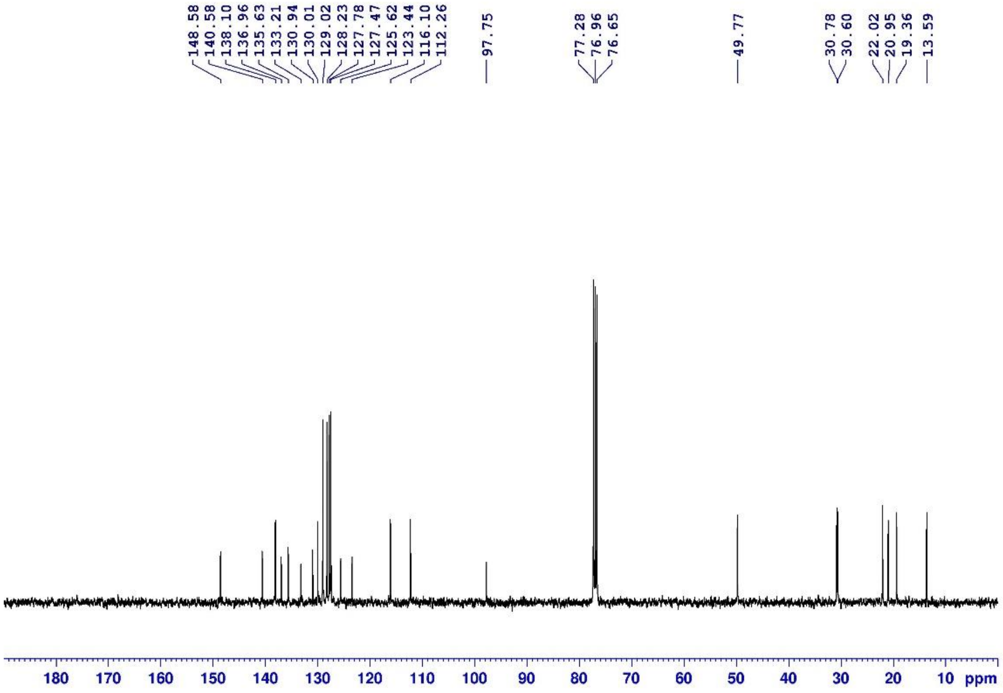

$^{1}$H NMR (400 MHz, $CDCl_3$) Spectrum of **1r**

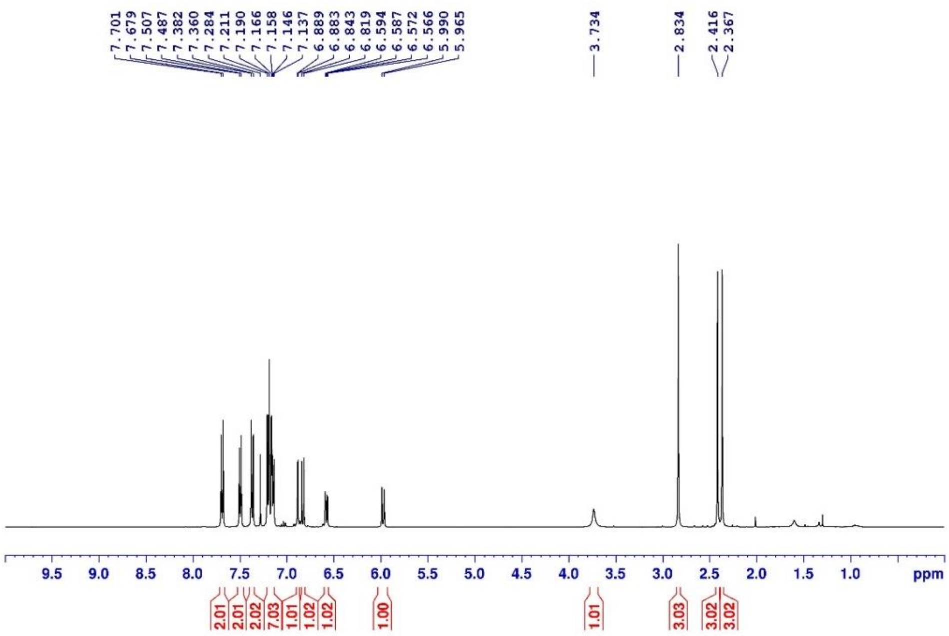

$^{13}$C{$^{1}$H} NMR (100 MHz, $CDCl_3$) **1r**

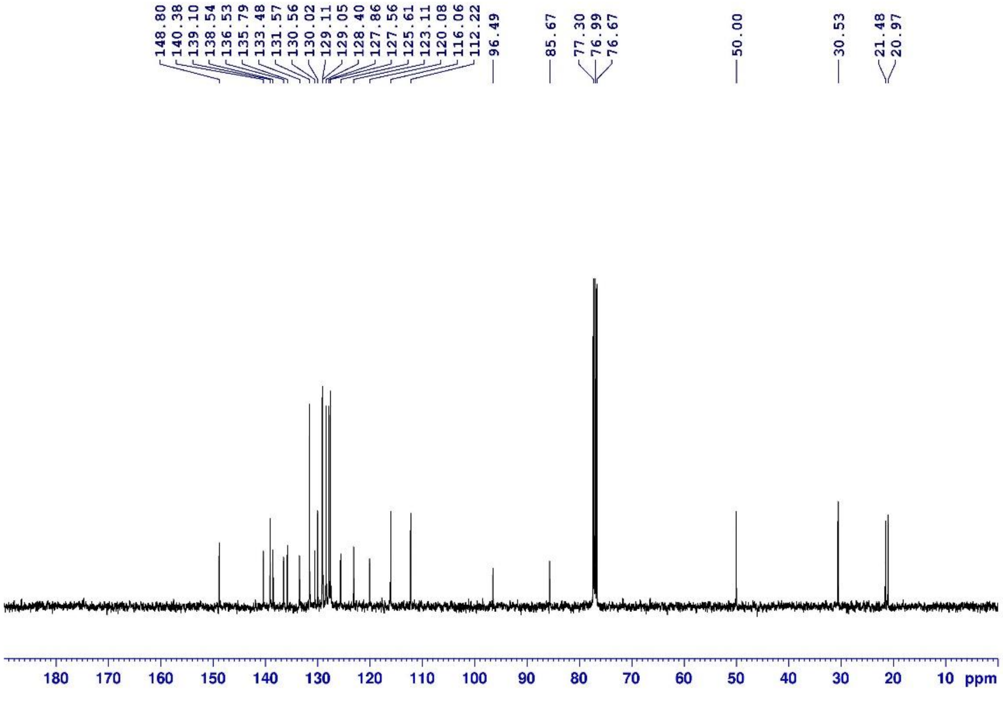

$^1$H NMR (400 MHz, $CDCl_3$) Spectrum of **1s**

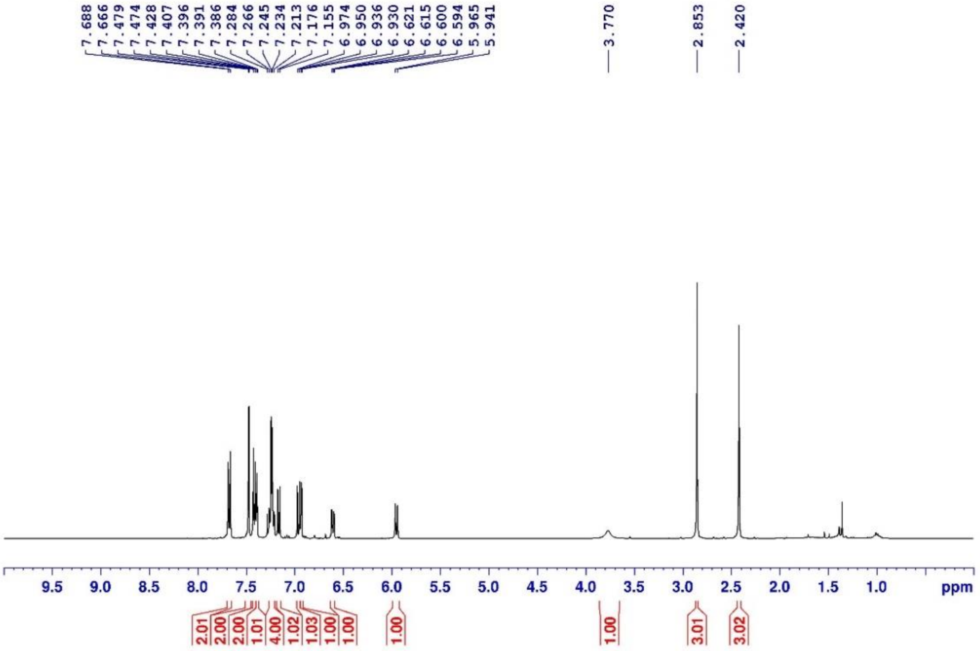


$^{13}C\{^1H\}$ NMR (100 MHz, $CDCl_3$) **1s**

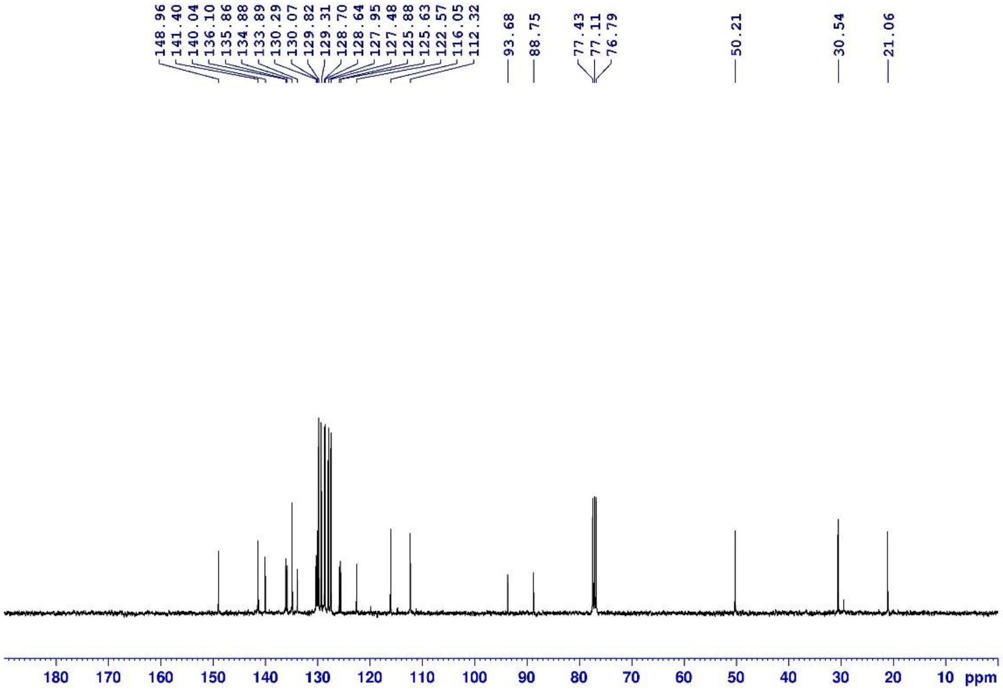

**¹H NMR (400 MHz, $CDCl_3$) Spectrum of 2a**

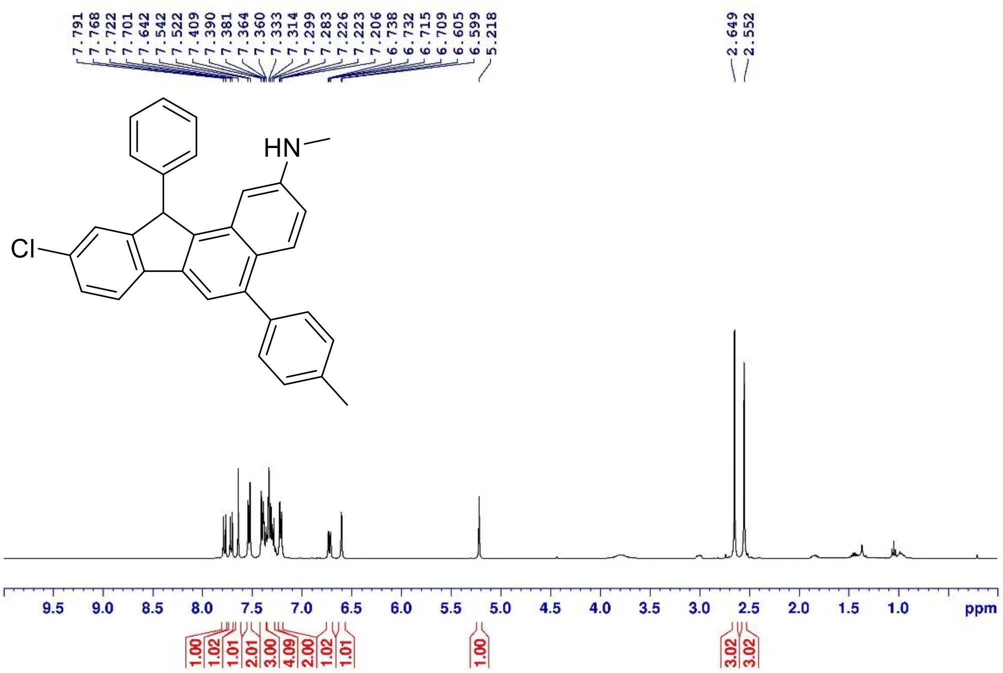


$^{13}C\{^1H\}$ NMR (100 MHz, $CDCl_3$) **2a**

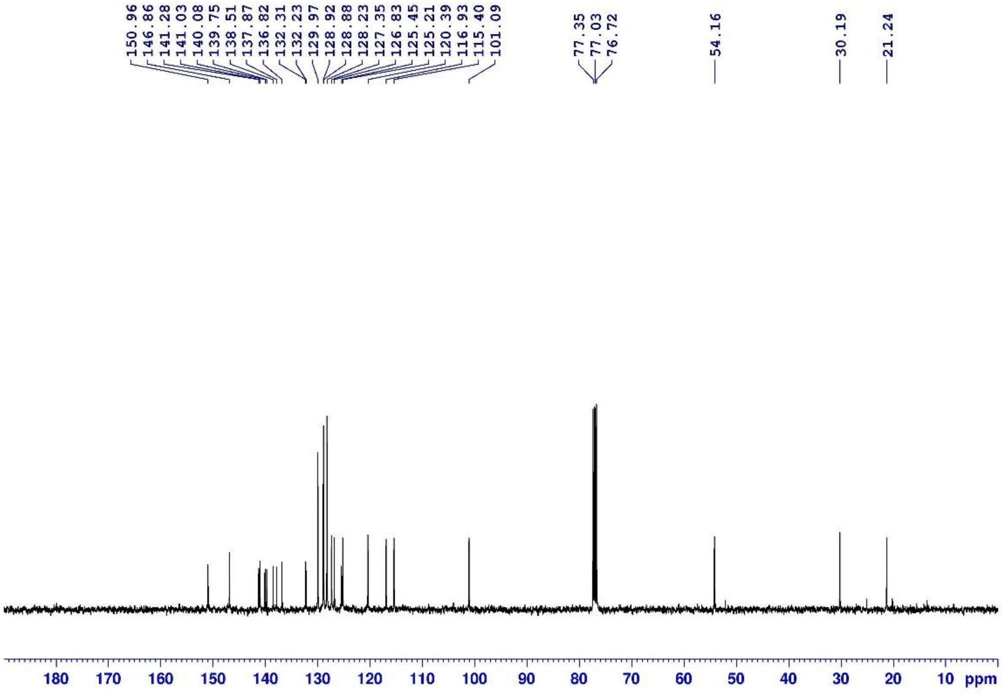

$^1$H NMR (400 MHz, $CDCl_3$) Spectrum of **2b**

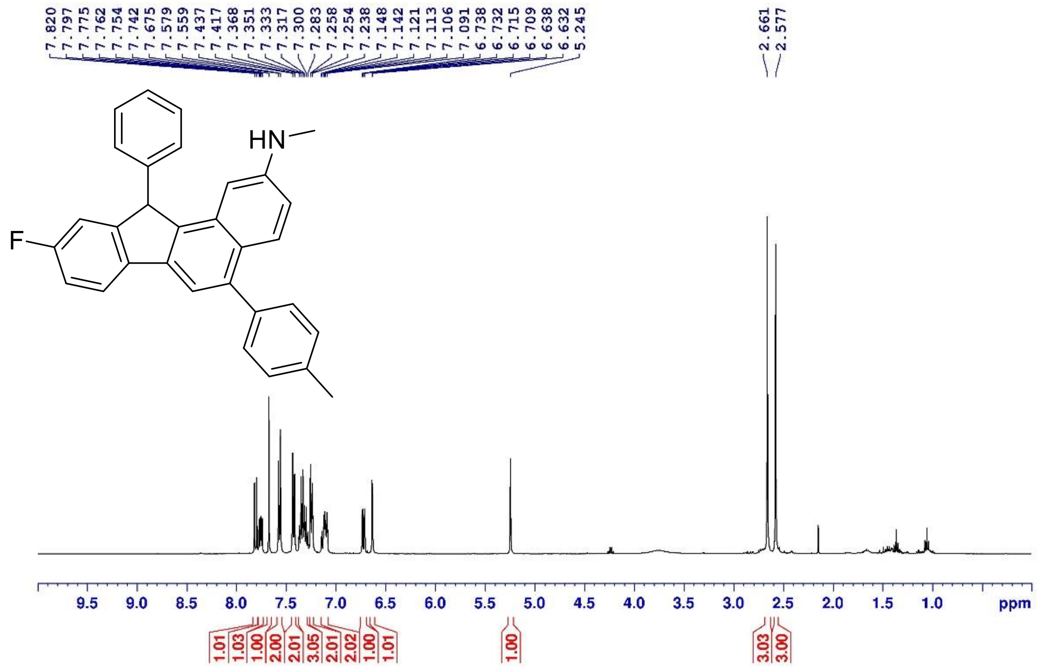


$^{13}C\{^1H\}$ NMR (100 MHz, $CDCl_3$) **2b**

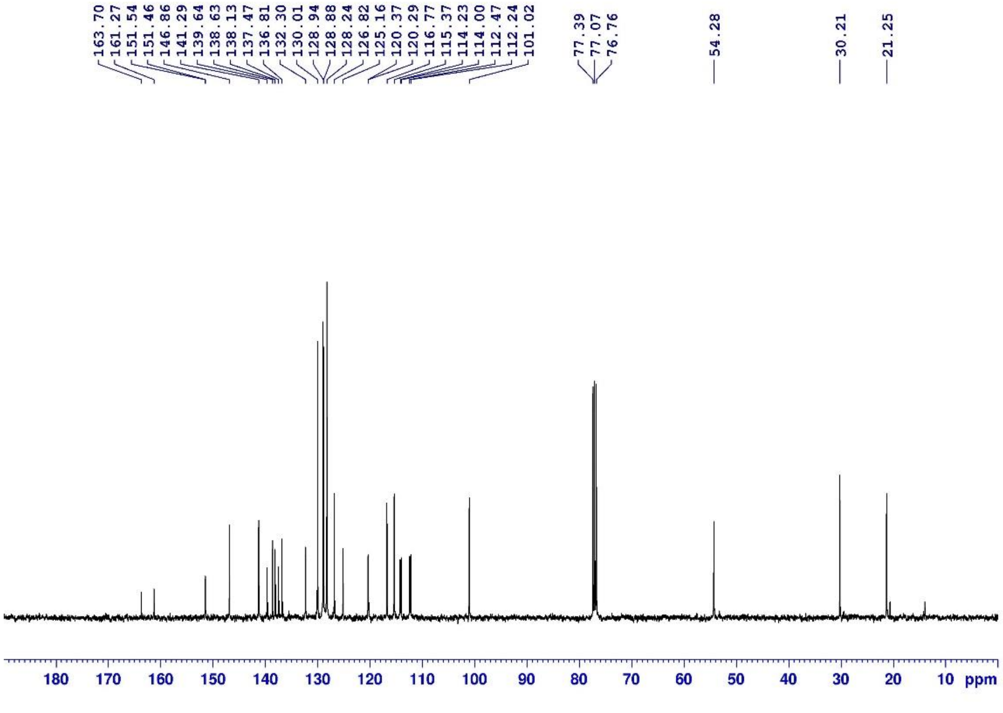

$^1$H NMR (400 MHz, $CDCl_3$) Spectrum of **2d**

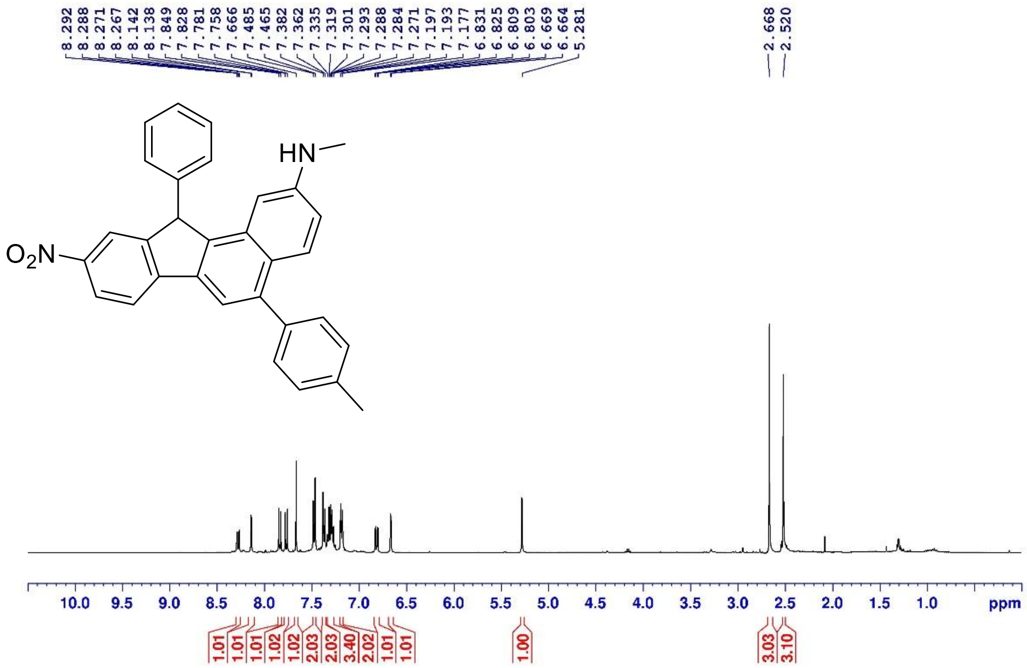

$^{13}C\{^1H\}$ NMR (100 MHz, $CDCl_3$) **2d**

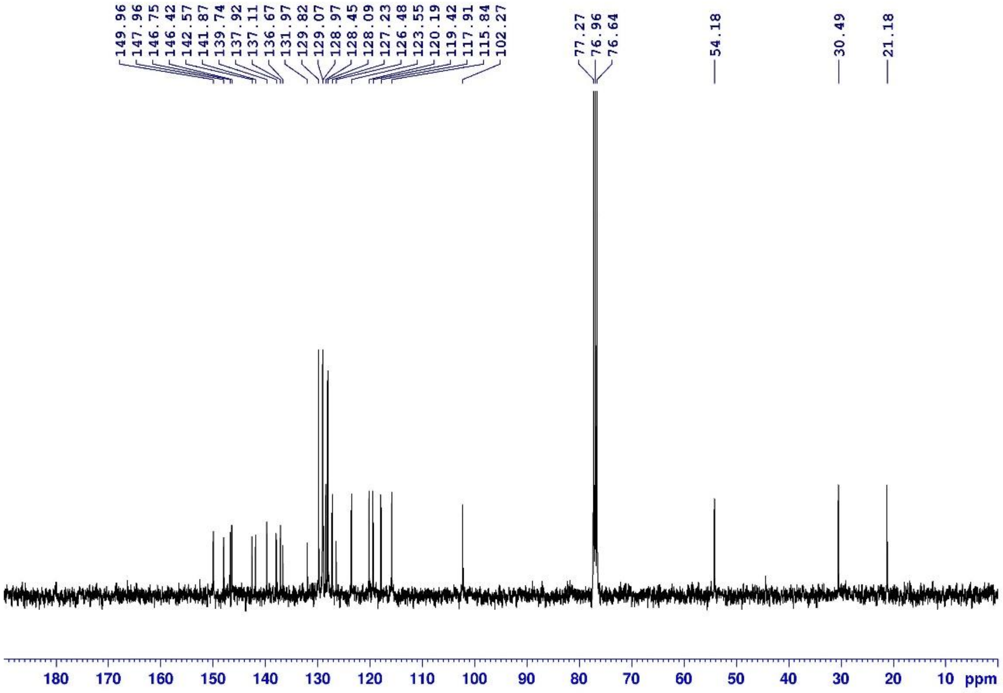

$^{1}H$ NMR (400 MHz, $CDCl_3$) Spectrum of **2e**

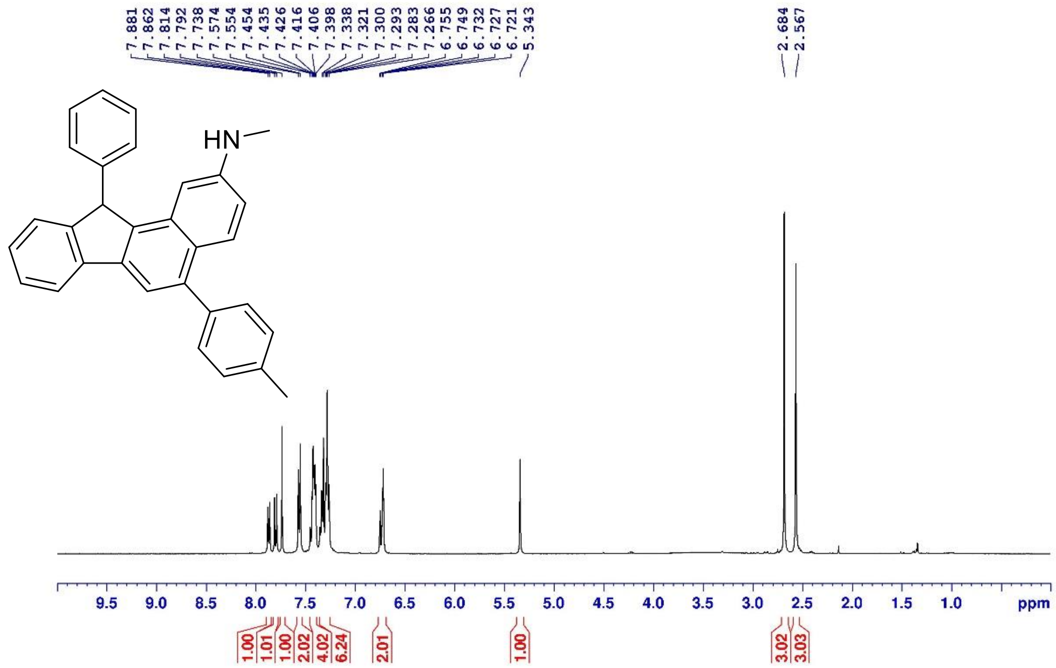


$^{13}C\{^{1}H\}$ NMR (100 MHz, $CDCl_3$) **2e**

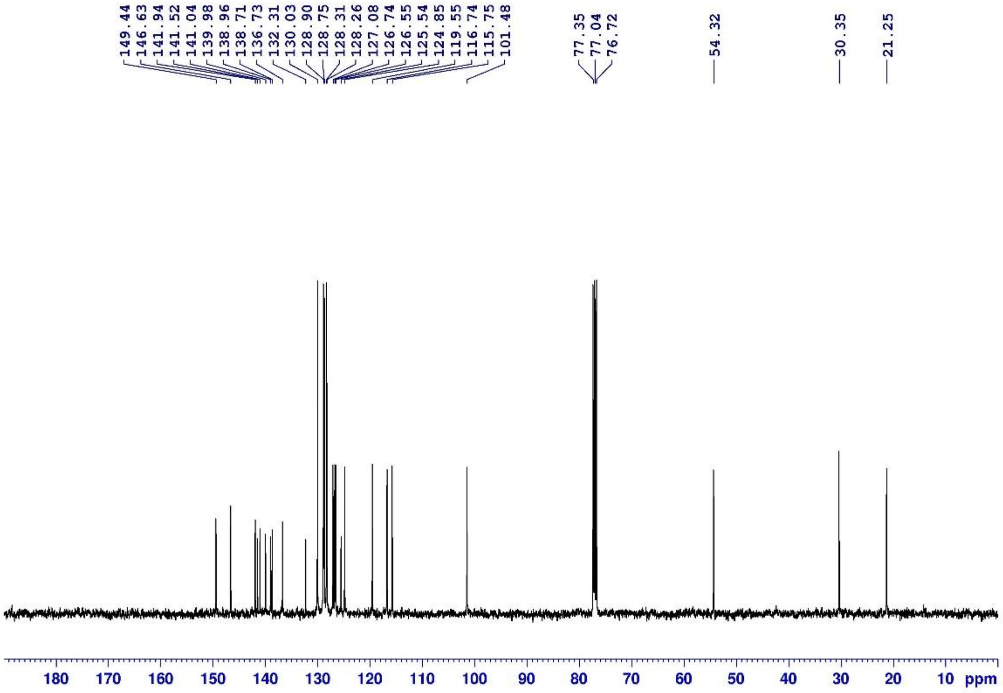

$^{1}H$ NMR (400 MHz, $CDCl_3$) Spectrum of **2f**

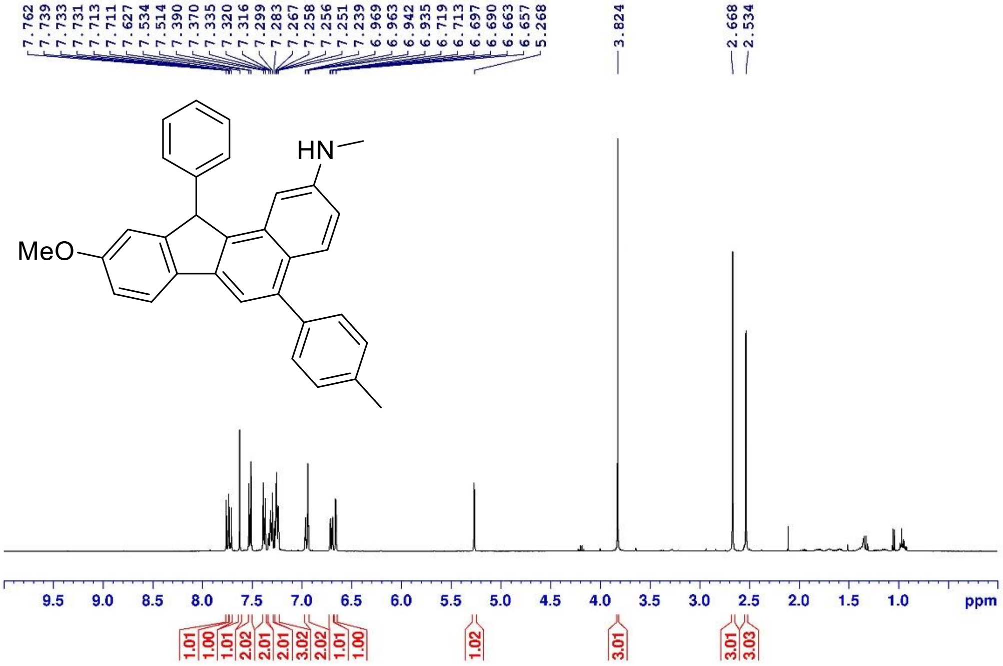


$^{13}C\{^{1}H\}$ NMR (100 MHz, $CDCl_3$) **2f**

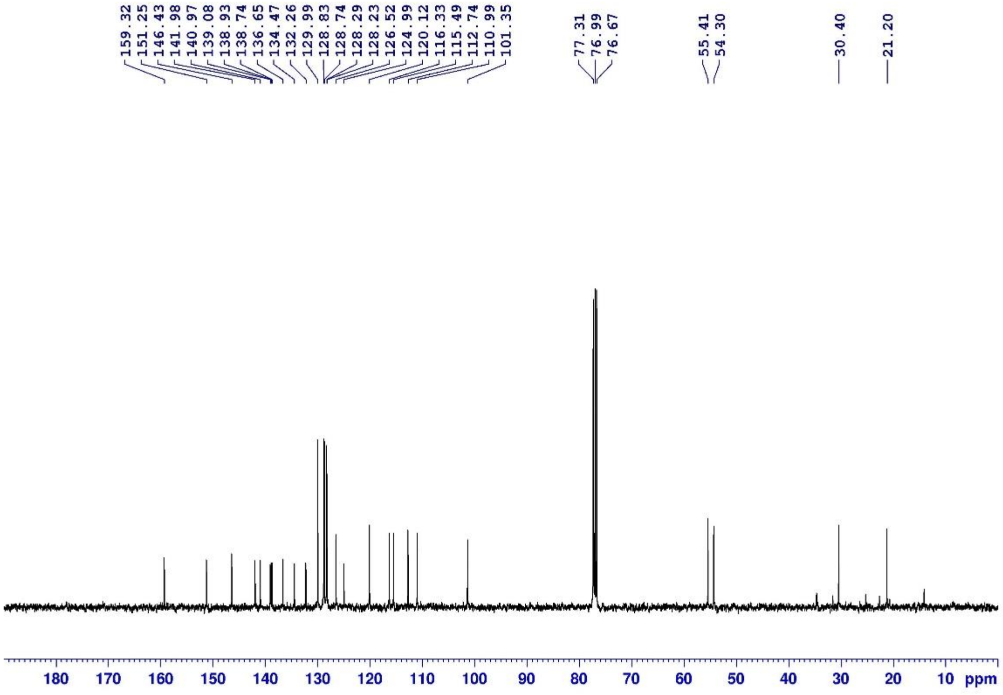

**¹H NMR (400 MHz, $CDCl_3$) Spectrum of 2g**

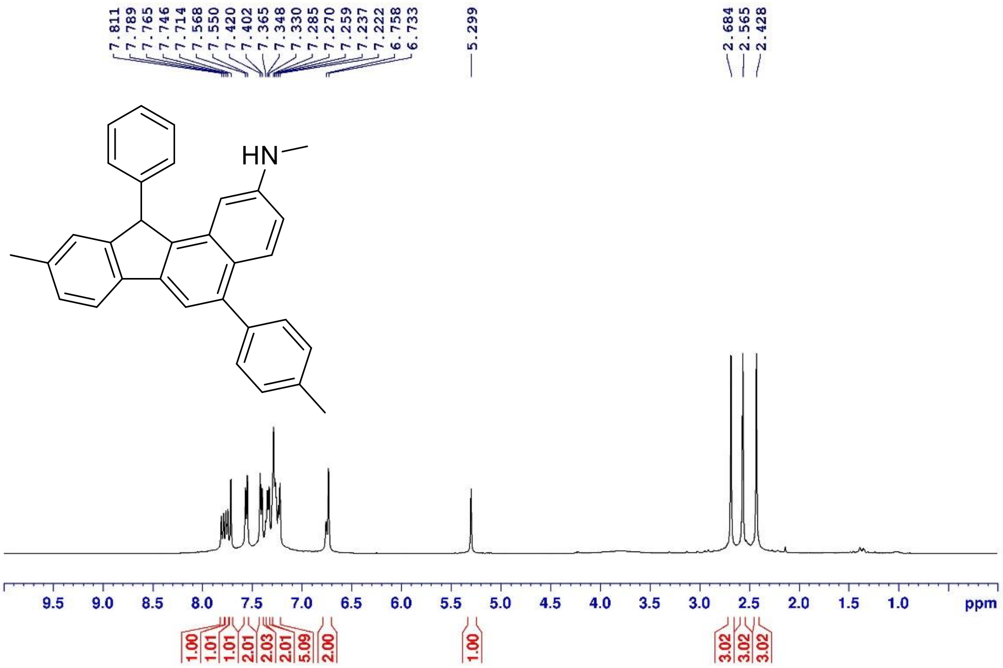


$^{13}C\{^1H\}$ NMR (100 MHz, $CDCl_3$) **2g**

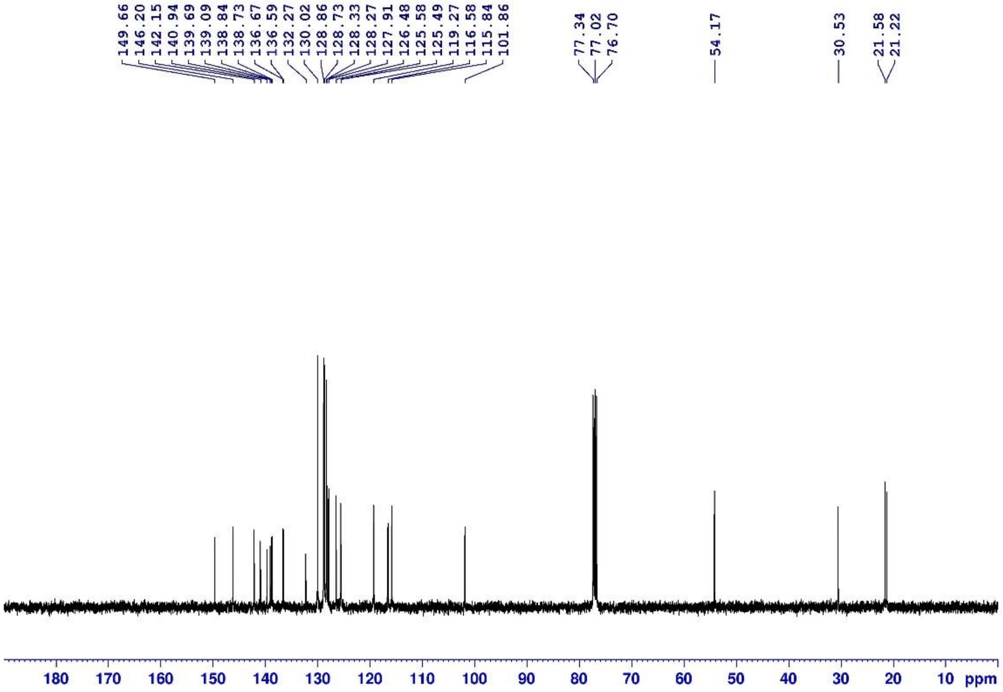

$^{1}H$ NMR (400 MHz, $CDCl_3$) Spectrum of **2h** + **2h’**

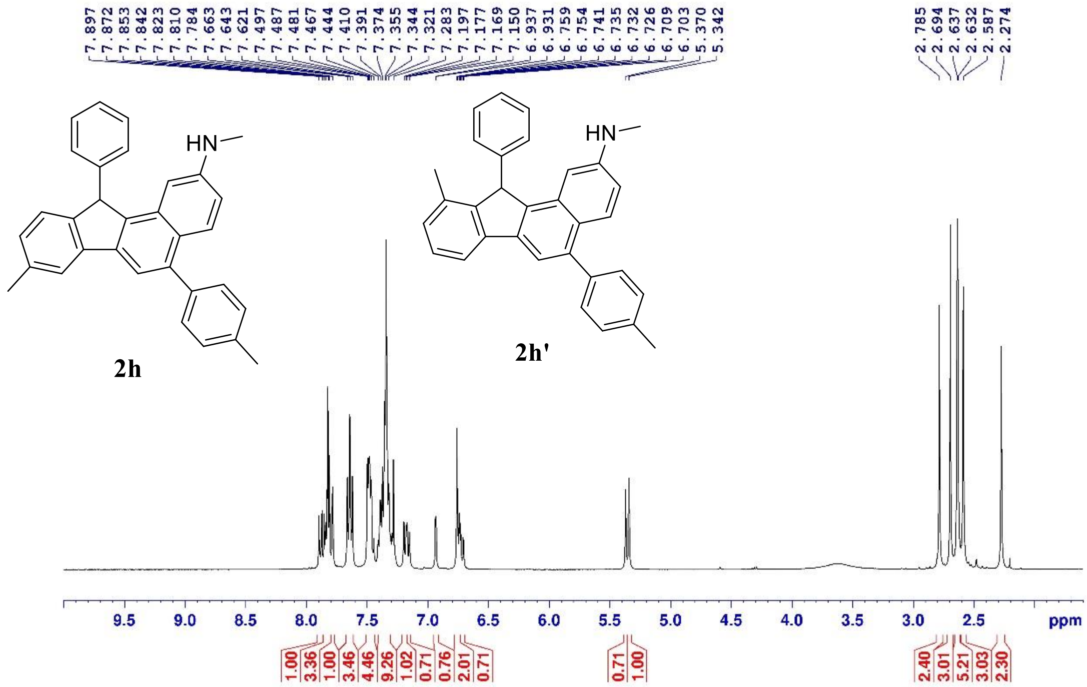


$^{13}C\{^{1}H\}$ NMR (100 MHz, $CDCl_3$) **2h** + **2h’**

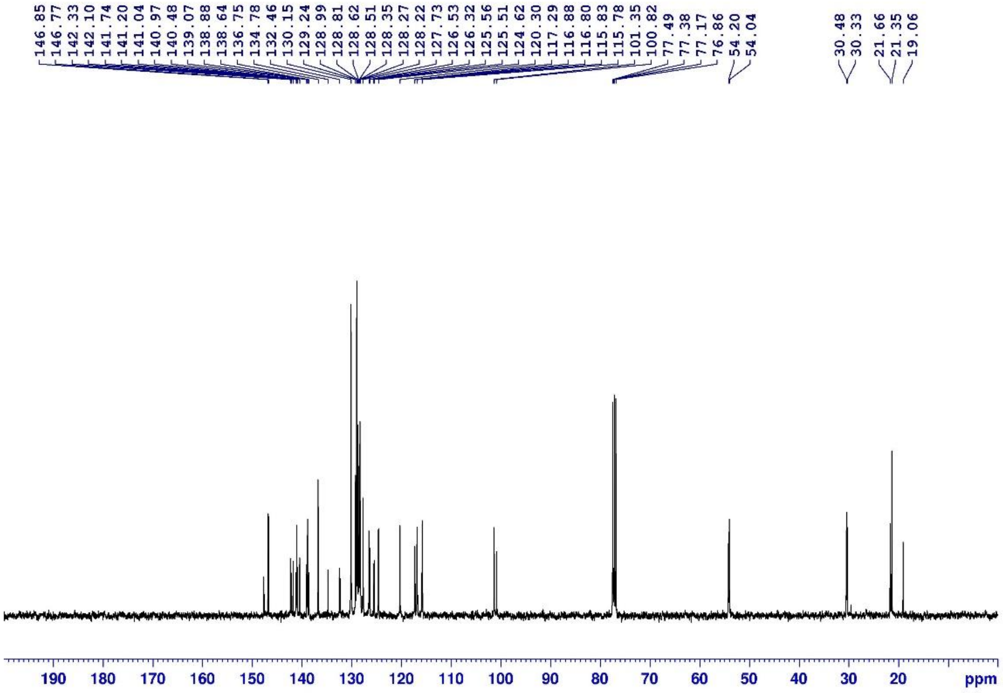

$^{1}$H NMR (400 MHz, $CDCl_3$) Spectrum of **2i**

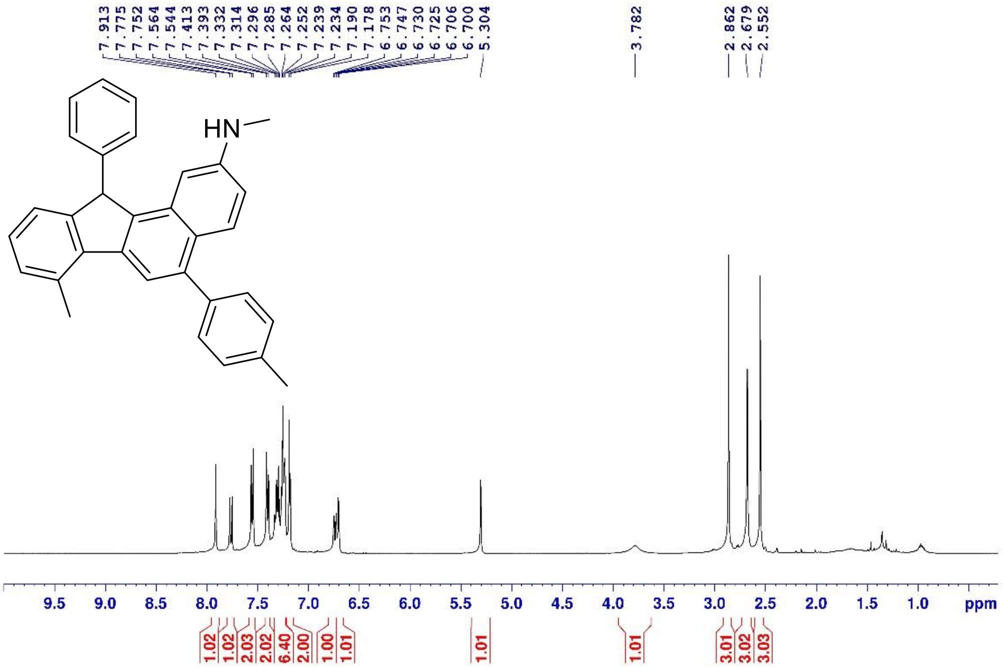


$^{13}$C{$^{1}$H} NMR (100 MHz, $CDCl_3$) **2i**

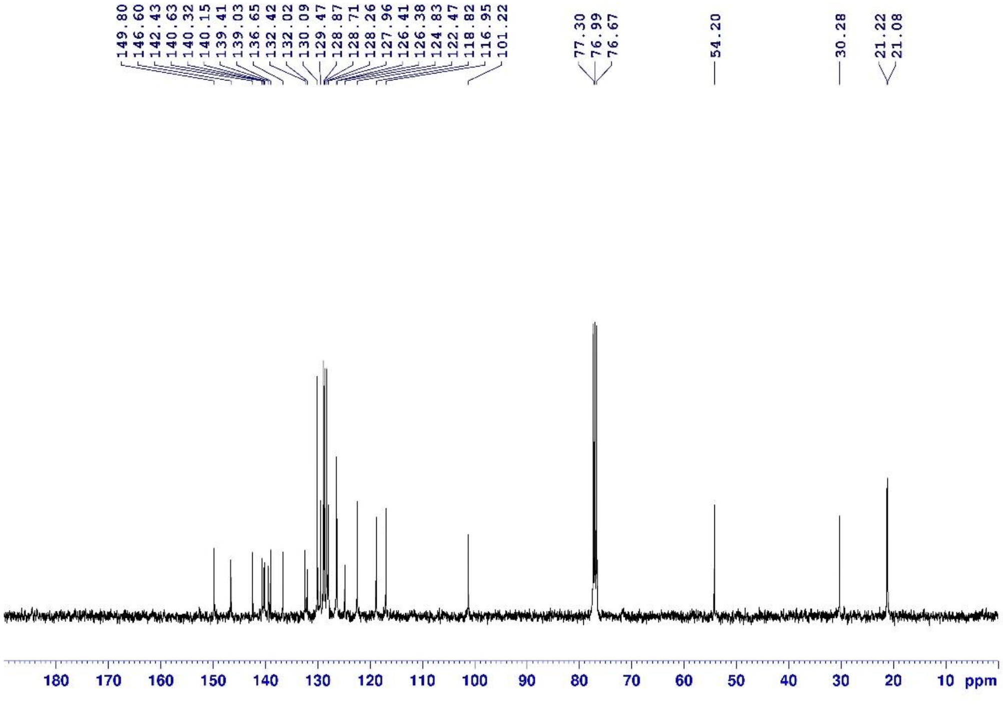

$^1$H NMR (400 MHz, $CDCl_3$) Spectrum of **2j**

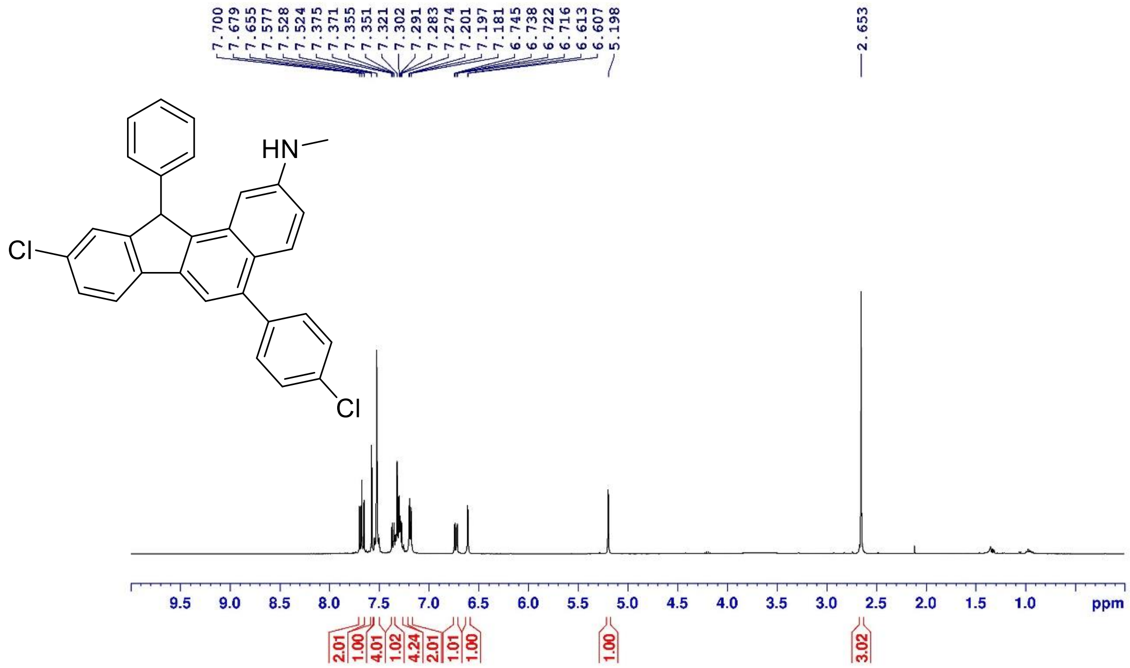


$^{13}C\{^1H\}$ NMR (100 MHz, $CDCl_3$) **2j**

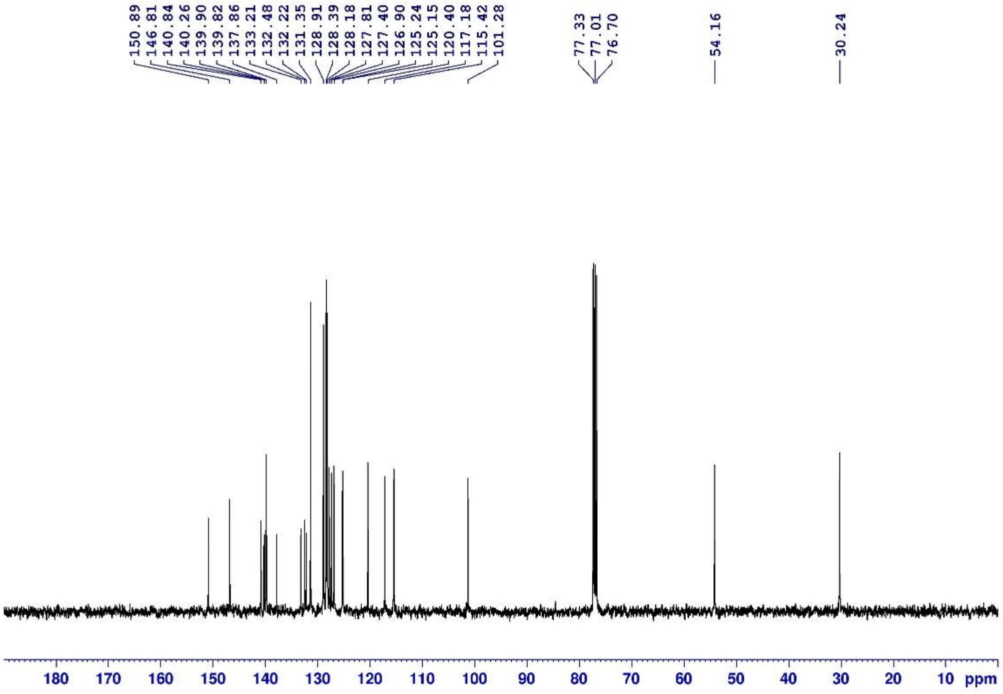

$^{1}$H NMR (400 MHz, $CDCl_3$) Spectrum of **2k**

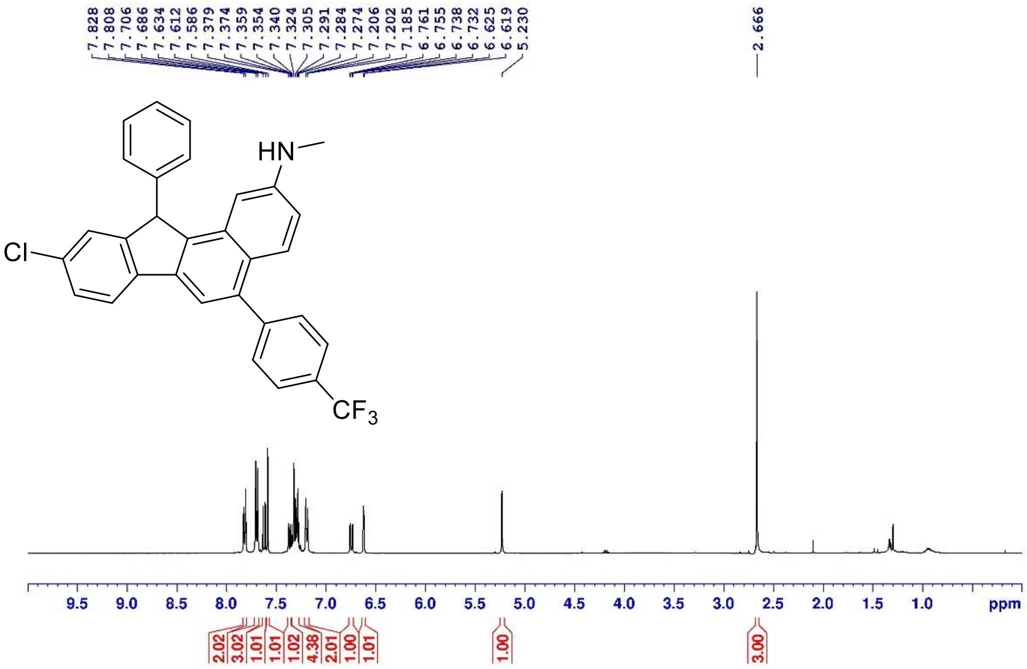


$^{13}$C{$^{1}$H} NMR (100 MHz, $CDCl_3$) **2k**

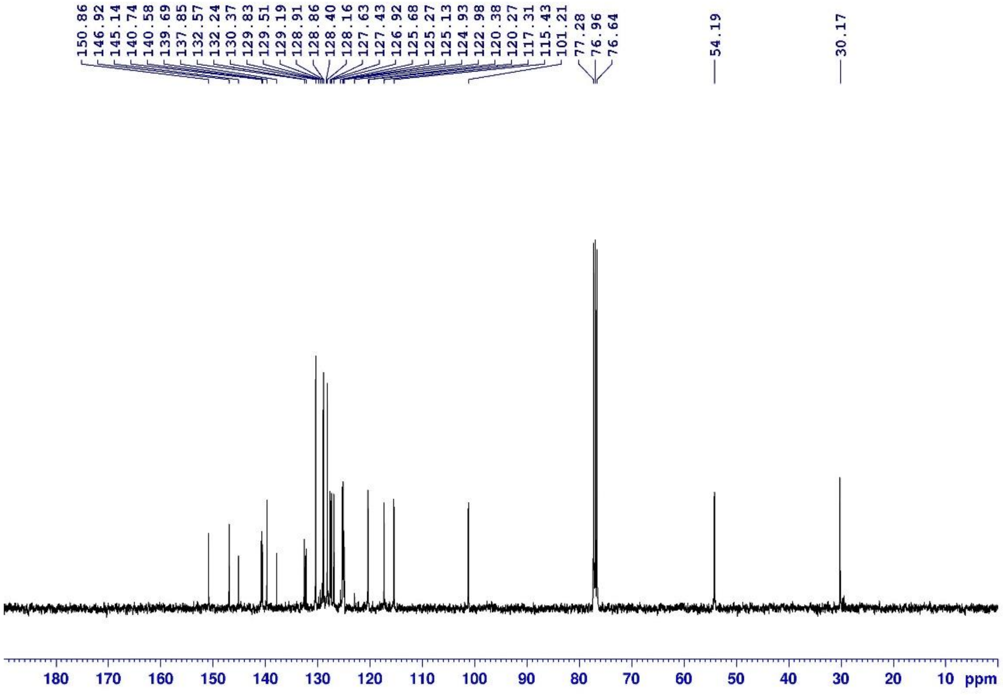

$^1$H NMR (400 MHz, $CDCl_3$) Spectrum of **2l**

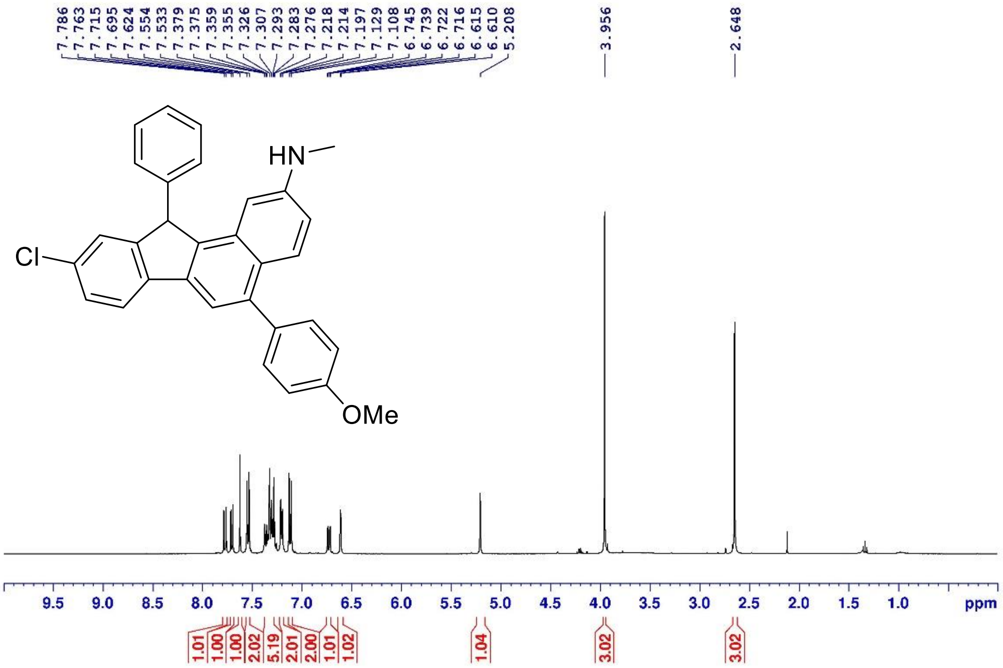


$^{13}$C{$^1$H} NMR (100 MHz, $CDCl_3$) **2l**

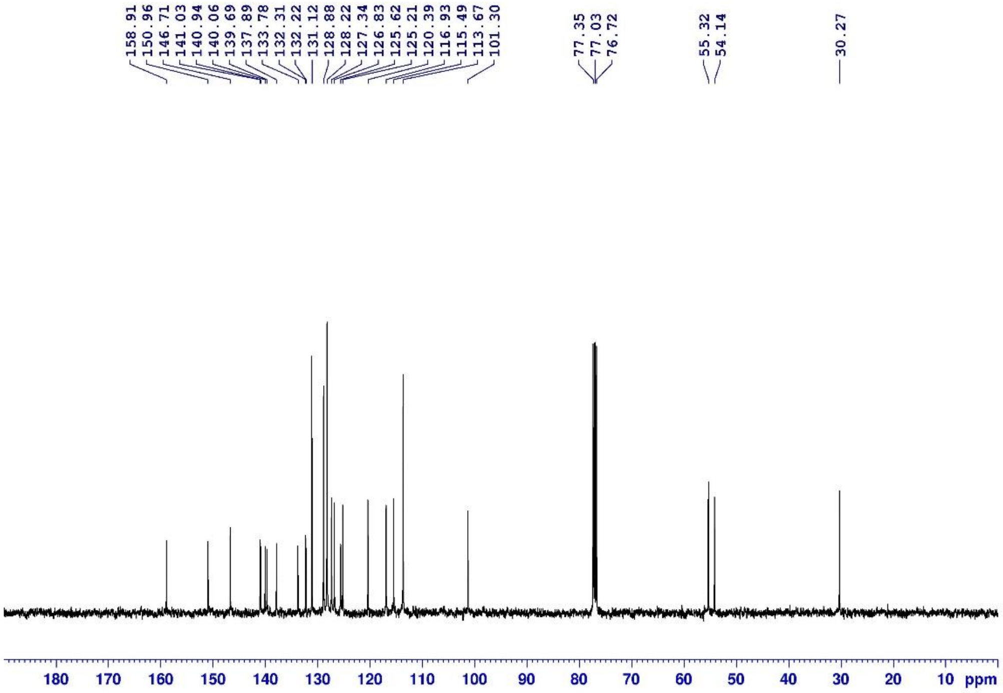

$^1$H NMR (400 MHz, $CDCl_3$) Spectrum of **2m**

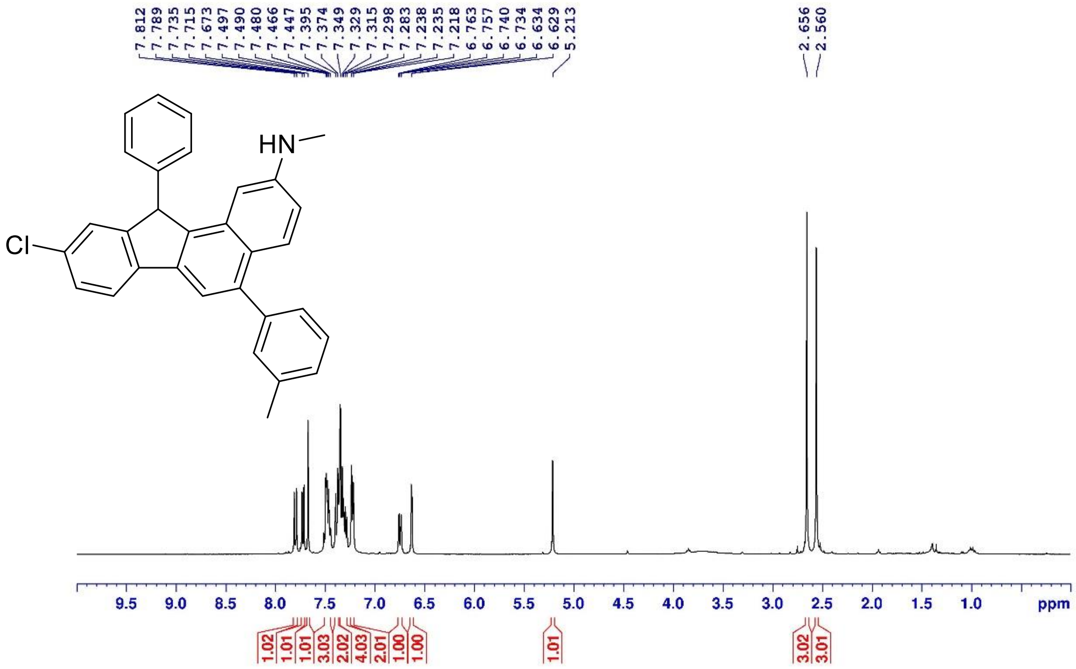


$^{13}C\{^1H\}$ NMR (100 MHz, $CDCl_3$) **2m**

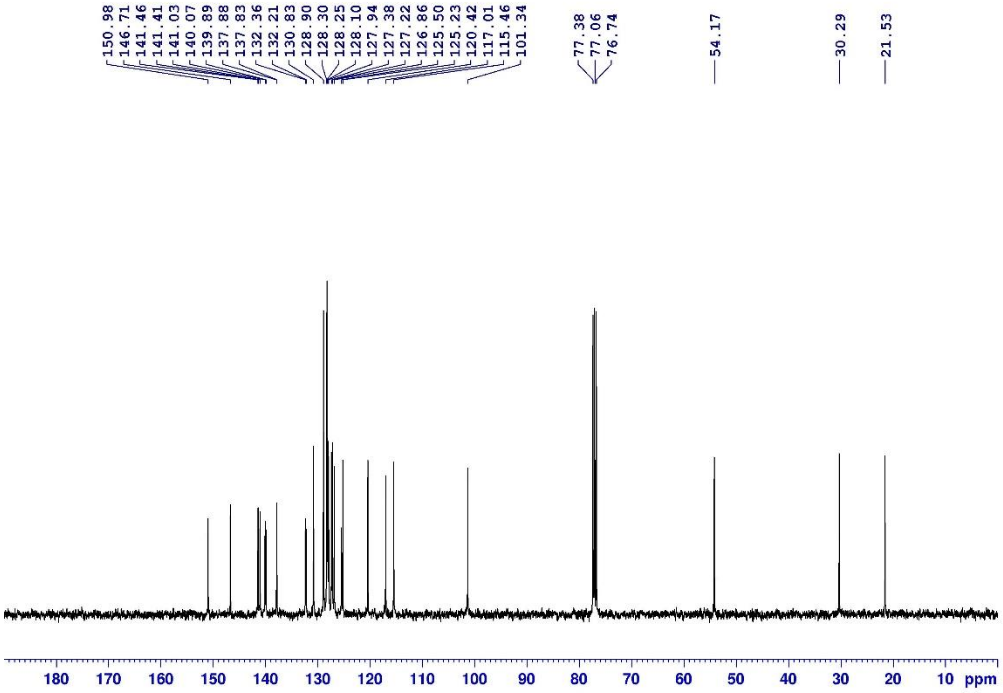

$^{1}H$ NMR (400 MHz, $CDCl_3$) Spectrum of **2n**

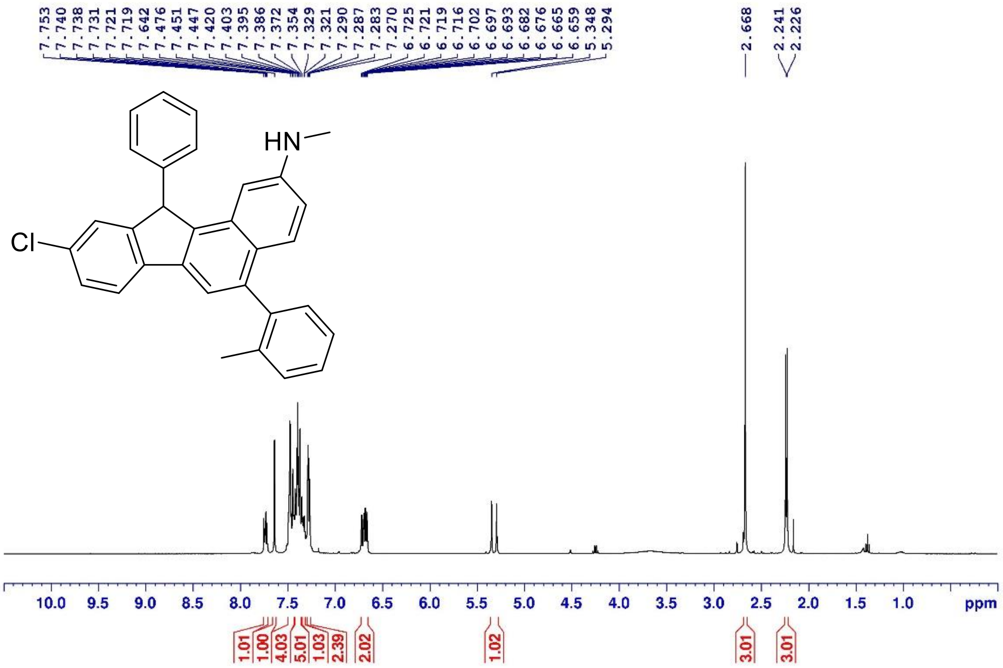


$^{13}C\{^{1}H\}$ NMR (100 MHz, $CDCl_3$) **2n**

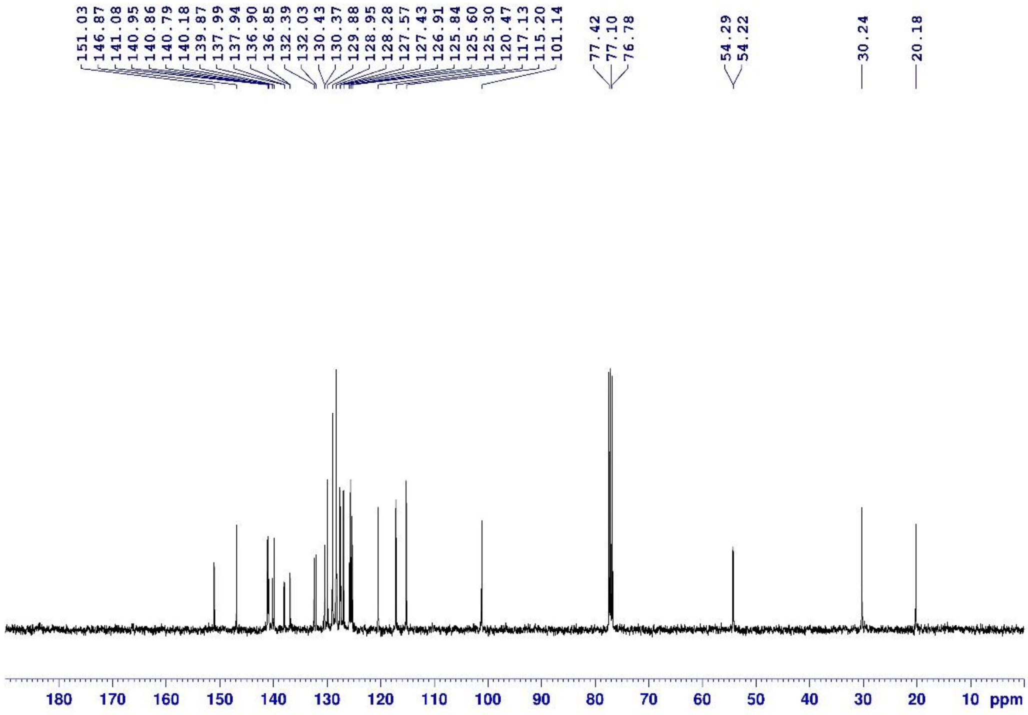

$^1$H NMR (400 MHz, $CDCl_3$) Spectrum of **2o**

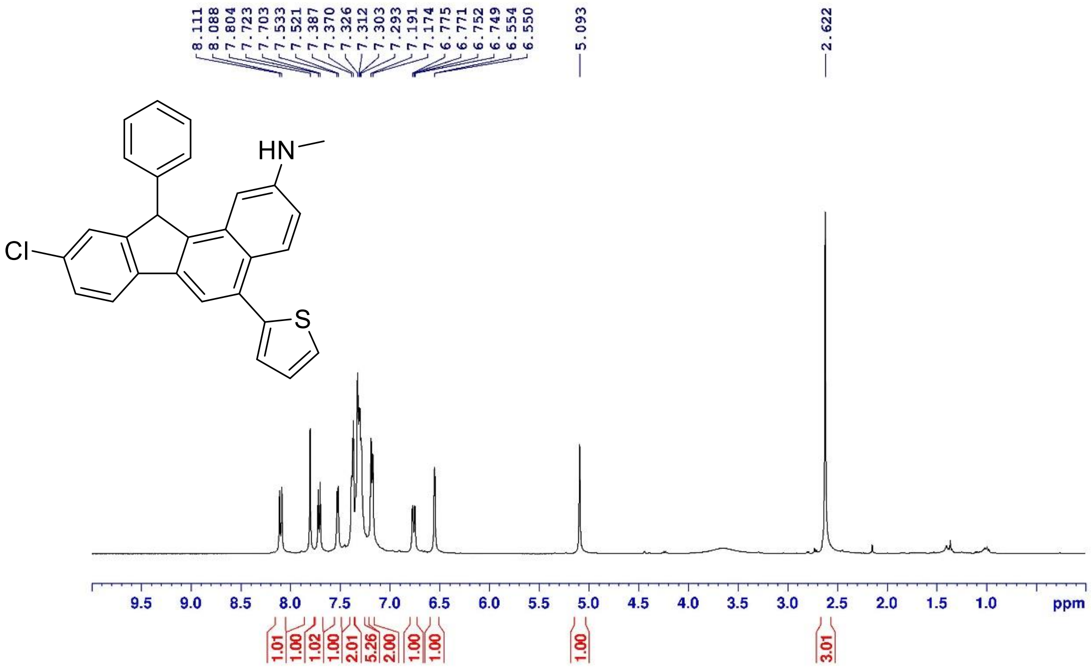


$^{13}$C{$^1$H} NMR (100 MHz, $CDCl_3$) **2o**

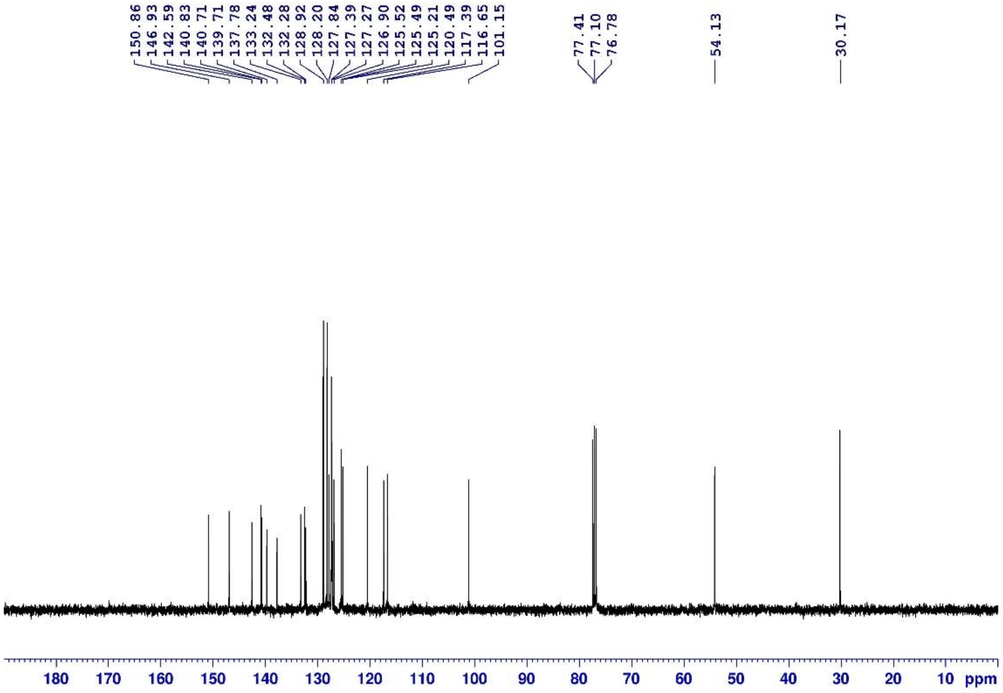

$^{1}$H NMR (400 MHz, $CDCl_3$) Spectrum of **2p**

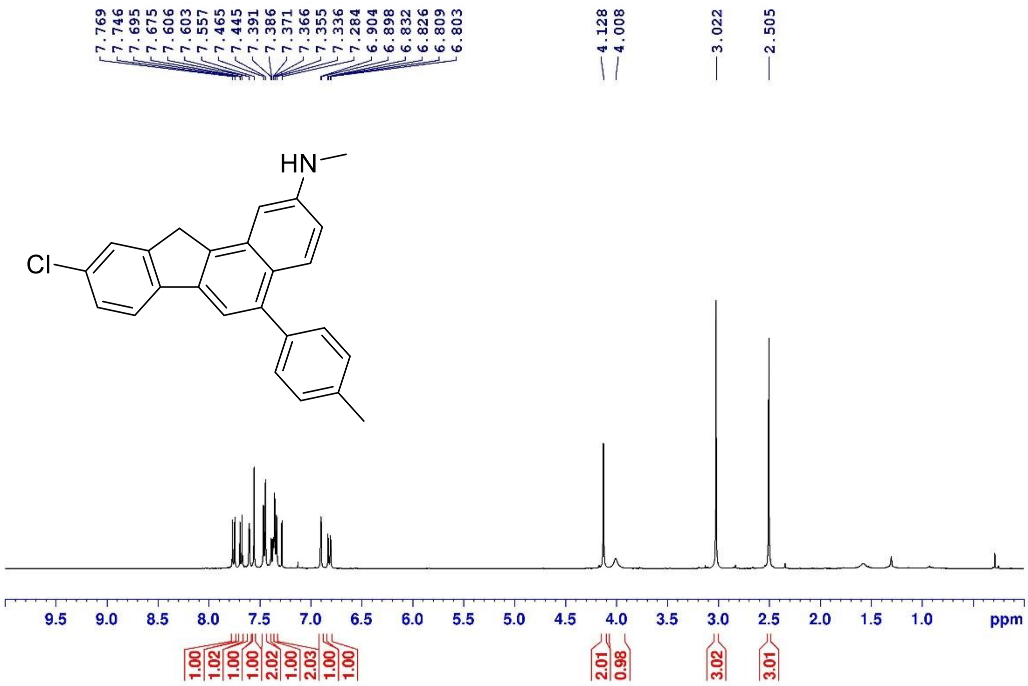

$^{13}$C{$^{1}$H} NMR (100 MHz, $CDCl_3$) **2p**

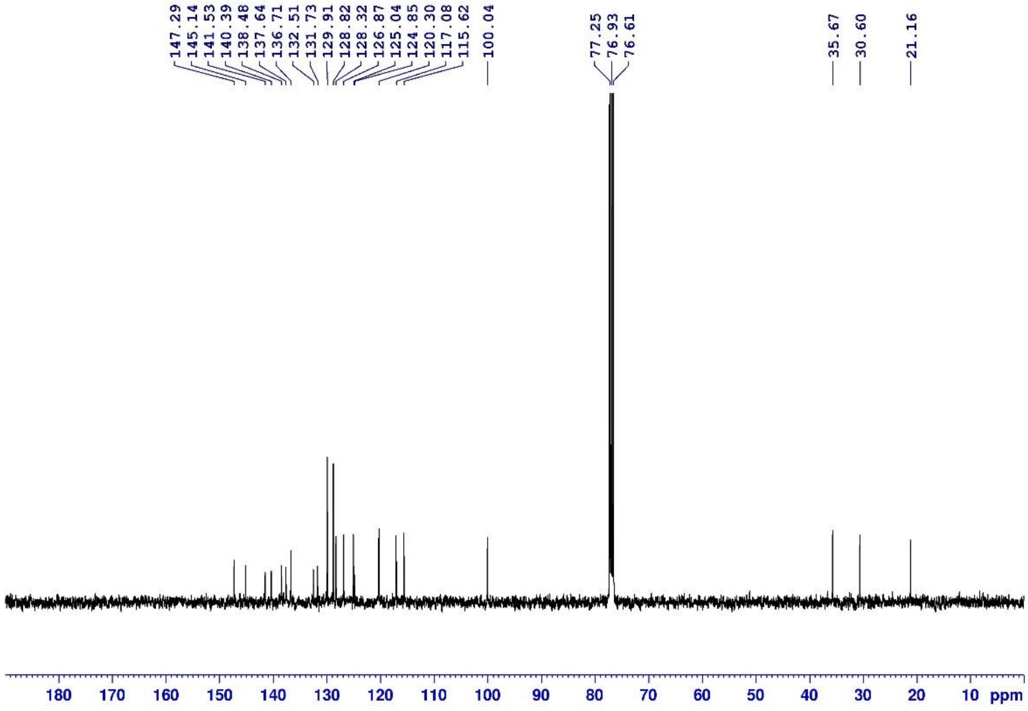

$^{1}H$ NMR (400 MHz, $CDCl_3$) Spectrum of **2r**

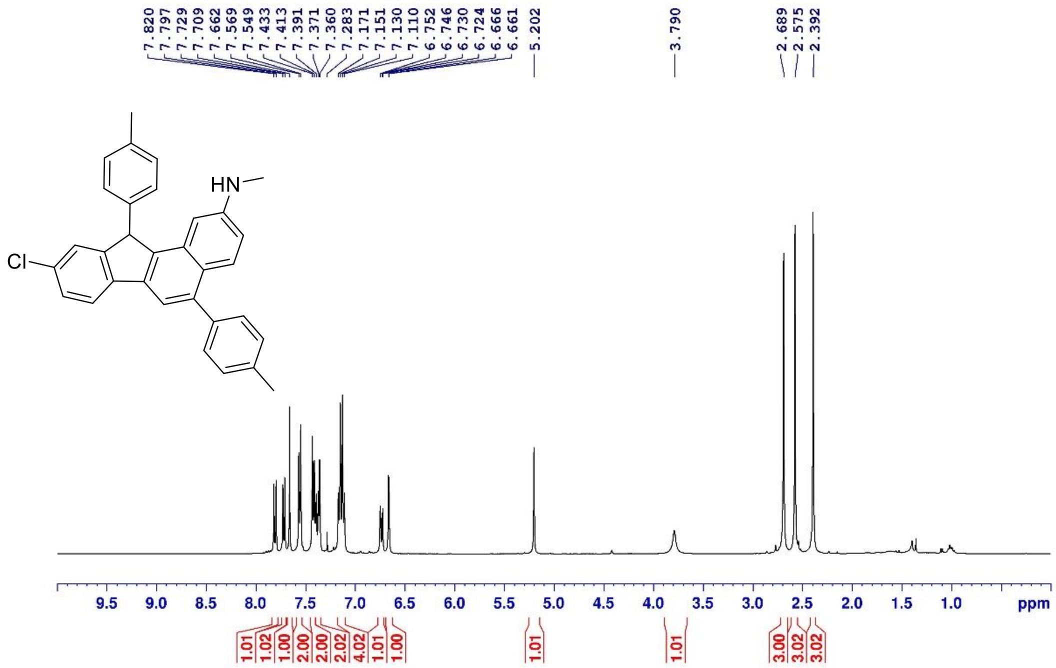


$^{13}C\{^{1}H\}$ NMR (100 MHz, $CDCl_3$) **2r**

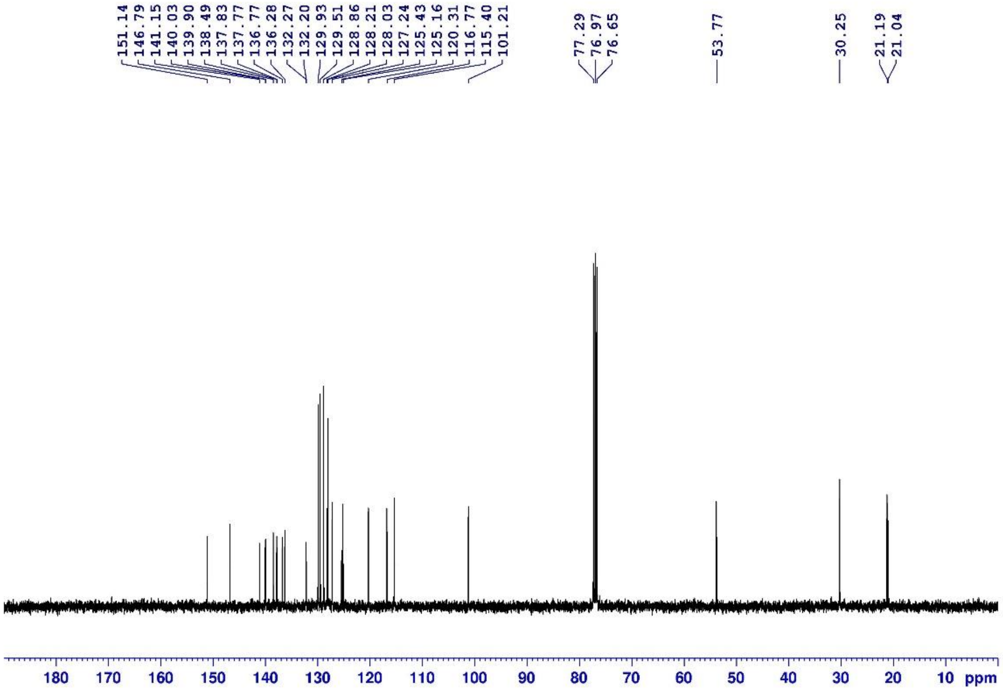

$^{1}H$ NMR (400 MHz, $CDCl_3$) Spectrum of **2s**

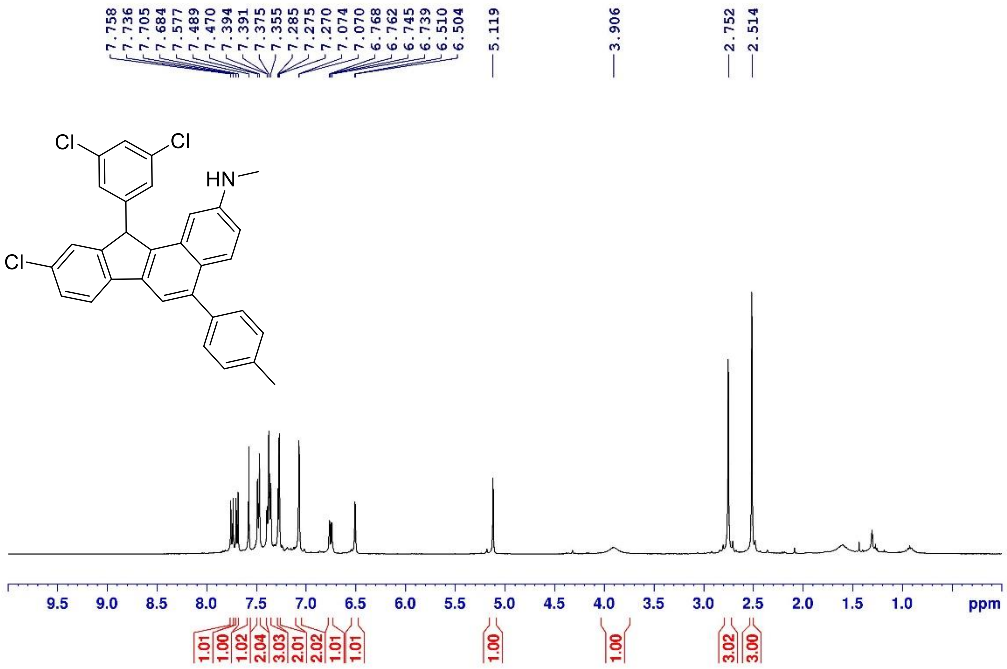


$^{13}C\{^{1}H\}$ NMR (100 MHz, $CDCl_3$) **2r**

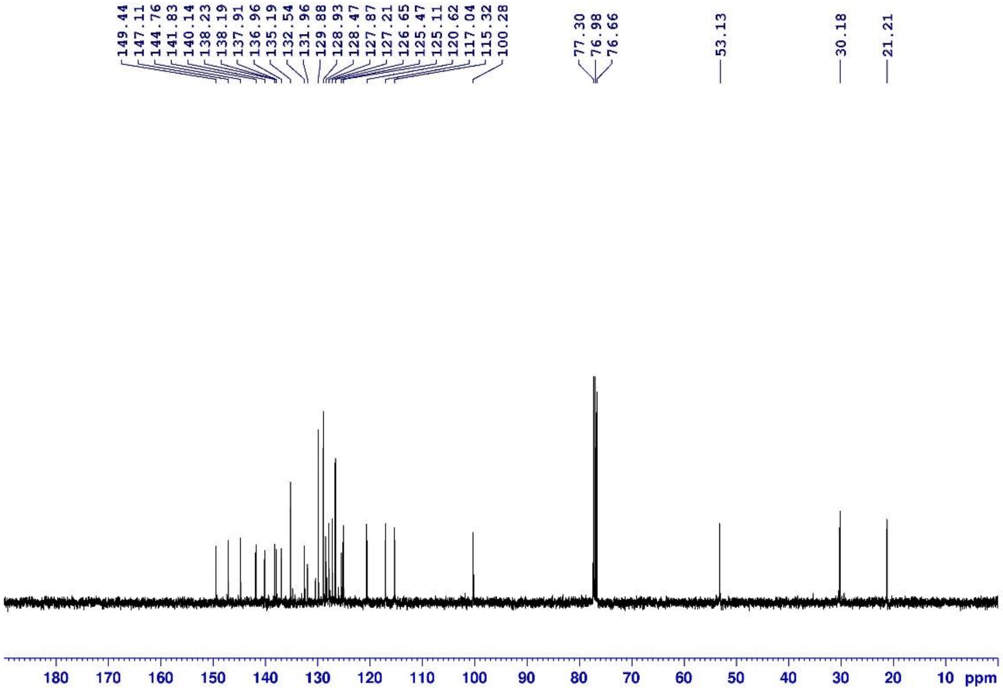

$^{1}$H NMR (400 MHz, $CDCl_3$) Spectrum of **3**

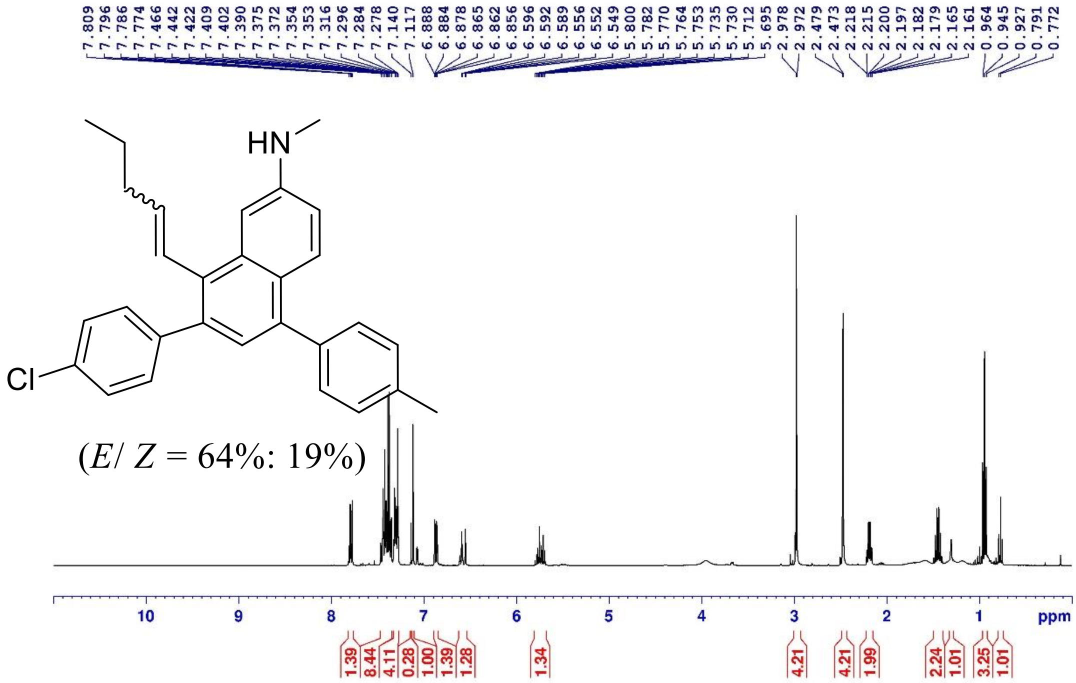


$^{13}$C{$^{1}$H} NMR (100 MHz, $CDCl_3$) **3**

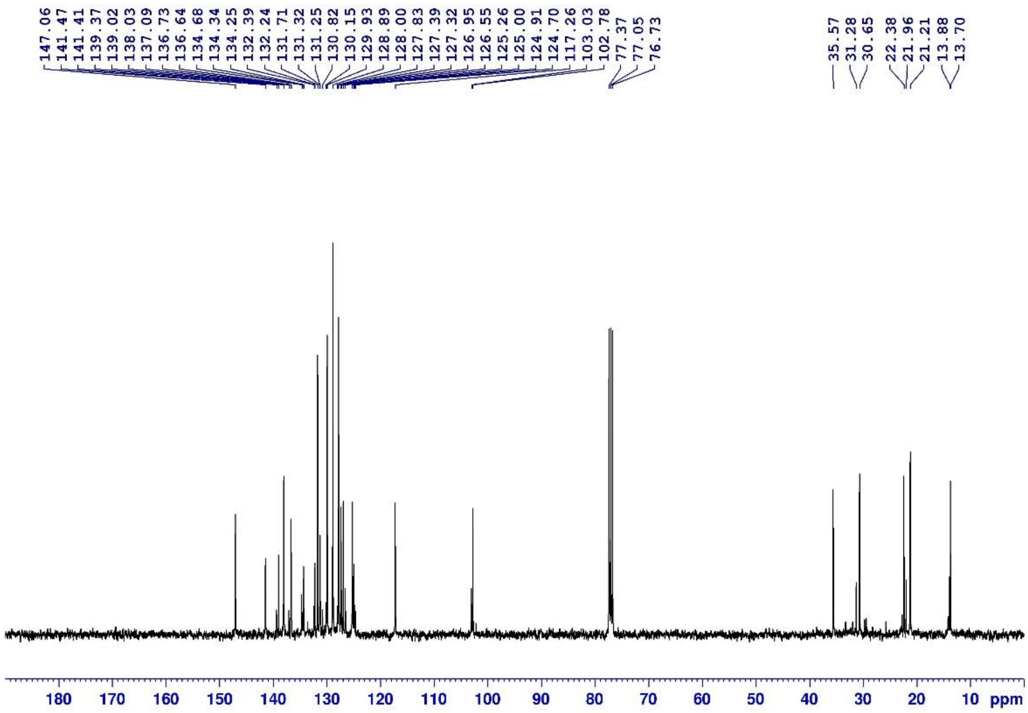

$^{1}$H NMR (400 MHz, $CDCl_3$) Spectrum of **4**

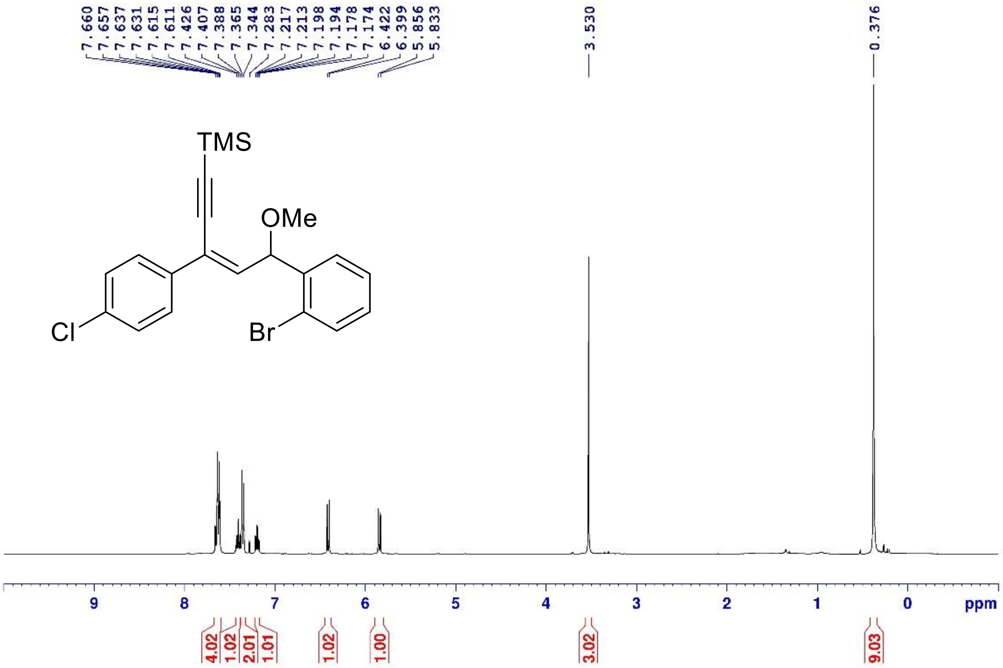


$^{13}$C{$^{1}$H} NMR (100 MHz, $CDCl_3$) **4**

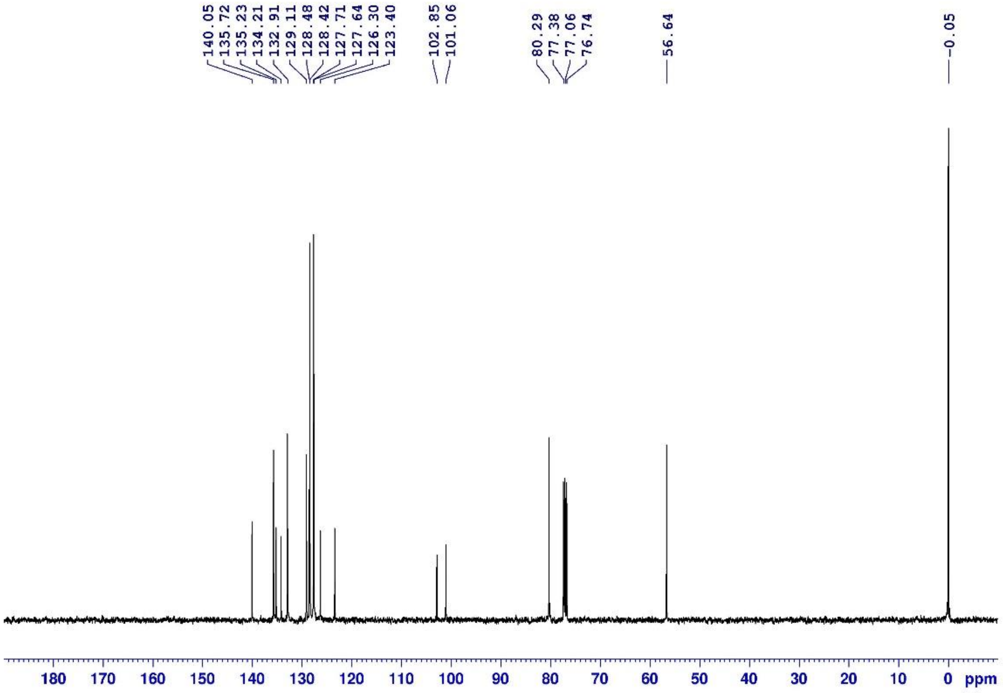

$^{1}H$ NMR (400 MHz, $CDCl_3$) Spectrum of **5**

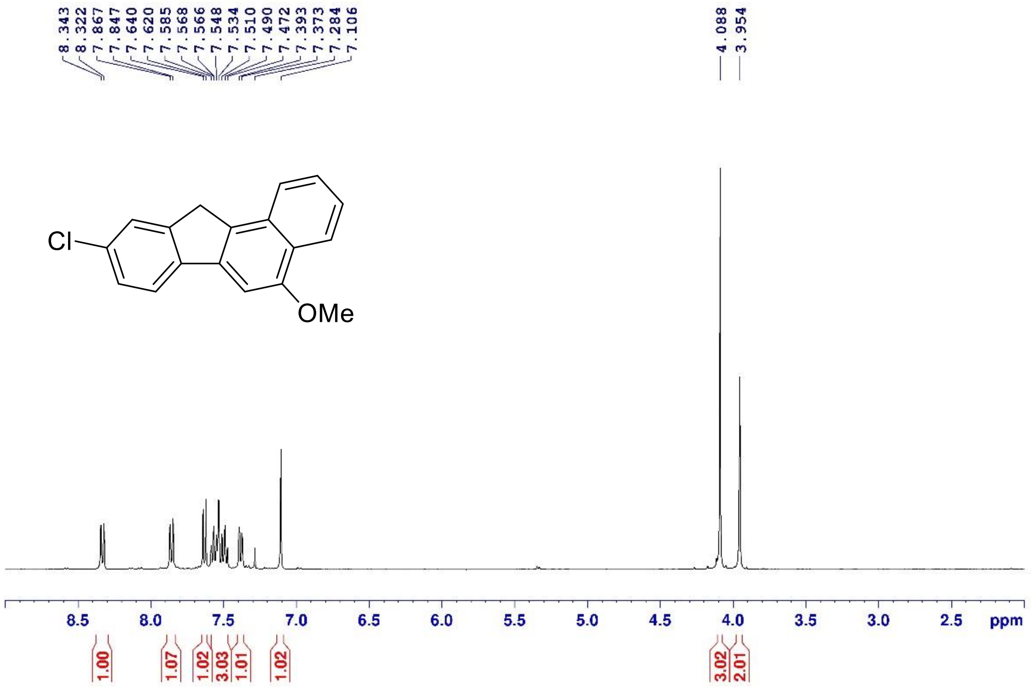


$^{13}C\{^{1}H\}$ NMR (100 MHz, $CDCl_3$) **5**

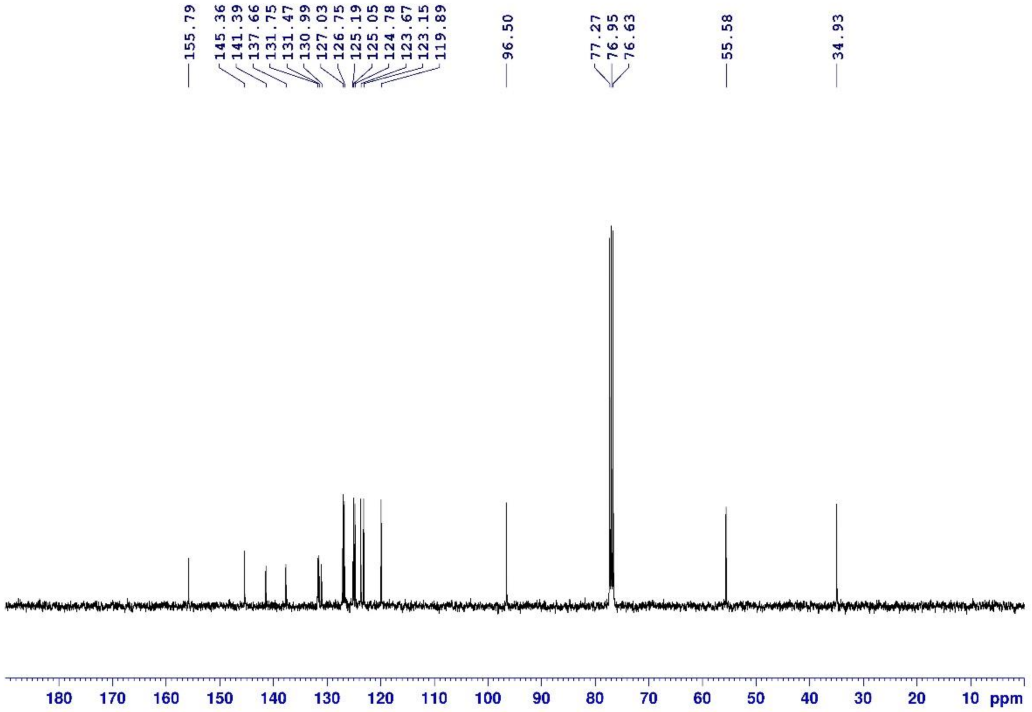

Supplement: Supplementary file 1 — jo4c01286_si_001.pdf [file jo4c01286_si_001.pdf]
